# Uncovering the Importance of Proton Donors in TmI_2_-Promoted Electron Transfer: Facile C−N Bond Cleavage in Unactivated Amides**

**DOI:** 10.1002/anie.201303178

**Published:** 2013-06-12

**Authors:** Michal Szostak, Malcolm Spain, David J Procter

**Affiliations:** School of Chemistry, University of ManchesterOxford Road, Manchester M13 9PL (UK)

**Keywords:** amides, C−N cleavage, electron transfer, lanthanides, thulium diiodide

**Figure 1:**
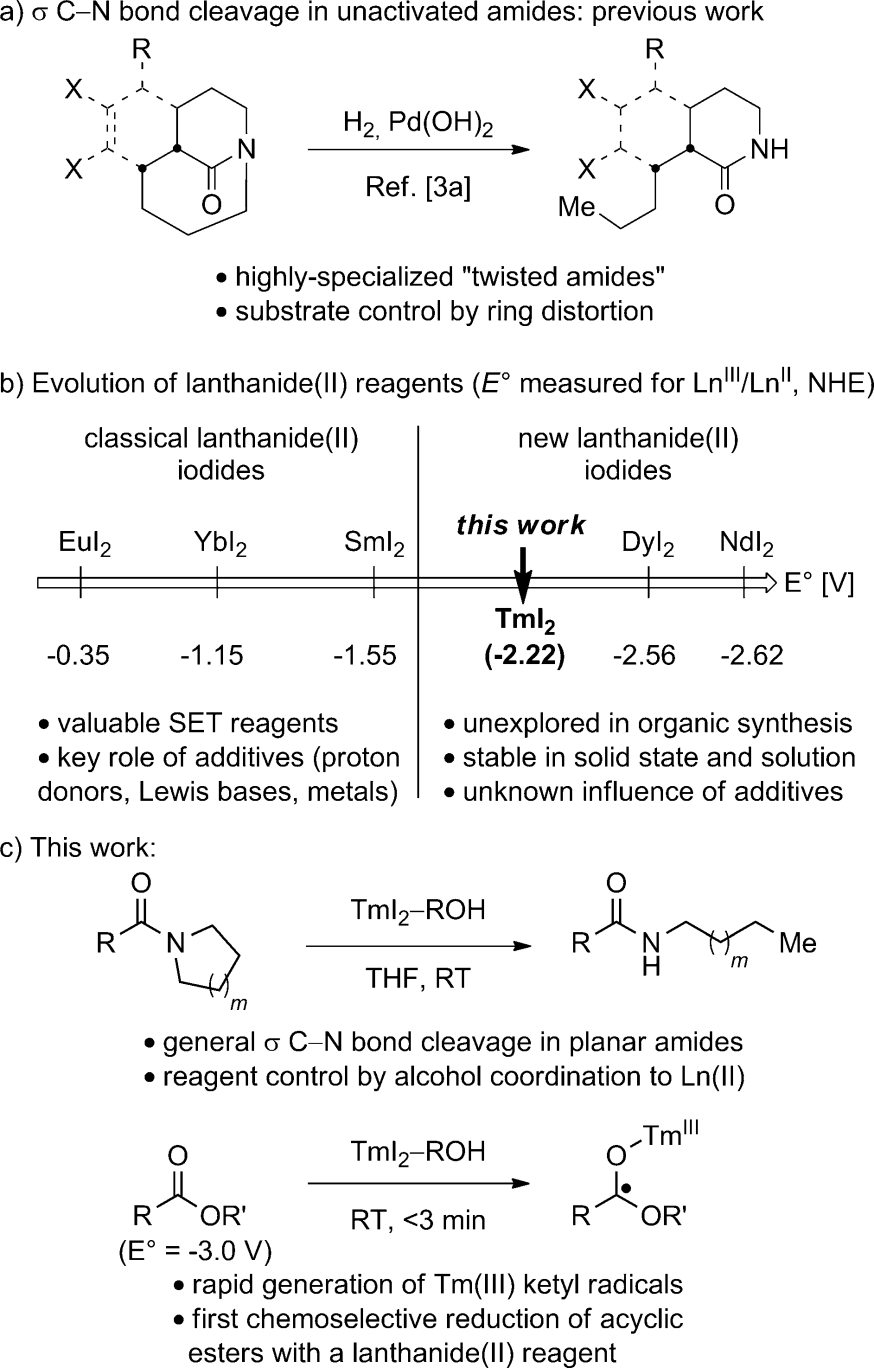
a) Cleavage of unactivated σ C−N bonds in amides. b) Classical and nonclassical lanthanide(II) iodides. c) This study.

The amide bond is one of the most ubiquitious functional groups in chemistry and biology.^1^ To date, the majority of strategies to functionalize amide bonds have focused on activation of the carbonyl group towards nucleophilic addition,^2^ however only few examples of the selective activation of σ C–N bonds in amides have been reported. In this regard, the cleavage of a σ C−N bond in amides was achieved in several highly innovative but very specialized bridged lactams, in which one of the C−N bonds was sufficiently distorted from planarity (Figure 1 a).^3^ Functionalization of the C−N bond in electronically activated phthalimides has also been described.^4^ However, a general method for the activation of σ C−N bonds in amides is unknown despite its considerable potential to advance the synthetic application of amide linkages in chemistry and biology.

The discovery of new reactivity modes of underexplored elements underpins major advancements in synthesis. In this regard, the seminal discovery of Kagan and co-workers that SmI_2_ acts as a strong electron donor^5^ has resulted in one of the most important single-electron transfer reagents in organic chemistry.^6^, ^7^ However, the inherent limitation of SmI_2_ is its relatively low redox potential (*E*° (Ln^III/II^)=−1.5 V vs. NHE),^8^ especially when compared with the extremely powerful, albeit less chemoselective, alkali metals in liquid ammonia (i.e. Birch-type reductants).^9^ Recently, nonclassical lanthanide(II) iodides (TmI_2_, thulium diiodide; DyI_2_, dysprosium diiodide; NdI_2_, neodymium diiodide) have emerged as an attractive solution to the problem of insufficient redox potential of SmI_2_ (Figure 1 b).^10^ In analogy to SmI_2_, these extremely reducing lanthanide iodides (*E*° (Ln^III/II^)=−2.2, −2.5, −2.6 V vs. NHE,^8^ respectively) have been fully characterized in ethereal solvents^11^ and can be easily obtained in multigram quantities.^12^ Seminal work by Evans et al. provided the first evidence that TmI_2_, DyI_2_, and NdI_2_ mediate challenging cross-coupling reactions beyond the scope of SmI_2_.^13^ Evans et al. also reported DyI_2_ as the first lanthanide(II) reagent capable of promoting Birch reductions under very mild conditions.11b However, the direct use of nonclassical lanthanides(II) to generate ketyl radicals has not been reported despite their significant potential to activate C—O groups that are typically resistant to open-shell reaction pathways.

Herein, we demonstrate that the TmI_2_−ROH reagent (R=H, Me), formed from the first nonclassical lanthanide(II) iodide in the series, promotes a highly unusual cleavage of the σ C−N bond in planar amides. Moreover, we report that TmI_2_−ROH is the first lanthanide(II) reagent to selectively generate ketyl radicals from aliphatic esters. Finally, we demonstrate that the presence of alcohols is critical for the formation of thermodynamically more powerful reductants from TmI_2_ (TmI_2_(ROH)_*n*_, *E*°=−2.6 V vs. SCE).

We recently developed approaches for the chemoselective reduction of cyclic esters14a and 1,3-diesters14b by using a SmI_2_−H_2_O reagent. These reactions were the first examples of the activation of carbonyls that are traditionally unreactive towards SmI_2_. On the basis of these results, we initiated efforts to chemoselectively activate other types of carbonyl by using lanthanide(II) reagents. We hypothesized that the use of the more-reducing nonclassical lanthanide(II) iodides would result in a chemoselective generation of acyl-type radicals from carboxylic acid derivatives that lie beyond the scope of SmI_2_. In particular, we considered that highly reducing nonclassical lanthanide(II) iodides that are additionally activated by proton donors, could potentially permit productive electron transfer to amide carbonyls, a functional group that has been traditionally resistant to single-electron-transfer reductants, as a result of n_N_→π*_C—O_ conjugation.^1^ With these considerations in mind, we subjected *N*,*N*-dialkyl amide **1 a** to several TmI_2_-mediated reaction conditions (Table 1). To our delight, with MeOH as the proton source, we observed efficient formation of *N*-monoalkyl amide **2 a**, in which a highly unusual cleavage of the σ C−N bond took place (Table 1, entry 3; see the Supporting Information for reagent stability studies). Control reactions demonstrated that the reaction did not proceed in the absence of a proton source (Table 1, entry 1), at low concentration of MeOH (Table 1, entry 2), with H_2_O as an alternative additive (Table 1, entry 5), and with a variety of SmI_2_ systems (Table 1, entries 6–8; see also the Supporting Information). Furthermore, the corresponding aliphatic pyrrolidinyl amine was inert to the reaction conditions (Table 1, entry 4), thus demonstrating high levels of chemoselectivity imparted by the TmI_2_ reagent.^15^

**Table 1:** Optimization of the C−N bond cleavage in unactivated amides in the presence of LnI_2_(ROH)_*n*_.

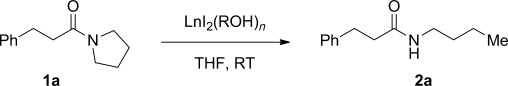						
Entry	LnI_2_	LnI_2_ (equiv)	ROH	ROH (equiv)[a]	*t*[b]	Yield [%][c]
1	TmI_2_	3	–	–	2 h	<2
2	TmI_2_	3	MeOH	10	3 min	<2
3	TmI_2_	3	MeOH	100	3 min	48 (77)[d]
4[e]	TmI_2_	3	MeOH	100	3 min	<2
5	TmI_2_	3	H_2_O	150	3 min	<2
6[f]	SmI_2_	3	–	–	3 h	<2
7[f]	SmI_2_	3	MeOH	100	3 h	<2
8[f]	SmI_2_	3	H_2_O	100	1 h	<2

[a] With respect to LnI_2_.

[b] Time elapsed until characteristic color change from Tm^II^ to Tm^III^.

[c] Determined by ^1^H NMR spectroscopy and/or GC–MS.

[d] In parentheses, yield based on the recovered starting material. TmI_2_ (6 equiv) afforded **2 a** in 45 % yield.

[e] The corresponding amine was used instead of the amide.

[f] Azetidinyl amide **1 d** used instead of the pyrrolidinyl amide. Reaction conditions: LnI_2_ (3 equiv), ROH (H_2_O, 150 equiv; MeOH, 100 equiv), THF, 23 °C. See the Supporting Information for details.

**Scheme 1:**
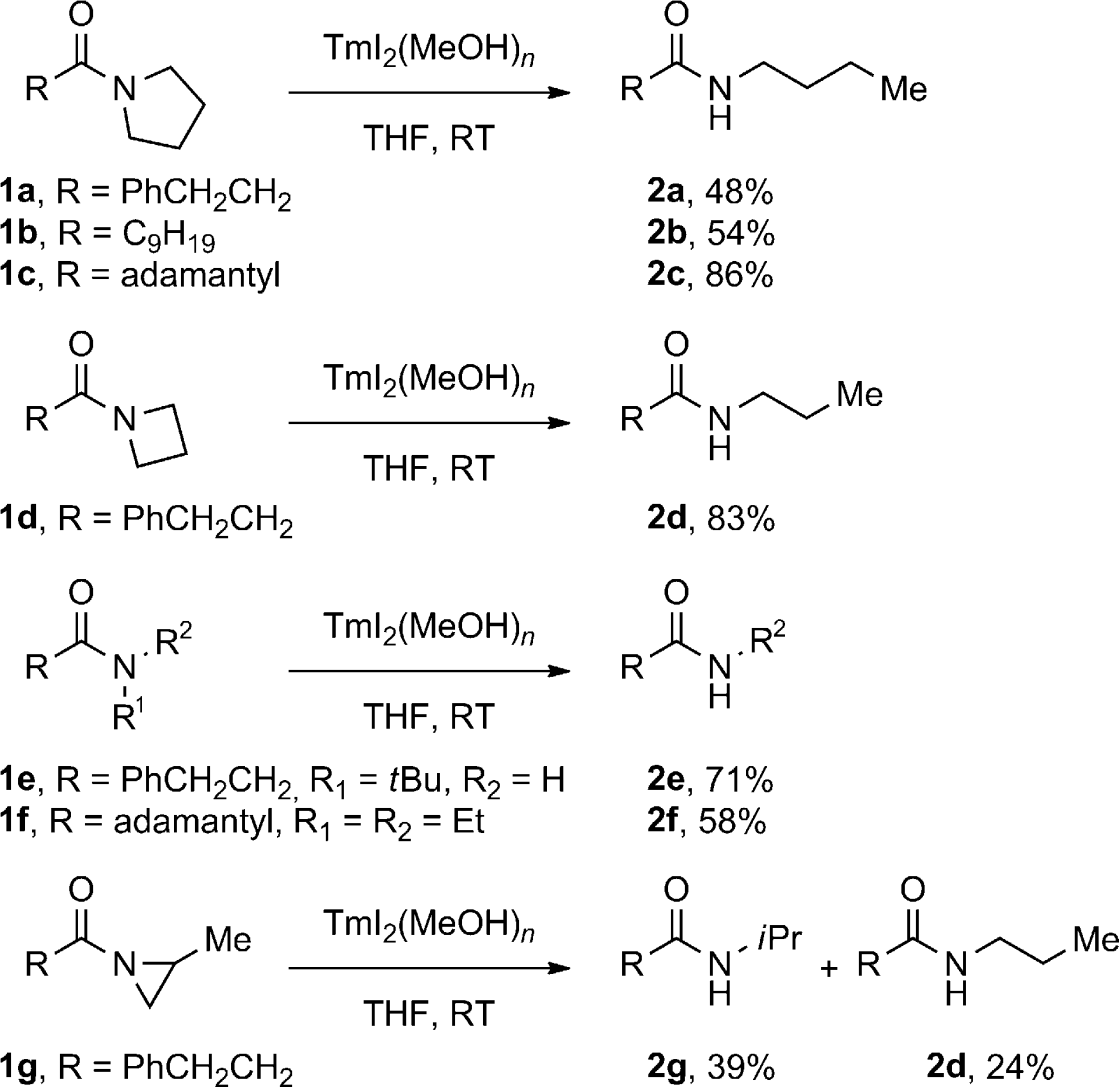
Cleavage of unactivated σ C−N bonds in amides in the presence of TmI_2_(ROH)_*n*_ at 23 °C.

With the optimized conditions in hand, a series of amides was subjected to the reaction to provide an initial examination of the scope of this transformation (Scheme 1). The C−N bond scission occurred for both unhindered and sterically encumbered pyrrolidinyl amides (**1 a**–**1 c**). Moreover, the reaction of the azetidinyl amide **1 d** demonstrated that the reaction is applicable to other cyclic amides. In addition, two acyclic amides (**1 e**–**1 f**) were similarly cleaved, thus demonstrating that the cyclic structure of amides is not necessary for the scission. Importantly, secondary *n*-alkyl and *n*-aryl amides did not undergo the cleavage reaction (see the Supporting Information), thus indicating complete selectivity of the reducing system for these tertiary amides. To gain a preliminary mechanistic insight, we subjected a sterically biased aziridinyl amide **1 g** to the reaction conditions. The reaction afforded an approximately 1.6:1.0 ratio of regioisomeric amides, with the predominant product resulting from cleavage at the less substituted carbon center. On the basis of this experiment and the known propensity of nonclassical LnI_2_ to cleave C−O bonds in ethers,^15^ we propose that the mechanism of the TmI_2_-mediated cleavage involves a direct insertion of Tm^II^ into the C−N amide bond; however, a mechanism involving fragmentation of an initially-formed ketyl-type radical seems also to be operating in some cases as suggested by the correlation of the reaction efficiency with thermochemical stabilization energies (SE) of the fragmenting radical in the series: *t*Bu (71 %, SE=4.35 kcal mol^−1^)>*i*Pr (29 %, SE=2.57 kcal mol^−1^)>Me (<2 %, SE=−1.65 kcal mol^−1^).^16^

The mechanistic implications of the C−N cleavage merit further discussion. The present reaction with TmI_2_ represents the first case of a general scission of unactivated σ C−N bonds in planar amides, and compares favorably with the previous examples of the cleavage of a σ C−N bond in distorted lactams^3^ (reagent vs. substrate control). Moreover, it strongly suggests that the reactivity of nonclassical lanthanides(II) extends beyond being the reagents that simply close the energy gap between SmI_2_ and the Birch-type reductants.^11^, 13a

Having established that TmI_2_−ROH is capable of an efficient electron transfer to the amide carbonyl group but not their reduction, the reagent system was applied to the generation of ketyl radicals from esters (Table 2). In previous work, we reported the reduction of lactones in the presence of SmI_2_−H_2_O;14a however, this reaction suffered from long reaction times, was limited to unhindered substrates, and could be applied only to six-membered lactones; other ring systems and acyclic esters were unreactive under the reaction conditions. In sharp contrast, TmI_2_−ROH reacted with a wide range of substrates, including lactones (Table 2, entry 1), aliphatic (Table 2, entries 2 and 3), aromatic (Table 2, entries 3 and 4), alpha-substituted (Table 2, entries 4–6), and sterically demanding (Table 2, entry 7) esters. In all cases rapid (within 2–3 min) reduction to the corresponding alcohols took place, clearly demonstrating the higher reactivity of TmI_2_−ROH. Control reactions established that, in the absence of proton donors, TmI_2_ does not reduce aliphatic esters. Acids are not reduced under the reaction conditions (Table 2, entry 8), thus opening the door for highly chemoselective reductions of carboxylic acid derivatives through single-electron reaction pathways that are not possible with the traditional alkali or transition metal hydrides.^9^ Overall, this study outlines the reactivity scale for the generation of ketyl-type radicals with TmI_2_−ROH (see the Supporting Information for comparison tables between TmI_2_ and SmI_2_), demonstrates that useful levels of chemoselectivity are possible with TmI_2_−ROH, and opens the door for the use of TmI_2_-generated ketyls in radical bond-forming reactions.

**Table 2:** Reduction of aliphatic esters in the presence of TmI_2_(ROH)_*n*_ at 23 °C.^[a]^

Entry	Ester/Acid	ROH	*t* [min][a]	Yield [%][b]
1	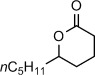	H_2_O	2–3	88
2	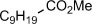	MeOH	2–3	99
3	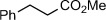	MeOH	2–3	96
4	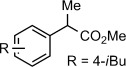	MeOH	2–3	85
5	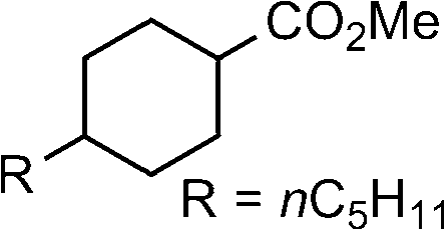	MeOH	2–3	94
6	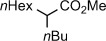	MeOH	2–3	63
7	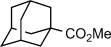	MeOH	2–3	58
8	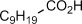	MeOH	2–3	<5[c]

[a] Time elapsed until characteristic color change from Tm^II^ to Tm^III^.

[b] Determined by ^1^H NMR spectropscopy.

[c] Decanoic acid recovered in >95 %. Reaction conditions: TmI_2_ (6–8 equiv), ROH (H_2_O, 150 equiv; MeOH, 100 equiv), THF, 23 °C. See the Supporting Information for details.

To gain a preliminary mechanistic insight into the key effect of protic additives on the properties of the TmI_2_ reagent (note that in both cases no reaction was observed with TmI_2_ alone, see the Supporting Information), we examined the reactivity of TmI_2_ with a set of aromatic hydrocarbons with gradually increasing redox potentials in the presence of MeOH (Table 3).^17^ In this study, the TmI_2_−MeOH complex was found to reduce aromatic hydrocarbons with redox potentials up to −2.6 V (vs. SCE); however, benzene was inert under the reaction conditions. These results suggest that the addition of MeOH to TmI_2_ results in an increase of the reduction potential of TmI_2_ by approximately 0.6 V.13d

**Table 3:** Determination of the redox potential of TmI_2_(ROH)_*n*_ by reduction of aromatic hydrocarbons.

Entry	Hydrocarbon	−*E*_1/2_ [V][a]	Reaction with TmI_2_ observed[c]
1	cyclooctatetraene[b]	1.83	+
2	anthracene	1.98	+
3	stilbene	2.21	+
4	1,4-diphenylbenzene	2.40	+
5	1,3,5-triphenylbenzene	2.51	+
6	naphthalene	2.61	+
7	styrene	2.65	+
8	benzene	3.42	−

[a] In volts vs. SCE; *E*_1/2_ describes half-reduction potential; see Ref. 17.

[b] Ref. 13d.

[c] Determined by GC and/or ^1^H NMR spectroscopy.

Furthermore, deuterium incorporation and kinetic isotope effect studies in the reduction of stilbene, a reaction that is known to proceed through an outer-sphere electron-transfer mechanism,^18^ using TmI_2_−ROH ([D_4_]methanol, 96.5 % D_2_ incorporation, *k*_H_/k_D_=1.13±0.1; D_2_O, 98.0 % D_2_ incorporation, *k*_H_/k_D_=1.27±0.1), suggest that the increase in reduction potential of the reagent results from complexation between the proton donor and TmI_2_.

A detailed examination of different proton donors in the model system (see the Supporting Information) revealed that a much lower concentration of alcohols (10 equiv) is required to enhance the redox potential of TmI_2_ in comparison with SmI_2_ (100 equiv).^19^ This result is consistent with the smaller radial size of Tm^II^ and bodes well for the development of catalytic cycles based on regeneration of the TmI_2_ reagent.^20^

**Scheme 2:**
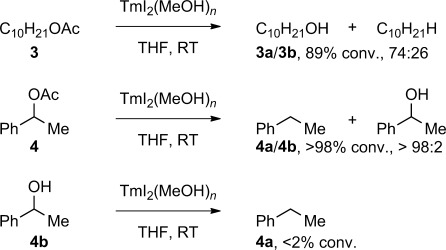
Investigating the mechanism of ester reduction with TmI_2_(ROH)_*n*_.

Finally, to test whether in analogy to amides a bond cleavage mechanism also contributes to the reduction of esters with TmI_2_, we subjected decyl and 1-phenylethyl acetate to the reaction conditions (Scheme 2). C−O bond scission was the minor pathway in the case of **3** and the predominant one in the case of **4**; these results indicate that the cleavage is also operating in the ester reduction and provides a unifying reactivity model for the TmI_2_-mediated electron transfer.13a

In summary, the highly unusual cleavage of unactivated σ C−N bonds in amides in the presence of TmI_2_, the first nonclassical lanthanide(II) iodide in the series (TmI_2_, DyI_2_, NdI_2_), has been achieved. This method was also applied to the first chemoselective reduction of esters with any lanthanide(II) reagent.^21^ Initial mechanistic studies suggest that proton donors play a key role in activating the reagent^22^ and that Tm^III^-bound ketyl radicals are more stable than the corresponding Sm^III^ ketyls.^23^ We fully expect that this work will serve as a platform to enable discovery of novel electron-transfer processes based on nonclassical lanthanide(II) iodides.
