# Supplementary material for: Uncovering the Importance of Proton Donors in TmI2-Promoted Electron Transfer: Facile C−N Bond Cleavage in Unactivated Amides
Source: Angew Chem Int Ed Engl. 2013 Jun 12;52(28):7237–41. doi: 10.1002/anie.201303178 (PMC4265963; doi:10.1002/anie.201303178)
Supplement: Supplementary file 1 [file anie0052-7237-sd1.pdf]

Angewandte Chemie
Eine Zeitschrift der Gesellschaft Deutscher Chemiker

Supporting Information

© Wiley-VCH 2013

69451 Weinheim, Germany

Uncovering the Importance of Proton Donors in TmI_2-Promoted Electron Transfer: Facile C–N Bond Cleavage in Unactivated Amides**

Michal Szostak, Malcolm Spain, and David J. Procter**

anie_201303178_sm_miscellaneous_information.pdf

Supplementary Information

Table of Contents

Table of Contents 1
List of Known Compounds/General Methods 2
Experimental Procedures and Characterization Data 3
- General Procedures 3
- Control Reactions using TmI_2–H_2O and TmI_2–MeOH 3
- C–N Bond Cleavage in Amides 4
- Reduction of Esters and Carboxylic Acids 8
- Mechanistic Studies 11
    - Determination of the Redox Potential of TmI_2–MeOH 11
    - Detailed Investigation of the Role of Proton Donors 11
    - Deuterium Incorporation and Kinetic Isotope Effect Studies 14
    - Studies on the Origin of Selectivity in Ester Reduction 15

Comparison of Reactivity of SmI_2–ROH and TmI_2–ROH 16
References 18
^{1}H and ^{13}C NMR Spectra 19

Corresponding Author:

Dr. Michal Szostak
Professor David J. Procter
School of Chemistry
University of Manchester
Oxford Road
Manchester, M13 9PL
United Kingdom

General Methods

All experiments were performed using standard Schlenk or glovebox techniques under argon atmosphere unless stated otherwise. Thulium(II) iodide powder (anh., 99.9+%; ICP-MS Full Trace Analysis, purity >99.9+%; Assay: 40.1% Tm, 60.5% I) was purchased from Aldrich and stored in an argon containing glove box (< 1 ppm of O_2). All solvents were purchased at the highest commercial grade and used as received or after purification by passing through activated alumina columns or distillation from sodium/benzophenone under nitrogen. All solvents were deoxygenated by freeze-pump-thawing with argon prior to use. All other chemicals were purchased at the highest commercial grade and used as received. Reaction glassware was oven-dried at 140 °C for at least 24 h or flame-dried prior to use, allowed to cool under vacuum and purged with argon (three cycles). All products and starting materials used in this study are commercially available and/or have been previously reported. ^{1}H NMR and ^{13}C NMR spectra were recorded in $CDCl_3$ on Bruker spectrometers at 300, 400 and 500 MHz (^{1}H NMR) and 75, 100 and 125 MHz (^{13}C NMR). All shifts are reported in parts per million (ppm) relative to residual $CHCl_3$ peak (7.27 and 77.2 ppm, ^{1}H NMR and ^{13}C NMR, respectively). All coupling constants (J) are reported in hertz (Hz). Abbreviations are: s, singlet; d, doublet; t, triplet; q, quartet; br s, broad singlet. GC-MS chromatography was performed using Agilent 7890A GC System and Agilent 5975C inert XL EI/CI MSD with Triple Axis Detector equipped with Agilent HP-5MS column (19091S-433) (length 30 m, internal diameter 0.25 mm, film 0.25 μm) using helium as the carrier gas at a flow rate of 1 mL/min and an initial oven temperature of 40 °C or 50 °C. The injector temperature was 250 °C. The detector temperature was 250 °C. For runs with the initial oven temperature of 40 °C, temperature was increased with a 15 °C/min ramp after 40 °C hold for 3 min to a final temperature of 300 °C, then hold at 300 °C for 5 min (splitless mode of injection, total run time of 25.33 min). For runs with the initial oven temperature of 50 °C, temperature was increased with a 25 °C/min ramp after 50 °C hold for 3 min to a final temperature of 300 °C, then hold at 300 °C for 5 min (splitlesss mode of injection, total run time of 18 min). GC chromatography was performed using DANI Master GC Fast Gas Chromatograph System equipped with Varian VF-1m column (length 30 m, internal diameter 0.25 mm, film 0.25 μm) using hydrogen as the carrier gas at a flow rate of 1 mL/min and an initial oven temperature of 40 °C or 70 °C. The injector temperature was 250 °C. The detector

temperature was 250 °C. The temperature was increased with a 10 °C/min ramp to a final temperature of 150 °C or 220 °C (splitless mode of injection). For runs with the initial oven temperature of 40 °C, temperature was increased by 10 °C/min after 40 °C hold for 3 min (total run time of 13 min). For runs with the initial oven temperature of 70 °C, temperature was increased by 10 °C/min with no hold time (total run time of 15 min).

Experimental Procedures and Characterization Data

General Procedures

In an argon-filled glove box, TmI_2 was weighted out into a vial equipped with a magnetic stir bar, transferred out of the glove box, and placed under a positive pressure of argon using standard Schenk techniques. Substrate (as a THF solution) was added, followed by a rapid injection of alcohol, with vigorous stirring. After the specified time, the excess of TmI_2 was oxidized by bubbling air through the reaction mixture. The reaction mixture was diluted with CH_2Cl_2 (20 mL) and HCl (1.0 *N*, 30 mL). The aqueous layer was extracted with CH_2Cl_2 (3 x 20 mL), organic layers were combined, dried over Na_2SO_4, filtered and concentrated. All products were identified using ^1H NMR (400 and 500 MHz) analysis and comparison with authentic samples. GC, GC/MS analysis was used for volatile products. In all cases, GC, GC/MS or LC/MS analysis was used as a complementary method of analysis to confirm the product distribution. All yields refer to yields determined by ^1H NMR using an internal standard unless stated otherwise. Reactions involving lanthanides(II) can typically be followed by visual observation of the changes in color of the respective reaction mixtures.[1] In the case of $TmI_2(THF)_n$, the color changes from Tm^{II} (dark green) to Tm^{III} (oxidized, solvated: transparent color; oxidized, w/o protic additives: yellow color).[2] It should be noted that the instability of $TmI_2(THF)_n$ to light has been reported; it is recommended that the reactions involving $TmI_2(THF)_n$ are carried out in the dark.[3]

Control Reactions using TmI_2–H_2O and TmI_2–MeOH

TmI_2–H_2O. An oven-dried vial equipped with a magnetic stir-bar was charged with TmI_2 (0.10 mmol) and 10.0 mL of THF, which resulted in a dark green solution of $TmI_2(THF)_n$. To a 1.0 mL of the stock solution of TmI_2 in THF prepared as described above, water (neat) was slowly added

dropwise. Upon addition of ca. 50 μL of H_2O a fleeting red color was observed, indicating the formation of a saturated TmI_2–H_2O complex.[4] *TmI_2–MeOH.* According to the above procedure, methanol (50 μL) was added to a 1.0 mL of the stock solution of TmI_2 in THF prepared as described above and the solution was stirred until the dark green color gradually faded to give a transparent solution of solvated Tm^{III}.[5,6] Upon prolonged exposure to air, the transparent color characteristic of solvated Tm^{III} species (with either H_2O or MeOH) slowly changes into dark yellow.[5,6]

C–N Bond Cleavage in Amides

According to the general procedure, amide as indicated in Table SI-5 (typically, 0.05 mmol) was reacted with thulium(II) iodide (0.15-0.30 mmol) and a proton source in THF (typically, 1.0 mL) until decolorization to transparent indicative of formation of Tm^{III} species had occurred. All compounds have been previously reported.

Table SI-1. C–N Bond Cleavage in Amides using TmI_2–ROH at 23 °C – Optimization Study.

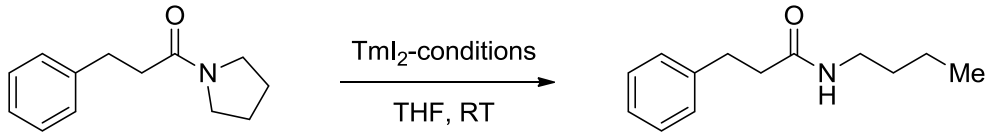

1a **2a**

entry	TmI_2 (equiv)	ROH	ROH (equiv)	time[a]	yield[b] (%)
1[c]	3	-	-	2 h	<2
2	3	MeOH	10	2-3 min	-
3	3	MeOH	100	2-3 min	48 (77)[d]
4	3	H_2O	150	2-3 min	<2
5[e]	3	MeOH	100	2-3 min	-

All reactions carried out using standard Schlenk techniques. Conditions: under argon, to TmI_2, substrate (0.05 mmol) in THF (typically, 1 mL) was added, followed by a proton source. Conversion = (100-SM). Yield refers to C–N bond cleavage product. [a]Indicates time after which decolorization from deep green (Tm^{II}) to transparent (Tm^{III}) had occurred. [b]Determined by ^1H NMR and/or GC-MS analysis by comparison with authentic samples. [c]Reaction carried out in the dark. [d]In parentheses, yield based on the recovered starting material. TmI_2 (6 equiv) afforded **2a** in 45% yield. [e]1-(3-Phenylpropyl)pyrrolidine used instead of the amide.

To investigate whether SmI_2 promotes a similar cleavage of the C–N bond in amides, we selected azetidinyl amide **1d** on the basis of its higher reactivity under the reaction conditions with TmI_2–MeOH. To provide reaction conditions with an absolute exclusion of oxygen, SmI_2 powder (AAPL, stored and dispensed in a glovebox) was used for these control reactions. However, under the tested reaction conditions, the C–N bond cleavage product **2d** was not observed (Table SI-2).

Table SI-2. C–N Bond Cleavage in Amides using SmI_2–ROH at 23 °C – Control Reactions.[a]

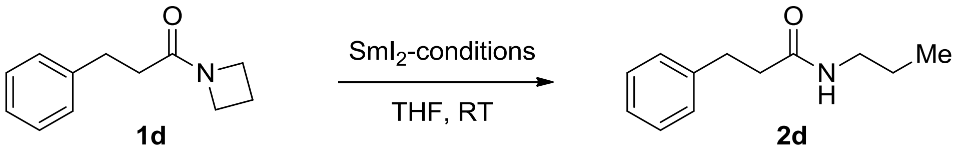

entry	SmI_2 (equiv)	ROH	ROH (equiv)	time[b]	conversion[c] (%)	yield[c] (%)
1	3	-	-	3 h	<2	<2
2	3	MeOH	100	3 h	<2	<2
3	3	H_2O	100	1 h	<2	<2

[a]See, Table SI-1. [b]Indicates the time after which decolorization from dark blue (SmI_2), dark brown (SmI_2–MeOH) or red wine (SmI_2–H_2O) to transparent (Sm^{III}) had occurred. [c]Determined by ^1H NMR and/or GC-MS.

To further investigate a potential mechanism of the C–N bond cleavage reaction, we subjected a set of primary amides with a varying degree of steric and electronic substitution at the nitrogen atom to the reaction conditions (Table SI-3). In the event, we found that the efficiency of the reaction could be correlated with thermochemical stabilization energy of the fragmenting radical,[7] which possibly could indicate that a mechanism based on an initially-formed ketyl radical, with a subsequent radical fragmentation is also operating in these cases. Further studies on the mechanism, full scope of this transformation, and its intriguing application to the chemistry of bridged lactams[8] will be reported separately.

Table SI-3. C–N Bond Cleavage of Amides using TmI_2–ROH at 23 °C – Correlation Study.[a]

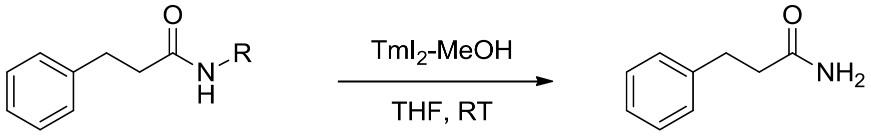

entry	R	TmI_2 (equiv)	ROH (equiv)	conversion[b] (%)	yield[b] (%)	SE[c] (kcal/mol)
1	*t*-Bu	3	100	>95	71	4.35
2	*i*-Pr	3	100	52	29	2.57
3	Me	3	100	33	<2	-1.67
4	Ph	3	100	23	<2	-10.27

[a]See, Table SI-1. [b]Determined by ^{1}H NMR and/or GC-MS. [c]SE = Thermochemical stabilization energy of the corresponding radical (*t*-Bu, *i*-Pr, Me, Ph) as defined by Ref. 7.

3-Phenyl-1-(pyrrolidin-1-yl)propan-1-one (1a). ^{1}H NMR (500 MHz, $CDCl_3$) δ 1.79-1.92 (m, 4H), 2.58 (t, *J* = 7.5 Hz, 2H), 3.0 (t, *J* = 8.0 Hz, 2H), 3.30 (t, *J* = 7.0 Hz, 2H), 3.47 (t, *J* = 7.0 Hz, 2H), 7.19-7.26 (m, 3H), 7.28-7.32 (m, 2H); ^{13}C NMR (125 MHz, $CDCl_3$) δ 24.4, 26.1, 31.2, 36.8, 45.7, 46.6, 126.1, 128.5, 128.5, 141.5, 170.9.

1-(3-Phenylpropyl)pyrrolidine (1a-SI). ^{1}H NMR (300 MHz, $CDCl_3$) δ 1.81 (m, 4H), 1.85-1.95 (m, 2H), 2.47-2.55 (m, 6H), 2.68 (t, *J* = 7.8 Hz, 2H), 7.17-7.24 (m, 3H), 7.27-7.34 (m, 2H); ^{13}C NMR (75 MHz, $CDCl_3$) δ 23.5, 30.7, 34.0, 54.2, 56.1, 125.7, 128.3, 128.4, 142.3.

1-(Pyrrolidin-1-yl)decan-1-one (1b). ^{1}H NMR (500 MHz, $CDCl_3$) δ 0.72 (t, *J* = 7.5 Hz, 3H), 1.05-1.21 (m, 12H), 1.45-1.52 (m, 2H), 1.65-1.73 (m, 2H), 1.75-1.82 (m, 2H), 2.10 (t, *J* = 7.5 Hz, 2H), 3.26 (t, *J* = 6.5 Hz, 2H), 3.30 (t, *J* = 6.5 Hz, 2H); ^{13}C NMR (125 MHz, $CDCl_3$) δ 14.1, 22.6, 24.4, 24.9, 26.1, 29.3, 29.4, 29.5, 31.8, 34.8, 45.5, 46.6, 171.8.

Adamantan-1-yl(pyrrolidin-1-yl)methanone (1c). ^{1}H NMR (500 MHz, $CDCl_3$) δ 1.35-1.43 (m, 6H), 1.44-1.58 (m, 4H), 1.64-1.68 (m, 6H), 1.68-1.72 (m, 3H), 3.05-3.42 (m, 4H); ^{13}C NMR (125 MHz, $CDCl_3$) δ 27.3, 28.3, 36.6, 38.1, 41.6, 47.9, 175.7.

1-(Azetidin-1-yl)-3-phenylpropan-1-one (1d). ^{1}H NMR (500 MHz, $CDCl_3$) δ 1.99-2.06 (m, 2H), 2.22 (t, *J* = 7.5 Hz, 2H), 2.82 (t, *J* = 7.5 Hz, 2H), 3.78 (t, *J* = 7.5 Hz, 2H), 3.86 (t, *J* = 7.5

Hz, 2H), 7.06-7.11 (m, 3H), 7.15-7.19 (m, 2H); ^{13}C NMR (125 MHz, $CDCl_3$) δ 14.9, 31.1, 33.1, 47.7, 49.9, 126.1, 128.3, 128.4, 141.2, 172.1.

***N*-(*tert*-Butyl)-3-phenylpropanamide (1e).** ^{1}H NMR (300 MHz, $CDCl_3$) δ 1.29 (s, 9H), 2.38 (t, *J* = 7.2 Hz, 2H), 2.92 (t, *J* = 7.8 Hz, 2H), 5.53 (br, 1H), 7.15-7.21 (m, 3H), 7.23-7.27 (m, 2H); ^{13}C NMR (75 MHz, $CDCl_3$) δ 28.7, 31.9, 39.3, 51.0, 126.1, 128.3, 128.5, 141.1, 171.5.

***N*,*N*-Diethyladamantane-1-carboxamide (1f).** ^{1}H NMR (500 MHz, $CDCl_3$) δ 1.06 (t, *J* = 7.0 Hz, 6H), 1.61-1.68 (m, 6H), 1.90-1.94 (m, 6H), 1.94-1.98 (m, 3H), 3.27-3.43 (m, 4H); ^{13}C NMR (125 MHz, $CDCl_3$) δ 13.7, 28.6, 36.7, 39.1, 41.7, 41.8, 176.0.

1-(2-Methylaziridin-1-yl)-3-phenylpropan-1-one (1g). ^{1}H NMR (500 MHz, $CDCl_3$) δ 1.18 (d, *J* = 5.0 Hz, 3H), 1.79 (d, *J* = 3.0 Hz, 1H), 2.13 (d, *J* = 6.0 Hz, 1H), 2.22-2.27 (m, 1H), 2.63 (td, *J* = 2.0, 7.5 Hz, 2H), 2.89 (t, *J* = 7.5 Hz, 2H), 7.10-7.14 (m, 3H), 7.18-7.22 (m, 2H); ^{13}C NMR (125 MHz, $CDCl_3$) δ 17.7, 31.2, 31.3, 32.7, 38.9, 126.3, 128.4, 128.5, 141.1, 184.9.

***N*-Butyl-3-phenylpropanamide (2a).** ^{1}H NMR (500 MHz, $CDCl_3$) δ 0.80 (t, *J* = 7.0 Hz, 3H), 1.13-1.21 (m, 2H), 1.29-1.35 (m, 2H), 2.39 (t, *J* = 7.5 Hz, 2H), 2.87 (t, *J* = 8.5 Hz, 2H), 3.09-3.13 (m, 2H), 5.79 (br, 1H), 7.09-7.13 (m, 3H), 7.16-7.21 (m, 2H); ^{13}C NMR (125 MHz, $CDCl_3$) δ 13.7, 20.0, 31.6, 31.9, 38.4, 39.3, 126.2, 128.4, 128.5, 140.9, 172.4.

***N*-Butyldecanamide (2b).** ^{1}H NMR (500 MHz, $CDCl_3$) δ 0.80 (t, *J* = 7.0 Hz, 3H), 0.84 (t, *J* = 7.0 Hz, 3H), 1.14-1.31 (m, 14H), 1.37-1.44 (m, 2H), 1.51-1.58 (m, 2H), 2.10 (t, *J* = 7.5 Hz, 2H), 3.13-3.17 (m, 2H), 6.40 (br, 1H); ^{13}C NMR (125 MHz, $CDCl_3$) δ 13.7, 14.0, 20.1, 22.6, 25.9, 29.2, 29.3, 29.4, 29.4, 31.7, 31.8, 36.7, 39.1, 173.4.

***N*-Butyladamantane-1-carboxamide (2c).** ^{1}H NMR (300 MHz, $CDCl_3$) δ 0.77 (t, *J* = 7.5 Hz, 3H), 1.15-1.27 (m, 2H), 1.31-1.41 (m, 2H), 1.53-1.66 (m, 6H), 1.71-1.76 (m, 6H), 1.87-1.94 (m, 3H), 3.07-3.14 (m, 2H), 5.80 (br, 1H); ^{13}C NMR (75 MHz, $CDCl_3$) δ 13.7, 20.0, 28.1, 31.7, 36.5, 38.9, 39.2, 40.4, 177.7.

3-Phenyl-*N*-propylpropanamide (2d). ^{1}H NMR (500 MHz, $CDCl_3$) δ 0.72 (t, *J* = 7.5 Hz, 3H), 1.30-1.37 (m, 2H), 2.36 (t, *J* = 7.5 Hz, 2H), 2.81 (t, *J* = 7.5 Hz, 2H), 3.01 (q, *J* = 7.0 Hz, 2H),

6.60 (br, 1H), 7.02-7.06 (m, 3H), 7.09-7.13 (m, 2H); ^{13}C NMR (125 MHz, $CDCl_3$) δ 11.4, 22.8, 31.9, 38.2, 41.3, 126.1, 128.3, 128.4, 141.0, 172.5.

3-Phenylpropanamide (2e). ^{1}H NMR (500 MHz, $CDCl_3$) δ 2.40 (t, *J* = 7.5 Hz, 2H), 2.84 (t, *J* = 7.5 Hz, 2H), 5.76 (br, 1H), 6.26 (br, 1H), 7.08-7.12 (m, 3H), 7.16-7.20 (m, 2H); ^{13}C NMR (125 MHz, $CDCl_3$) δ 31.4, 37.5, 126.3, 128.3, 128.6, 140.7, 175.5.

***N*-Ethyladamantane-1-carboxamide (2f).** ^{1}H NMR (300 MHz, $CDCl_3$) δ 1.05 (t, *J* = 7.2 Hz, 3H), 1.58-1.70 (m, 6H), 1.74-1.81 (m, 6H), 1.91-1.99 (m, 3H), 3.15-3.24 (m, 2H), 5.81 (br, 1H); ^{13}C NMR (75 MHz, $CDCl_3$) δ 14.9, 28.1, 34.1, 36.5, 39.2, 40.4, 177.8.

***N*-Isopropyl-3-phenylpropanamide (2g).** ^{1}H NMR (500 MHz, $CDCl_3$) δ 0.96 (d, *J* = 7.0 Hz, 6H), 2.33 (t, *J* = 7.5 Hz, 2H), 2.81 (t, *J* = 7.5 Hz, 2H), 3.87-3.96 (m, 1H), 6.14 (d, *J* = 7.0 Hz, 1H), 7.03-7.07 (m, 3H), 7.11-7.15 (m, 2H); ^{13}C NMR (125 MHz, $CDCl_3$) δ 22.6, 31.9, 38.4, 41.2, 126.1, 128.3, 128.4, 141.0, 171.4.

***N*-Methyl-3-phenylpropanamide (1h-SI).** ^{1}H NMR (300 MHz, $CDCl_3$) δ 2.37 (t, *J* = 7.5 Hz, 2H), 2.63 (d, *J* = 5.1 Hz, 3H), 2.84 (t, *J* = 7.5 Hz, 2H), 6.33 (br, 1H), 7.04-7.11 (m, 3H), 7.13-7.19 (m, 2H); ^{13}C NMR (75 MHz, $CDCl_3$) δ 26.3, 31.8, 38.2, 126.2, 128.3, 128.5, 141.0, 173.2.

***N*-Phenyl-3-phenylpropanamide (1i-SI).** ^{1}H NMR (500 MHz, $CDCl_3$) δ 2.54 (t, *J* = 7.0 Hz, 2H), 2.93 (t, *J* = 7.5 Hz, 2H), 6.99 (t, *J* = 7.0 Hz, 1H), 7.09-7.13 (m, 3H), 7.15-7.21 (m, 4H), 7.35 (d, *J* = 8.0 Hz, 2H), 7.45 (br, 1H); ^{13}C NMR (125 MHz, $CDCl_3$) δ 31.6, 39.4, 120.1, 124.4, 126.4, 128.4, 128.7, 130.0, 137.8, 140.7, 170.8.

Reduction of Esters and Carboxylic Acids

According to the general procedure, an ester or carboxylic acid as indicated in Table SI-4 (typically, 0.05 mmol) was reacted with thulium(II) iodide (0.30 mmol) and methanol (0.20 mL, 100 equiv) or water (0.14 mL, 150 equiv) in THF (typically, 1.0 mL) at rt until decolorization to transparent indicative of the formation of Tm^{III} species had occurred. All compounds have been previously reported.

Table SI-4. Reduction of Esters and Carboxylic Acids with TmI_2–ROH at 23 °C.

entry	ester/acid	product	ROH	ROH (equiv)	time[a] (min)	conv.[b] (%)	yield[b] (%)
1	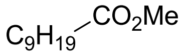	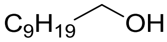	-	-	2-3	7	<10
2	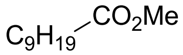	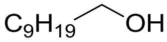	H_2O	150	2-3	>95	83[c]
3	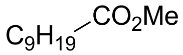	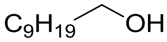	MeOH	100	2-3	>95	99
4	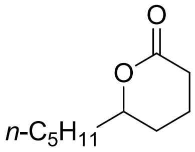	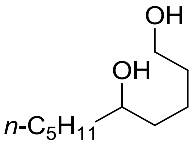	H_2O	150	2-3	>95	88
5	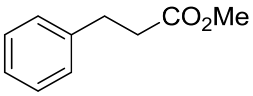	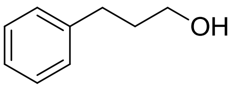	MeOH	100	2-3	>95	96
6[d]	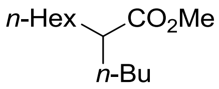	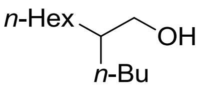	MeOH	100	2-3	63	63
7[d]	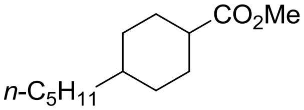	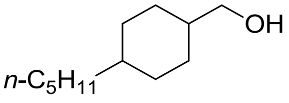	MeOH	100	2-3	>95	94
8	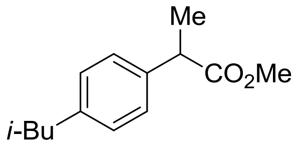	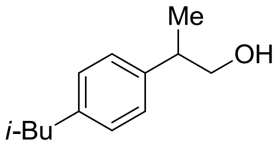	MeOH	100	2-3	86	85
9	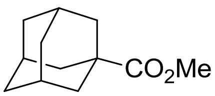	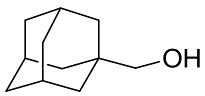	MeOH	100	2-3	59	58
10	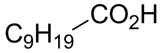	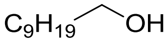	MeOH	100	2-3	<5	<5[e]

All reactions carried out using standard Schlenk techniques. Conditions: under argon, to 6 equiv of TmI_2, substrate (typically, 0.050 mmol) in THF (typically, 1 mL) was added, followed by H_2O or MeOH (150 or 100 equiv). Conversion = (100-SM). [a]Time elapsed until color change from characteristic Tm(II) to Tm(III). [b]Determined by ^{1}H NMR and/or GC-MS. [c]61:39 ratio of 1-decanol/1-decanal. [d]8 equiv of TmI_2 was used. [e]Starting material recovered in >95% yield.

Decan-1-ol (entry 1). ^{1}H NMR (500 MHz, $CDCl_3$) δ 0.81 (t, *J* = 6.9 Hz, 3H), 1.15-1.33 (m, 15H), 1.47-1.52 (m, 2H), 3.57 (t, *J* = 5.8 Hz, 2H); ^{13}C NMR (125 MHz, $CDCl_3$) δ 14.1, 22.7, 25.7, 29.3, 29.4, 29.6, 29.6, 31.9, 32.8, 63.1.

Decane-1,5-diol (entry 4). ^{1}H NMR (500 MHz, $CDCl_3$) δ 0.82 (t, *J* = 6.9 Hz, 3H), 1.19-1.28 (m, 5H), 1.32-1.58 (m, 7H), 1.48-1.58 (m, 2H), 1.61 (br, 2H), 3.51-3.56 (m, 1H), 3.59 (t, *J* = 6.0 Hz, 2H); ^{13}C NMR (125 MHz, $CDCl_3$) δ 14.0, 21.8, 22.6, 25.3, 31.9, 32.6, 37.0, 37.5, 62.7, 71.9.

3-Phenylpropan-1-ol (entry 5). ^{1}H NMR (500 MHz, $CDCl_3$) δ 1.27 (br, 1H), 1.80-1.86 (m, 2H), 2.64 (t, *J* = 7.5 Hz, 2H), 3.61 (t, *J* = 6.5 Hz, 2H), 7.10-7.24 (m, 5H); ^{13}C NMR (125 MHz, $CDCl_3$) δ 32.1, 34.3, 62.3, 125.9, 128.4, 128.5, 141.8.

2-Butyloctan-1-ol (entry 6). ^{1}H NMR (500 MHz, $CDCl_3$) δ 0.79-0.85 (m, 6H), 1.09-1.14 (br, 1H), 1.16-1.29 (m, 16H), 1.35-1.42 (m, 1H), 3.47 (t, *J* = 5.4 Hz, 2H); ^{13}C NMR (100 MHz, $CDCl_3$) δ 14.1, 22.7, 23.1, 26.9, 29.1, 29.8, 30.6, 30.9, 31.9, 40.5, 65.8.

***trans*-4-(Pentylcyclohexyl)methanol (entry 7).** ^{1}H NMR (300 MHz, $CDCl_3$) δ 0.75-0.91 (m, 7H), 1.02-1.27 (m, 10H), 1.38 (br, 1H), 1.71 (d, *J* = 8.7 Hz, 4H), 3.37 (d, *J* = 6.4 Hz, 2H); ^{13}C NMR (75 MHz, $CDCl_3$) δ 14.1, 22.7, 26.6, 29.5, 32.2, 32.7, 37.4, 37.8, 40.7, 68.8.

2-(4-Isobutylphenyl)propan-1-ol (entry 8). ^{1}H NMR (500 MHz, $CDCl_3$) δ 0.83 (d, *J* = 7.0 Hz, 6H), 1.19 (d, *J* = 7.0 Hz, 3H), 1.54 (br, 1H), 1.73-1.82 (m, 1H), 2.38 (d, *J* = 7.0 Hz, 2H), 2.81-2.89 (m, 1H), 3.61 (d, *J* = 6.5 Hz, 2H), 7.02-7.09 (m, 4H); ^{13}C NMR (125 MHz, $CDCl_3$) δ 17.6, 22.4, 30.3, 42.0, 45.0, 68.8, 127.2, 129.4, 140.1, 140.7.

1-Adamantanemethanol (entry 9). ^{1}H NMR (500 MHz, $CDCl_3$) δ 1.23 (br, 1H), 1.44 (m, 6H), 1.57 (m, 1H), 1.59 (m, 2H), 1.65 (m, 2H), 1.68 (m, 1H), 1.92 (m, 3H), 3.13 (s, 2H); ^{13}C NMR (125 MHz, $CDCl_3$) δ 28.2, 34.5, 37.2, 39.0, 73.9.

Mechanistic Studies

Determination of the Redox Potential of TmI_2–MeOH.[9] According to the general procedure, an aromatic hydrocarbon as indicated in Table SI-5 (0.05 mmol) was reacted with thulium(II) iodide (0.15 mmol) and methanol (0.20 mL, 100 equiv) in THF (1.0 mL) at rt for 2-3 min. A small portion of the reaction mixture (200-500 μL) was transferred to a GC vial and analyzed directly by GC and GC-MS to obtain the product distribution by comparison with authentic samples. In case of non-volatile products, ^{1}H NMR analysis was used to obtain the product distribution after the work-up as described in the general methods.

Detailed Investigation of the Role of Proton Donors

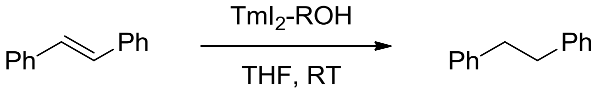

According to the general procedure, *trans*-stilbene (0.05 mmol) was reacted with thulium(II) iodide (0.15 mmol) and a proton donor as indicated in Table SI-6 in THF at rt for 2-3 min unless noted otherwise. The product distribution was obtained by ^{1}H NMR (500 MHz) and GC-MS analysis after work-up as described in the general methods. **Bibenzyl.** ^{1}H NMR (300 MHz, $CDCl_3$) δ 3.10 (s, 4H), 7.33-7.40 (m, 6H), 7.42-7.49 (m, 4H); ^{13}C NMR (75 MHz, $CDCl_3$) δ 38.1, 126.1, 128.5, 128.6, 141.9. ***trans*-Stilbene.** ^{1}H NMR (300 MHz, $CDCl_3$) δ 7.26 (s, 2H), 7.40 (tt, J = 7.5, 1.2 Hz, 2H), 7.50 (m, 4H), 7.65 (m, 4H); ^{13}C NMR (75 MHz, $CDCl_3$) δ 126.7, 127.8, 128.9, 137.5.

Additional Discussion. Following the fact that the reduction of aromatic hydrocarbons is well-established to proceed via an outer-sphere electron transfer[10] and a recent report in which TmI_2 was shown to reduce aromatic hydrocarbons with redox potentials lower than E° = -2.0 V vs. SCE,[11] stilbene (E° = -2.2 V vs. SCE) was selected as a representative substrate for this study (Table SI-6). In agreement with the literature, no reaction was observed when stilbene was subjected to a THF solution of TmI_2 even for extended periods of time (entries 1, 2). Remarkably, however, in the presence of protic additives, MeOH (entry 3) or H_2O (entry 4), but under otherwise identical reaction conditions, efficient reduction of stilbene to bibenzyl occurred. The reaction proceeded even when only approx. 3 equiv of alcohol with respect to TmI_2 were used to form the active Tm(II) complex (entries 5 and 6), while non-coordinating alcohols as well as a tri-component system based proved less effective in promoting the reduction (entries 7 and 8). The latter effect is consistent with studies on the role of proton donors on the reactivity of SmI_2.[10] A tri-component system based on amine-proton donor was also less effective, possibly due to the difficulties in achieving a homogenous solution under these conditions (entry 9).[12] Overall, these experiments demonstrated for the first time that the reactivity of non-classical lanthanide(II) iodides can be significantly modulated by the use of proton donors and suggested that a much lower concentration of alcohols is required to enhance the redox potential of TmI_2 in comparison with SmI_2.[13]

Table SI-5. Determination of Redox Potential of TmI_2 by Reduction of Aromatic Hydrocarbons.

entry	hydrocarbon	$-E_{1/2}{}^{a}$	major product	conversion (%)
1[b]	cyclooctatetraene	1.83	cyclooctadiene	>98
2	anthracene	1.98	9,10-dihydroanthracene	84
3	*trans*-stilbene	2.21	bibenzyl	>98
4	1,4-dipehenylbenzene	2.40	dihydro-1,4-diphenylbenzene	40
5	1,3,5-triphenylbenzne	2.51	dihydrotriphenylbenzene	15
6	naphthalene	2.61	1,4-dihydronaphthalene	1.8
7	styrene	2.65	ethylbenzene	46
8	benzene	3.42	1,4-cyclohexadiene	<2

See, Table SI-1. [a]In volts vs. SCE; $-E_{1/2}$ describes half-reduction potential. Ref. 9. [b]Ref 11.

Table SI-6. Detailed Investigation of the Role of Proton Donor (Reduction of *trans*-Stilbene).

entry	TmI_2 (equiv)[a]	ROH	ROH (equiv)[a]	time[b]	conversion[c] (%)	yield[c] (%)	notes
1	3	-	-	3 min	<5	<5	-
2[d]	3	-	-	1 h	<5	<5	-
3	3	MeOH	100	3 min	>95	96	-
4	3	H_2O	150	3 min	86	81	-
5	3	MeOH	10	3 min	>95	99	-
6	3	H_2O	10	3 min	>95	99	-
7	3	*t*-BuOH	10	3 min	<5	<5	-
8	3	TFE	10	3 min	28	28	-
9	3	Et_3N/H_2O	18/18	<5 s	20	20	-
10	3	MeOD-d_4	100	1.5 min	>95	93	96.5% D^2-purity
11[e]	3	MeOD-d_4/MeOH	100	1.5 min	>95	72	k_H/k_D = 1.13±0.1
12	3	D_2O	100	1 min	>95	94	98.0% D^2-purity
13[e]	3	D_2O/H_2O	100	1 min	>95	91	k_H/k_D = 1.27±0.1

All reactions carried out using standard Schlenk techniques. Conditions: under argon, to 3 equiv of TmI_2, substrate (0.050 mmol) in THF (typically, 1 mL) was added, followed by an alcohol. Conversion = (100-SM). [a]With respect to *trans*-stilbene. [b]Indicates time after which decolorization from deep green (Tm^{II}) to transparent, yellow or milky-white (Tm^{III}) had occurred. [c]Determined by ^{1}H NMR analysis by comparison with authentic samples. [d]Reaction carried out in the dark. [e]1:1 mixture of MeOD-d_4/MeOH or D_2O/H_2O was used.

Determination of Deuterium Incorporation and KIE[14,15]

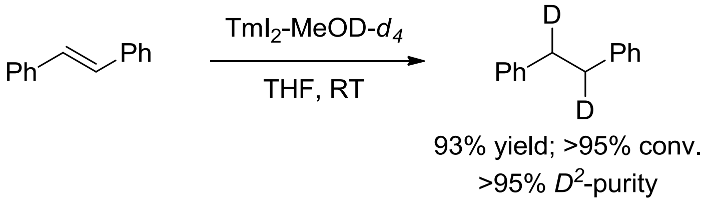

(1)

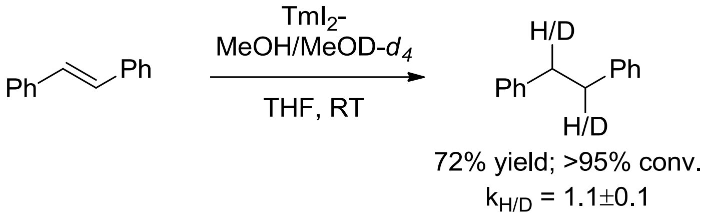

(2)

Eq 1. According to the general procedure, *trans*-stilbene (0.05 mmol) was reacted with thulium(II) iodide (0.15 mmol) and methanol-d_4 (0.20 mL, 100 equiv) in THF (1.0 mL) for 2-3 min at rt to afford 1,2-*D*,*D*-1,2-diphenylethane in 93% yield (96.5% D_2 incorporation).

Eq 2. According to the general procedure, *trans*-stilbene (0.05 mmol) was reacted with thulium(II) iodide (0.15 mmol) and methanol/methanol-d_4 (1:1, 0.20 mL, 100 equiv) in THF (1.0 mL) for 2-3 min at rt to afford 1,2-*D*,*D*-1,2-diphenylethane and bibenzyl (>95% conversion). The amount of each species was determined by ^{1}H NMR (500 MHz, $CDCl_3$). Kinetic isotope effect, k_H/k_D = 1.13±0.1. **1,2-*D*,*D*-1,2-Diphenylethane.** ^{1}H NMR (500 MHz, $CDCl_3$) δ = 2.82 (s, 2 H), 7.10-7.14 (m, 6 H), 7.19-7.23 (m, 4 H); ^{13}C NMR (75 MHz, $CDCl_3$) δ = 37.5 (t, J^1 = 19.0 Hz), 125.9, 128.3, 128.5, 141.8. Deuterium incorporation and kinetic isotope effect using TmI_2–D_2O was determined following the same procedure (see, Table SI-6, entries 12-13 for details).

<u>*Studies on the Origin of Selectivity in Ester Reduction*</u>

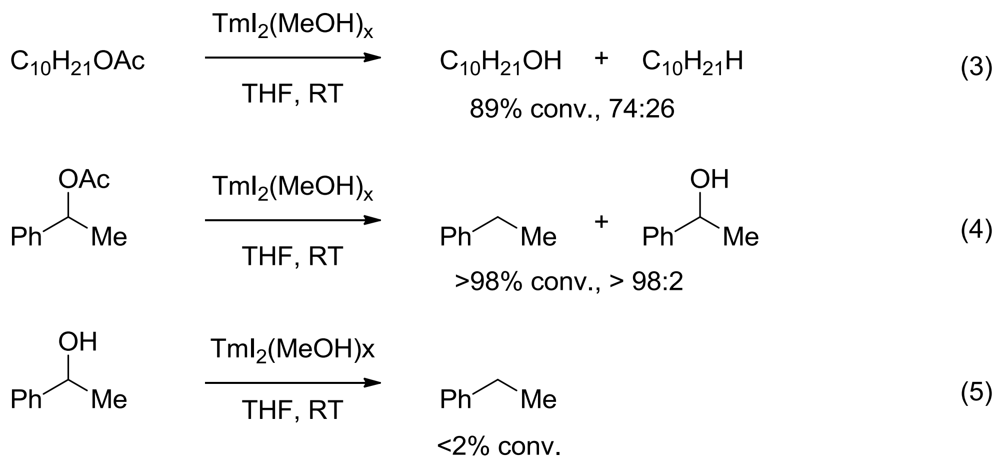

According to the general procedure, decyl acetate, 1-phenylethyl acetate or 1-phenylethanol, as indicated in equations 1-3 (typically, 0.05 mmol), was reacted with thulium(II) iodide (eq 3-4: 6 equiv; eq 5: 3 equiv), and methanol (100 equiv) in THF (typically, 1.0 mL) until decolorization to transparent indicative of formation of Tm^{III} species had occurred. A small portion of the reaction mixture (200-500 μL) was transferred to a GC vial. The product distribution was analyzed directly by GC and GC-MS and comparison with authentic samples. All data represent values corrected for response factors obtained by analyzing known quantities of the starting materials/products. All compounds have been previously reported and are commercially available.

Chart SI-1. Comparison of Reactivity of SmI_2–ROH and TmI_2–ROH.

entry	substrate	SmI_2-ROH[a]	TmI_2-ROH[b]
1	aromatic hydrocarbons	cyclooctatetraene ($E_{1/2}$ = -1.83 V)	naphthalene ($E_{1/2}$ = -2.61 V)
2	lactones, 6-membered	+ (limited scope)	+
3	lactones, 5-membered	-	+
4	lactones, 7-membered	-	+
5	RCOOR'	-	+
6	RCONR'$_2$	-	+
7	ArCH=CHAr	-	+
8	RCOOH	-	-

[a]See, the list of seminal publications below. [b]This work.

List of Seminal References

SmI_2–ROH:

1. The Role of Proton Donors in SmI_2-Mediated Ketone Reduction: New Mechanistic Insights, Chopade, P. R.; Prasad, E.; Flowers, R. A., II, *J. Am. Chem. Soc.*, **2004**, *126*, 44.

2. A Ring Size-Selective Reduction of Lactones Using SmI_2 and H_2O, Duffy, L. A.; Matsubara, H.; Procter, D. J., *J. Am. Chem. Soc.*, **2008**, *130*, 1136.

3. Guidelines for the Use of Proton Donors in SmI_2 Reactions: Reduction of α-Cyanostilbene, Amiel-Levy, M.; Hoz, S., *J. Am. Chem. Soc.*, **2009**, *131*, 8280.

TmI_2:

1. Synthesis and Structure of the First Molecular Thulium(II) Complex: $[TmI_2(MeOCH_2CH_2OMe)_3]$, Bochkarev, M. N.; Fedushkin, I. L.; Fagin, A. A.; Petrovskaya, T. V.; Ziller, J. W.; Broomhall-Dillard, R. N. R.; Evans, W. J., *Angew. Chem. Int. Ed.*, **1997**, *36*, 133.

2. Ketone Coupling with Alkyl Iodides, Bromides, and Chlorides Using Thulium Diiodide: a More Powerful Version of $SmI_2(THF)_x$/HMPA, Evans, W. J.; Allen, N. T., *J. Am. Chem. Soc.*, **2000**, *122*, 2118.

Chart SI-2. Comparison of Reactivity of SmI_2–ROH and TmI_2–ROH: Representative Examples.

entry	substrate	SmI_2–ROH (equiv)	time[a] (h)	conv.[b]	TmI_2–ROH (equiv)	time[a] (min)	conv.[b]
1	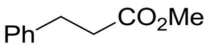	6-800/H_2O	24	<2%	6-100/MeOH	2-3	>95%
2	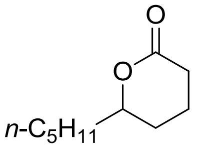	6-800/H_2O	24	88%	6-150/H_2O	2-3	>95%
3	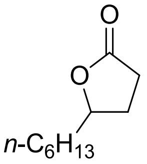	8-800/H_2O	8	<5%	6-150/H_2O	2-3	>95%
4	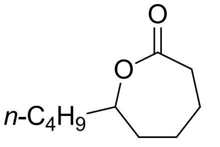	8-800/H_2O	8	<5%	6-150/H_2O	2-3	>95%
5	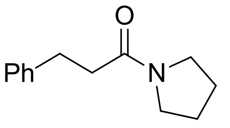	6-200/H_2O	1	<5%	6-200/MeOH	2-3	89%
6	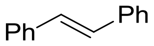	6-10/H_2O	24	<5%	3-10/MeOH	2-3	>95%

All reactions carried out using standard Schlenk techniques. [a]Indicates time after which decolorization of Ln^{II}–ROH from deep green (Tm^{II}) to transparent, yellow or milky-white (Tm^{III}) or from burgundy-red (Sm^{II}) to transparent or yellow (Sm^{III}) had occurred. [b]Determined by GC-MS and/or ^1H NMR and comparison with authentic samples.

References

1. P. Girard, J. L. Namy and H. B. Kagan, *J. Am. Chem. Soc.*, **1980**, *102*, 2693.
2. W. J. Evans and N. T. Allen, *J. Am. Chem. Soc.*, **2000**, *122*, 2118.
3. M. N. Bochkarev and A. A. Fagin, *Chem. Eur. J.*, **1999**, *5*, 2990.
4. E. Prasad and R. A. Flowers, II, *J. Am. Chem. Soc.*, **2005**, *127*, 18093.
5. (a) P. R. Chopade, E. Prasad and R. A. Flowers, II, *J. Am. Chem. Soc.*, **2004**, *126*, 44. (b) M. Amiel-Levy and S. Hoz, *J. Am. Chem. Soc.*, **2009**, *131*, 820. (c) D. V. Sadasivam, J. A. Teprovich, Jr., D. J. Procter and R. A. Flowers, II, *Org. Lett.*, **2010**, *12*, 4140.
6. (a) L. B. Asprey and F. H. Kruse, *Inorg. Nucl. Chem.*, **1960**, *13*, 32. (b) R. G. Bulgakov, S. P. Kuleshov, Z. S. Kinzyabaeva, A. A. Fagin, I. R. Masalimov and M. N. Bochkarev, *Russ. Chem. Bull. Int. Ed.*, **2007**, *56*, 1956.
7. C. Leroy, D. Peeters and C. Wilante, *THEOCHEM*, **1982**, *5*, 217.
8. Y. Lei, A. D. Wrobleski, J. E. Golden, D. R. Powell and J. Aubé, *J. Am. Chem. Soc.*, **2005**, *127*, 4552.
9. W. J. Evans, S. L. Gonzales and W. J. Ziller, *J. Am. Chem. Soc.*, **1994**, *116*, 2600.
10. M. Amiel-Levy, S. Hoz, *J. Am. Chem. Soc.*, **2009**, *131*, 8280.
11. I. L. Fedushkin, M. N. Bochkarev, S. Dechert and H. Schumann, *Chem. Eur. J.*, **2001**, *7*, 3558.
12. A. Dahlén, G. Hilmersson, *Eur. J. Inorg. Chem.*, **2004**, 3393.
13. P. R. Chopade, E. Prasad, R. A. Flowers, II, *J. Am. Chem. Soc.*, **2004**, *126*, 44.
14. (a) M. Szostak, M. Spain and D. J. Procter, *Chem. Commun.*, **2011**, *47*, 10254. (b) D. Parmar, L. A. Duffy, D. V. Sadasivam, H. Matsubara, P. A. Bradley, R. A. Flowers, II and D. J. Procter, *J. Am. Chem. Soc.*, **2009**, *131*, 15467. (c) A. Dáhlen and G. Hilmersson, *Chem. Eur. J.*, **2003**, 9, 1123.
15. E. M. Simmons and J. F. Hartwig, *Angew. Chem. Int. Ed.*, **2012**, *51*, 3066.

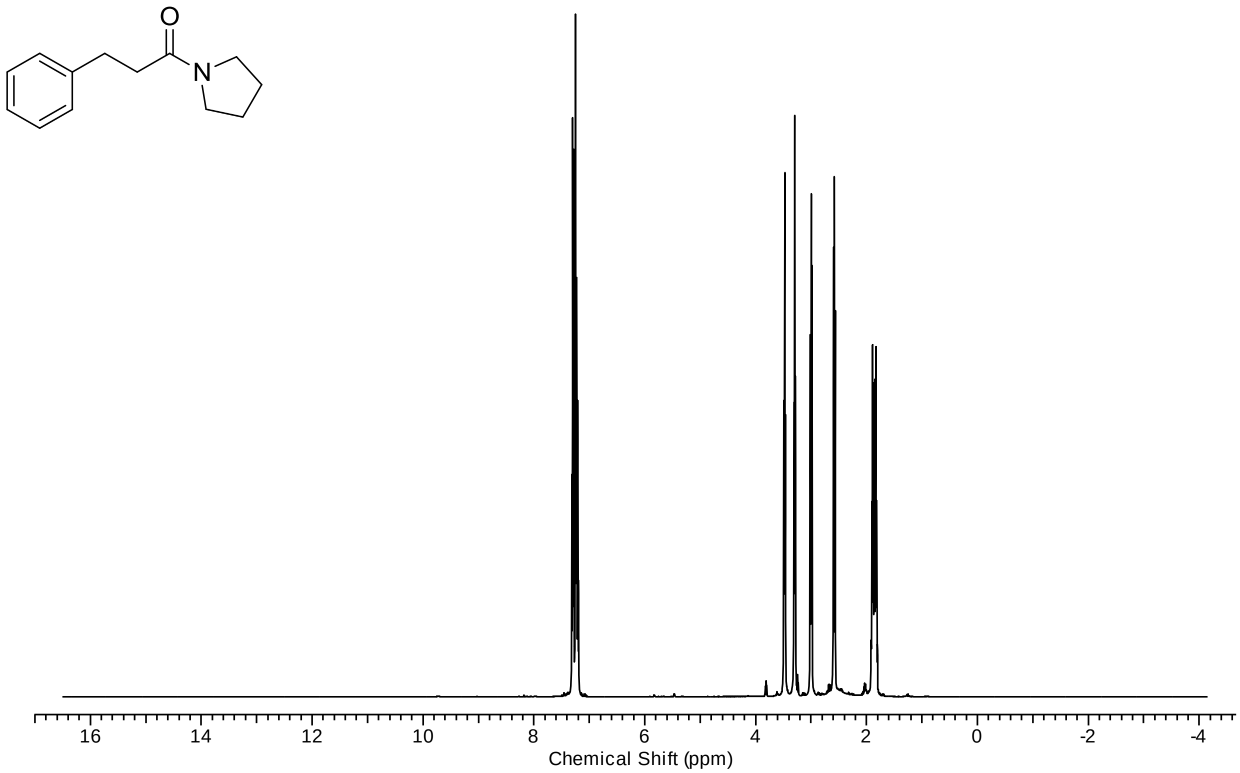
O
N
16 14 12 10 8 6 4 2 0 -2 -4
Chemical Shift (ppm)

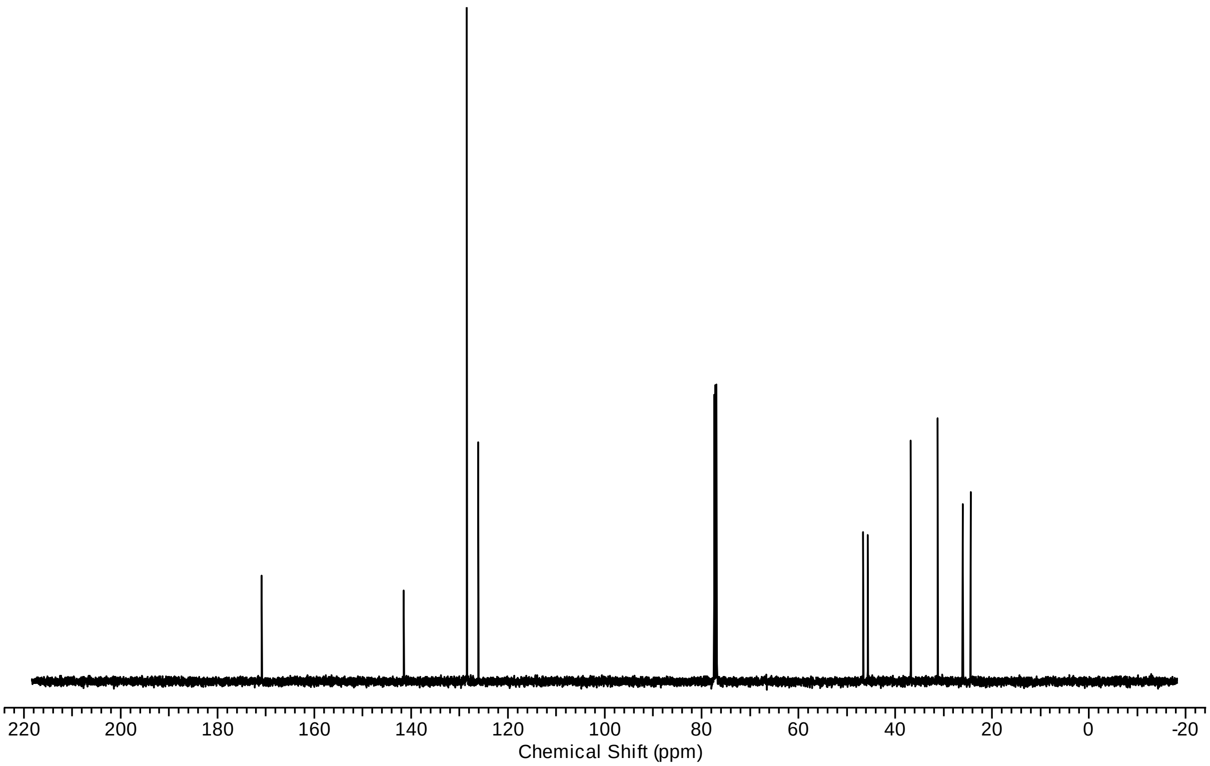
220 200 180 160 140 120 100 80 60 40 20 0 -20
Chemical Shift (ppm)

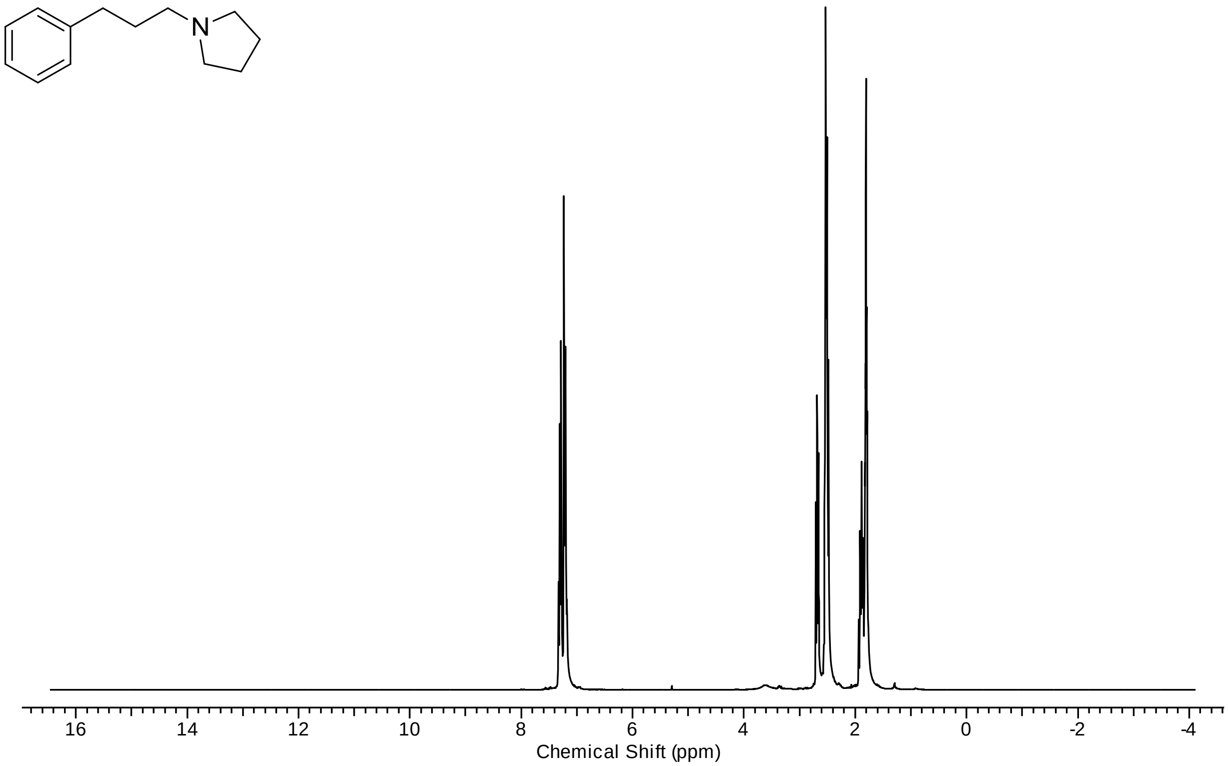
N
16
14
12
10
8
6
4
2
0
-2
-4
Chemical Shift (ppm)

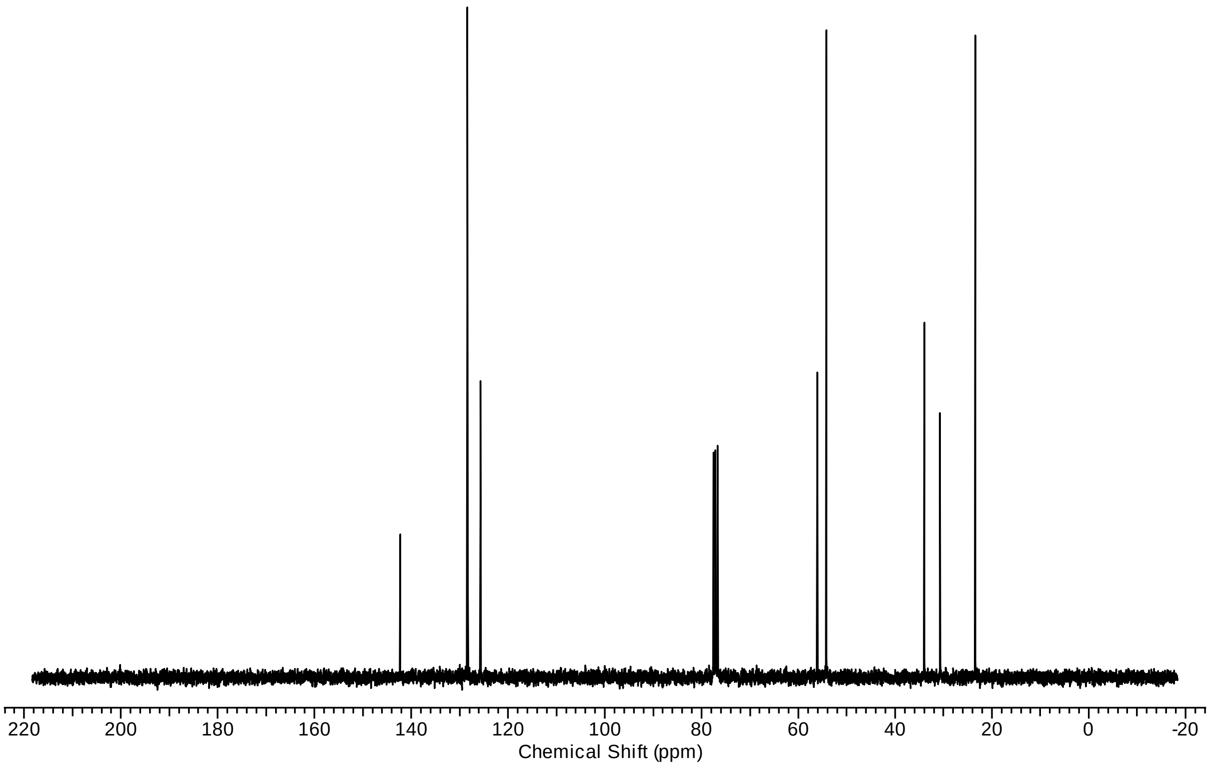
220
200
180
160
140
120
100
80
60
40
20
0
-20
Chemical Shift (ppm)

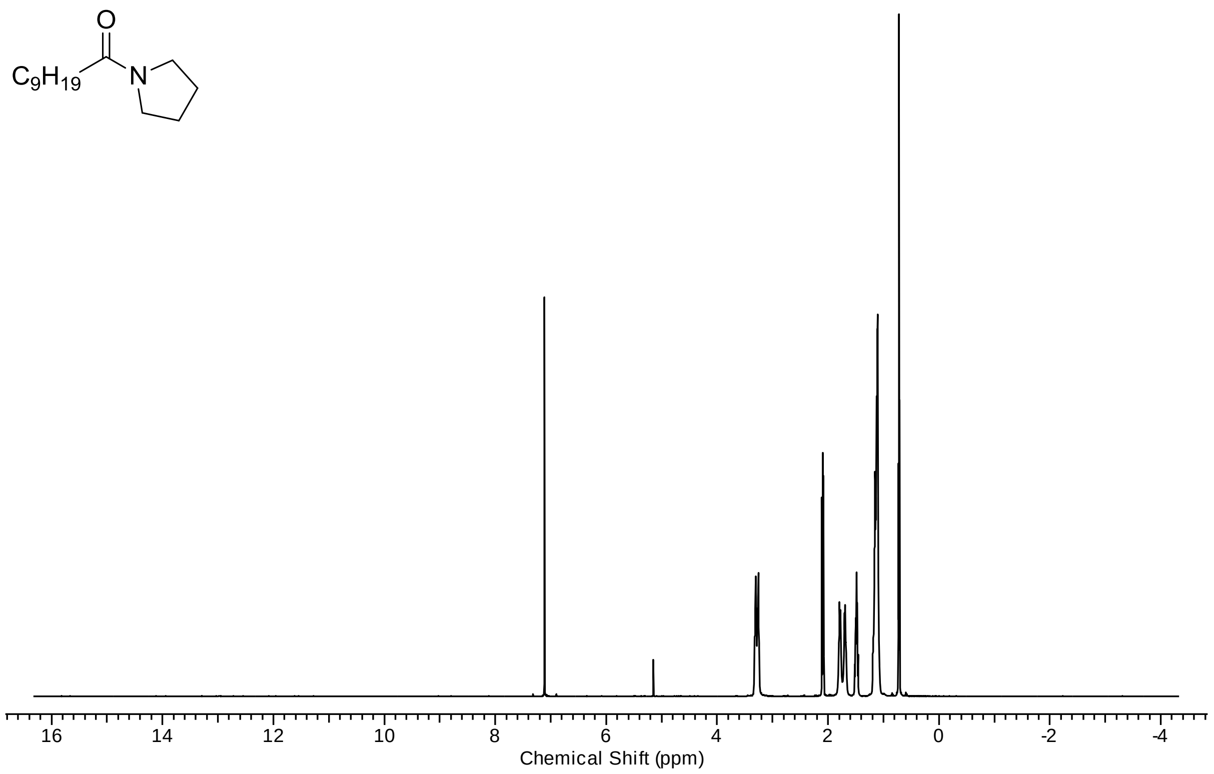
O
C_9H_{19}
N
16
14
12
10
8
6
4
2
0
-2
-4
Chemical Shift (ppm)

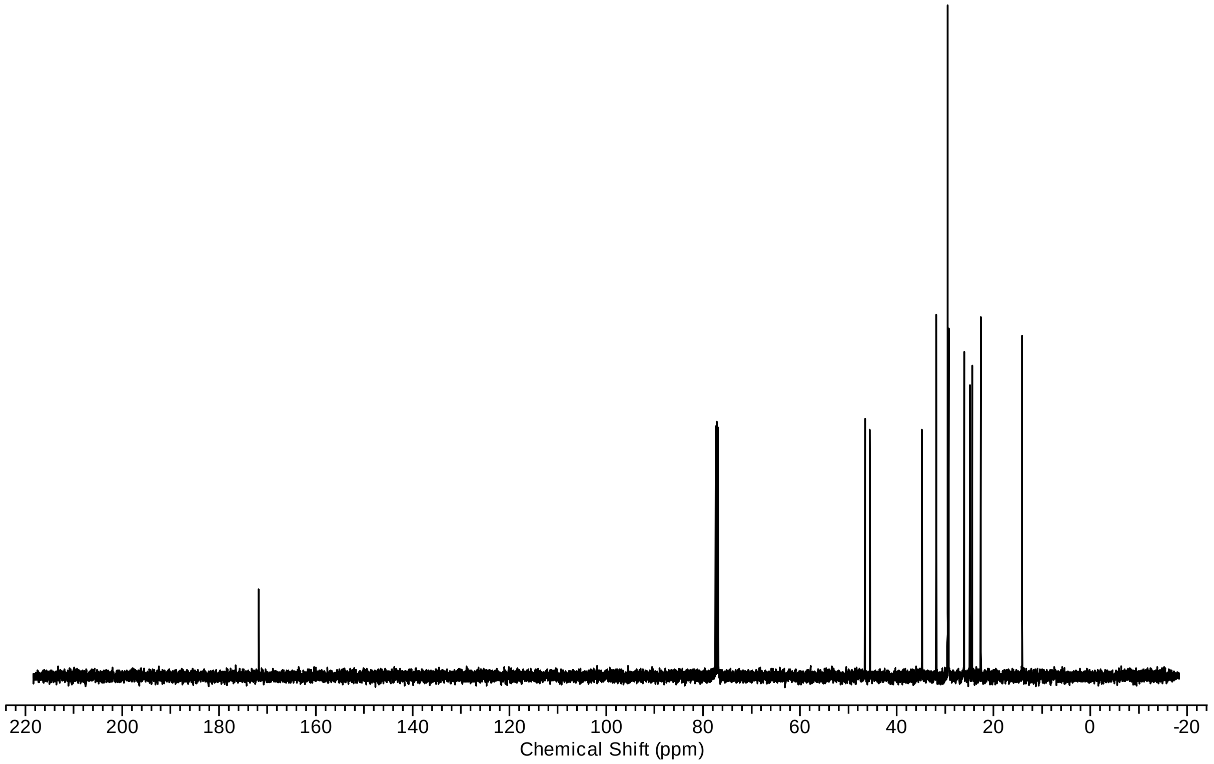
220
200
180
160
140
120
100
80
60
40
20
0
-20
Chemical Shift (ppm)

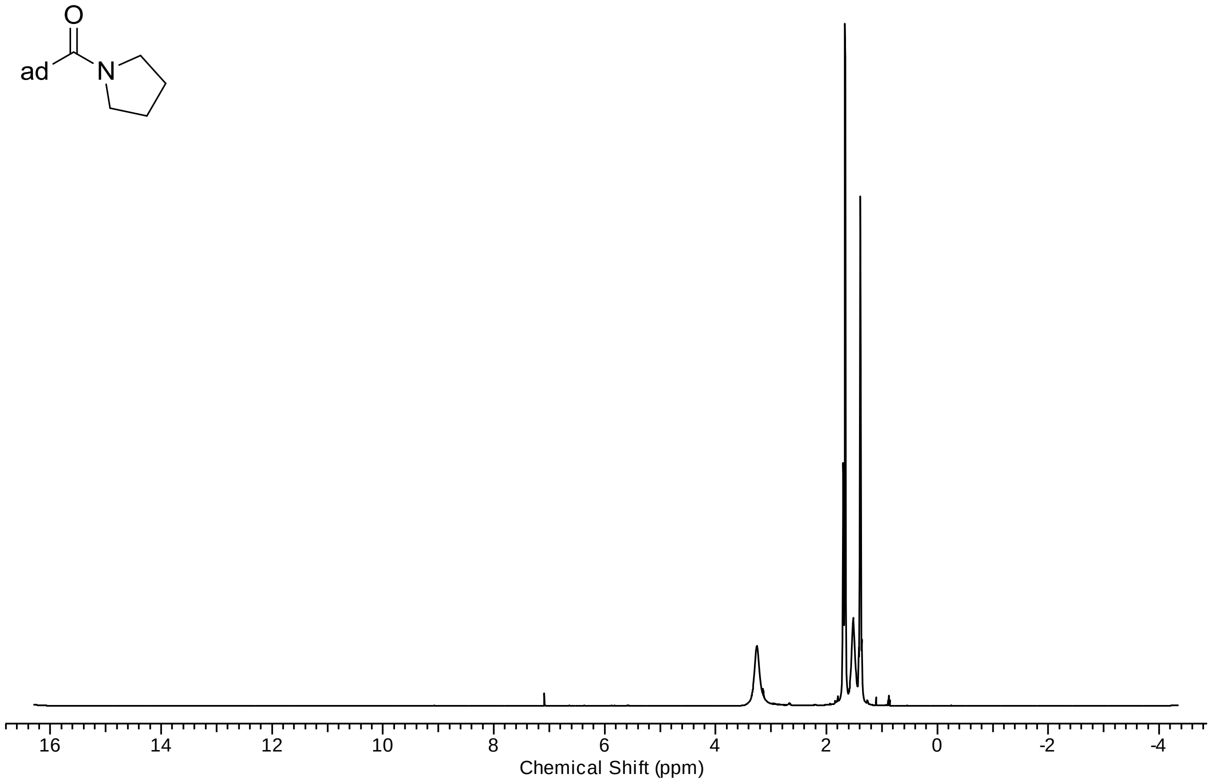
O
ad
N
16 14 12 10 8 6 4 2 0 -2 -4
Chemical Shift (ppm)

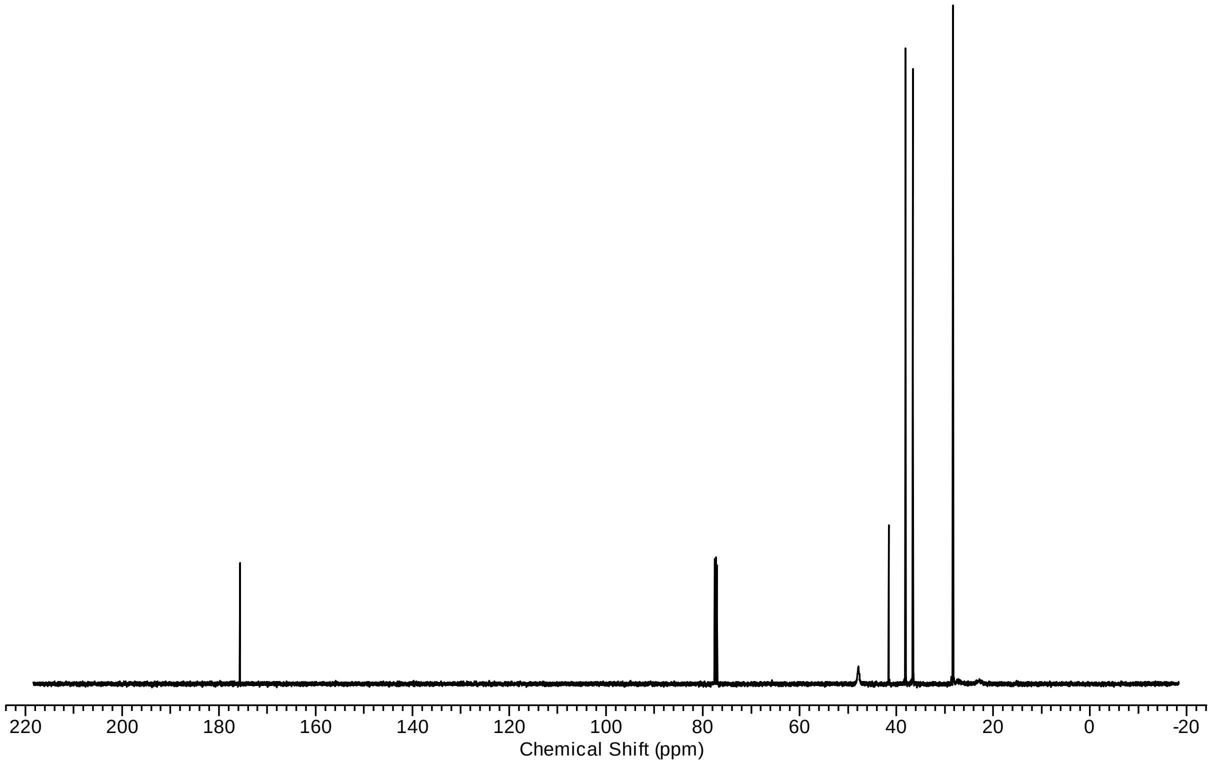
220 200 180 160 140 120 100 80 60 40 20 0 -20
Chemical Shift (ppm)

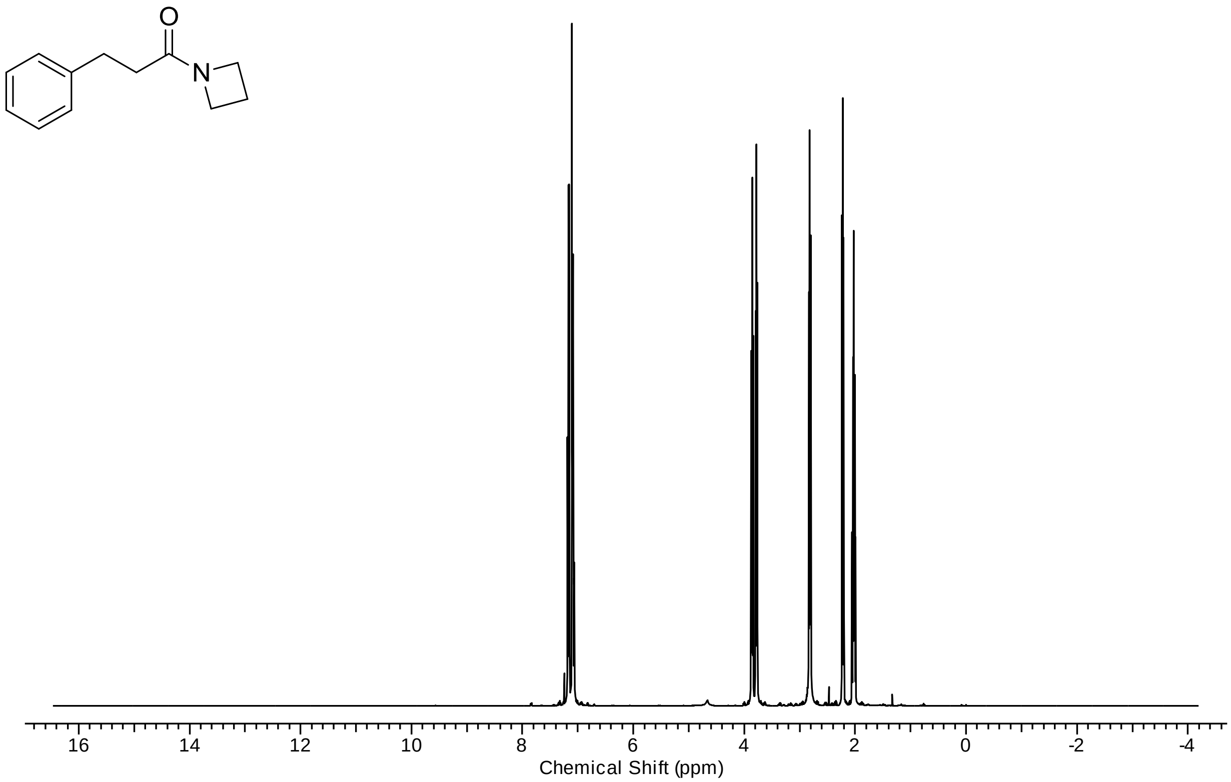
O
N
16
14
12
10
8
6
4
2
0
-2
-4
Chemical Shift (ppm)

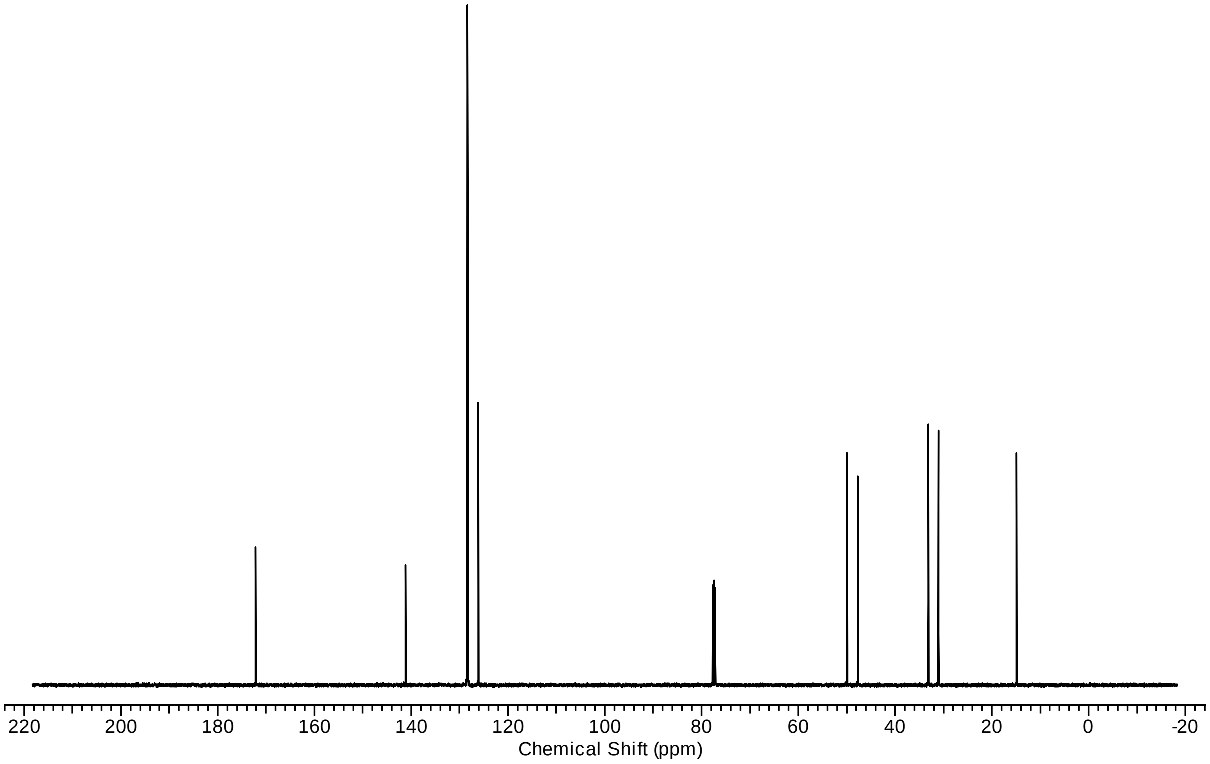
220
200
180
160
140
120
100
80
60
40
20
0
-20
Chemical Shift (ppm)

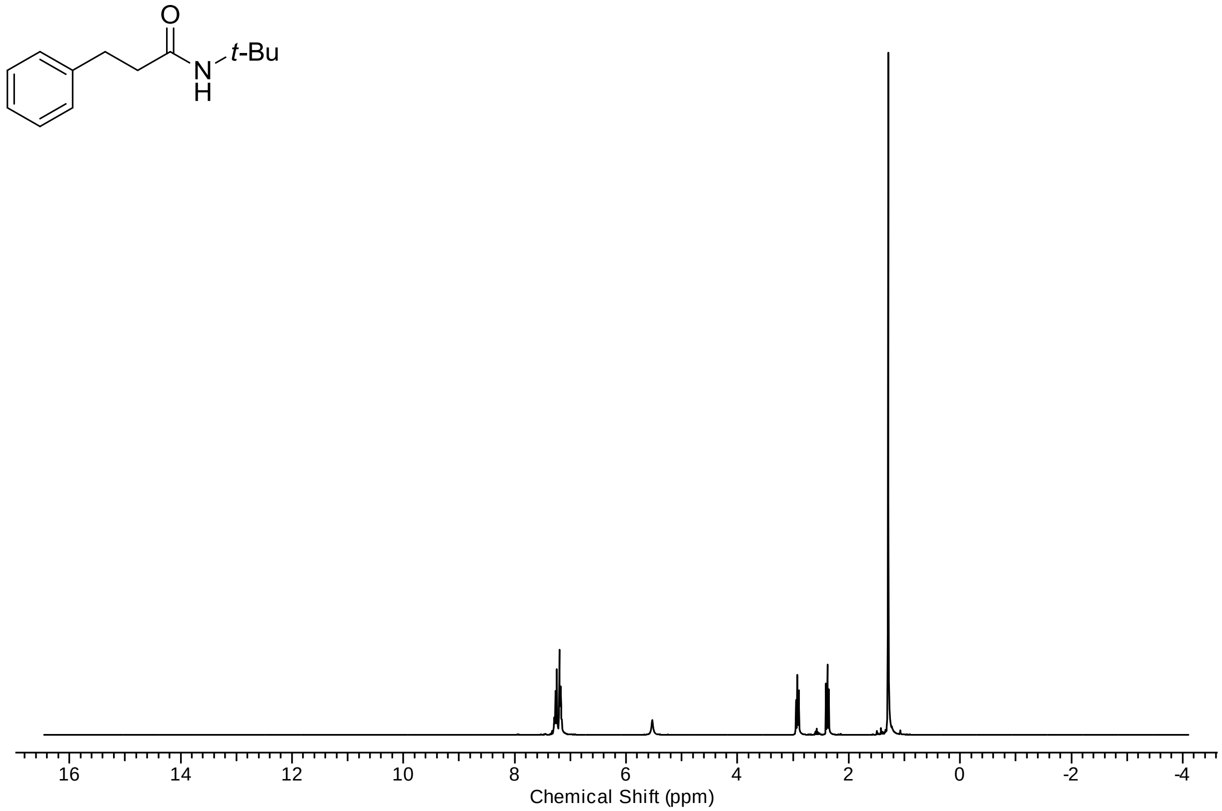
O
N
H
t-Bu
16
14
12
10
8
6
4
2
0
-2
-4
Chemical Shift (ppm)

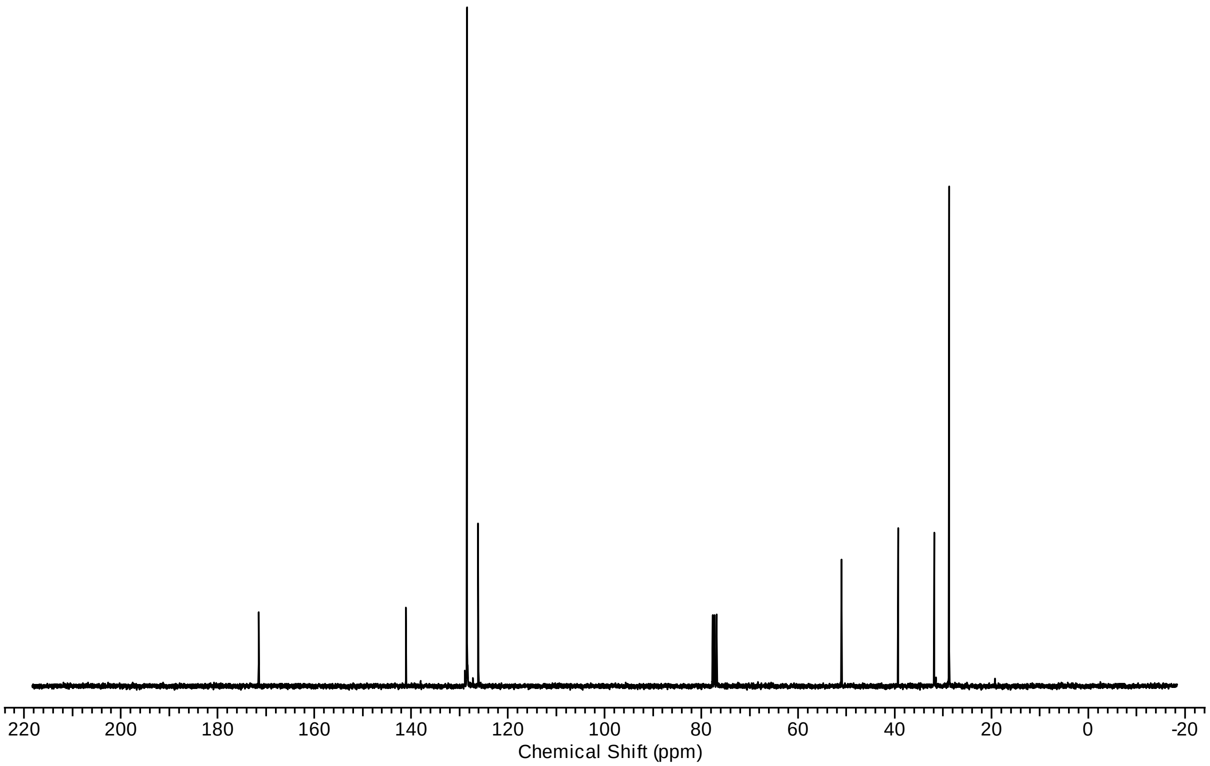
220
200
180
160
140
120
100
80
60
40
20
0
-20
Chemical Shift (ppm)

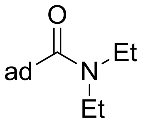
O
ad
N
Et
Et

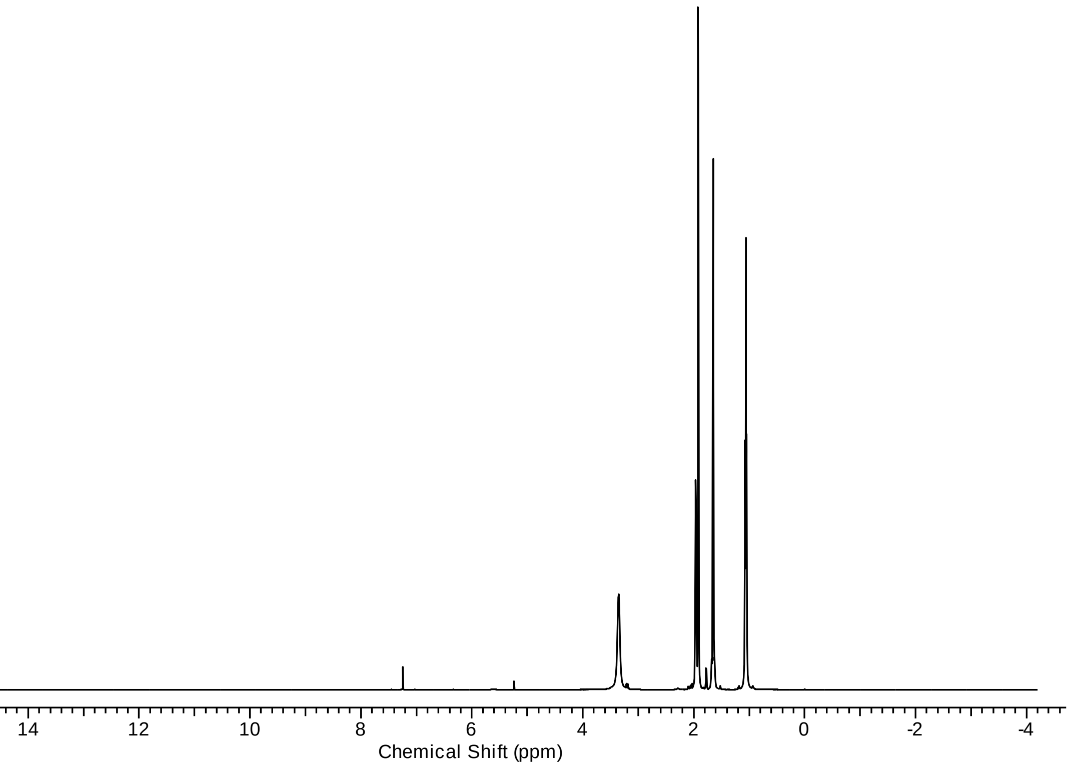
16
14
12
10
8
6
4
2
0
-2
-4
Chemical Shift (ppm)

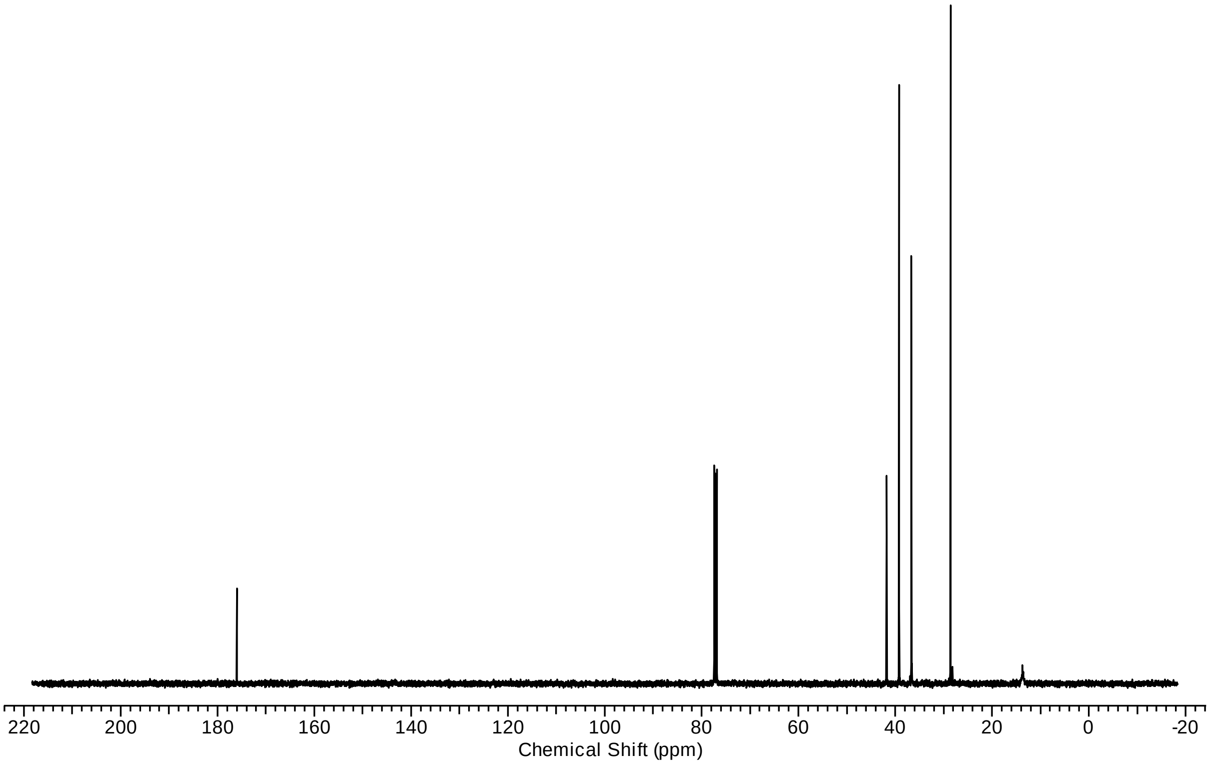
220
200
180
160
140
120
100
80
60
40
20
0
-20
Chemical Shift (ppm)

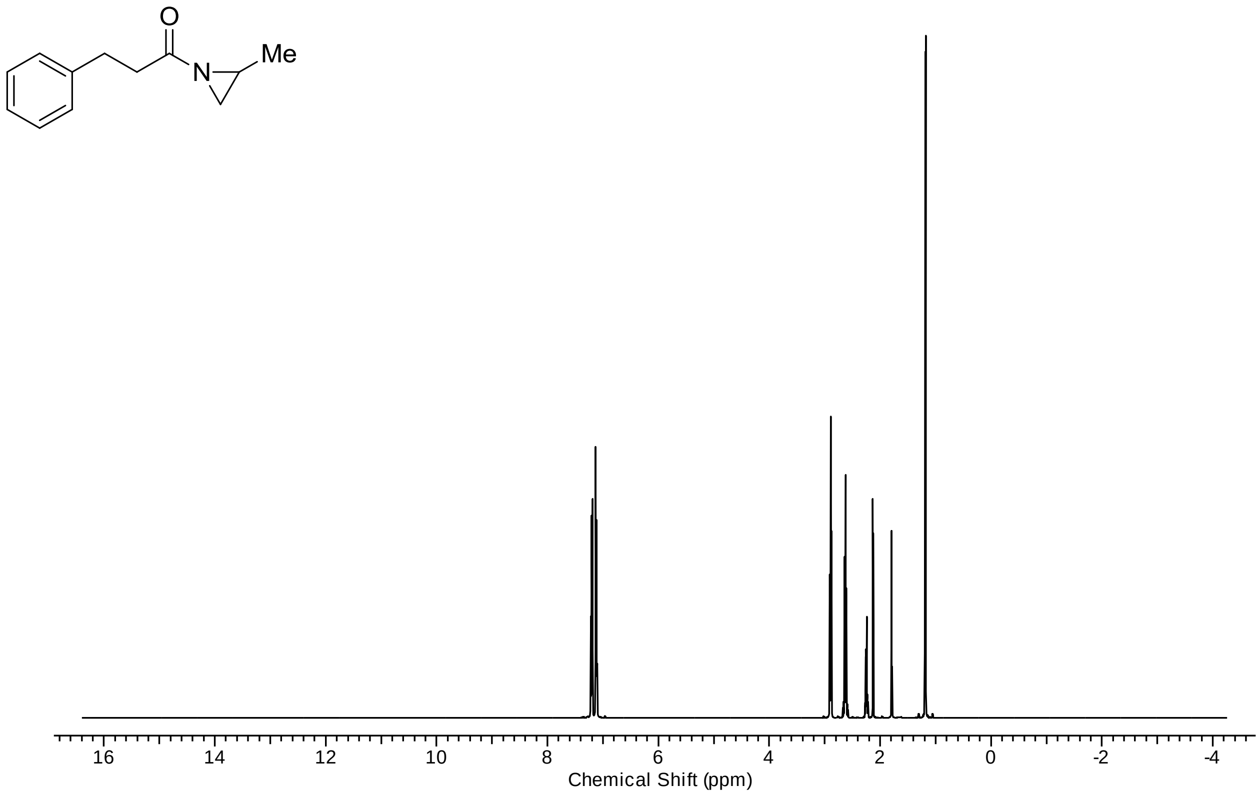
O
N
Me
16 14 12 10 8 6 4 2 0 -2 -4
Chemical Shift (ppm)

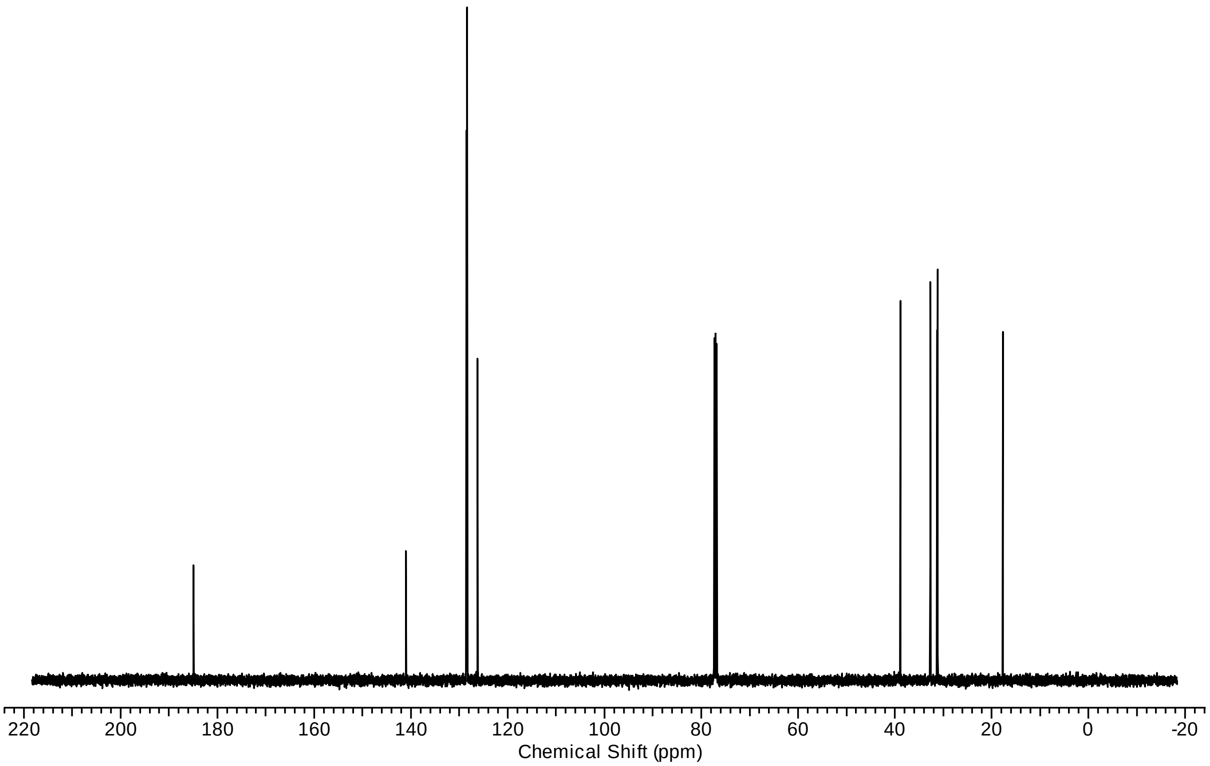
220 200 180 160 140 120 100 80 60 40 20 0 -20
Chemical Shift (ppm)

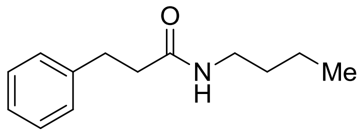
O
N
H
Me

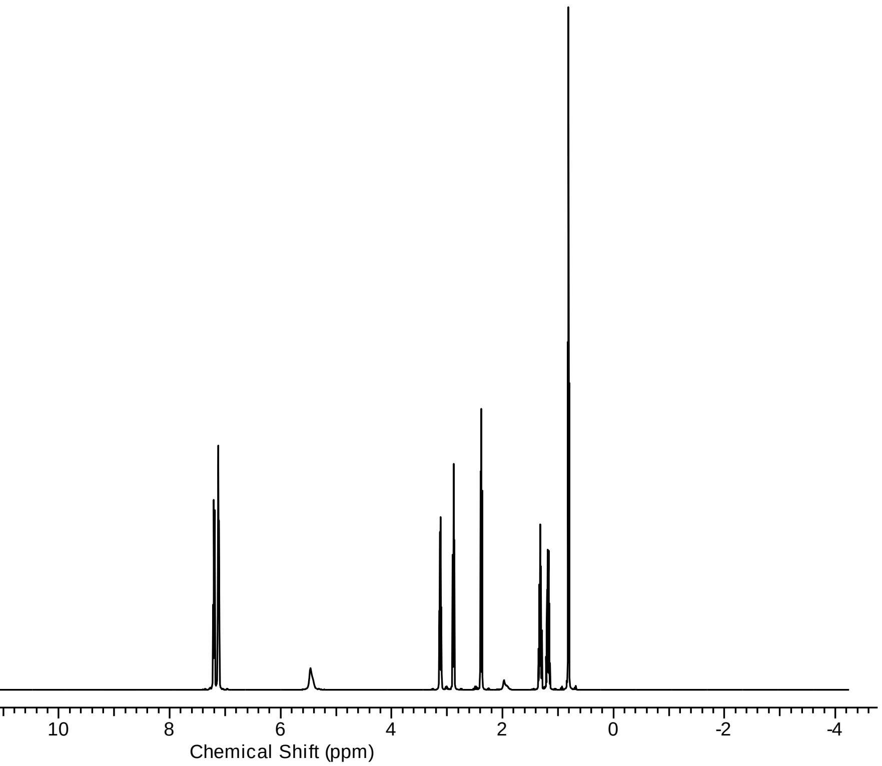
16 14 12 10 8 6 4 2 0 -2 -4
Chemical Shift (ppm)

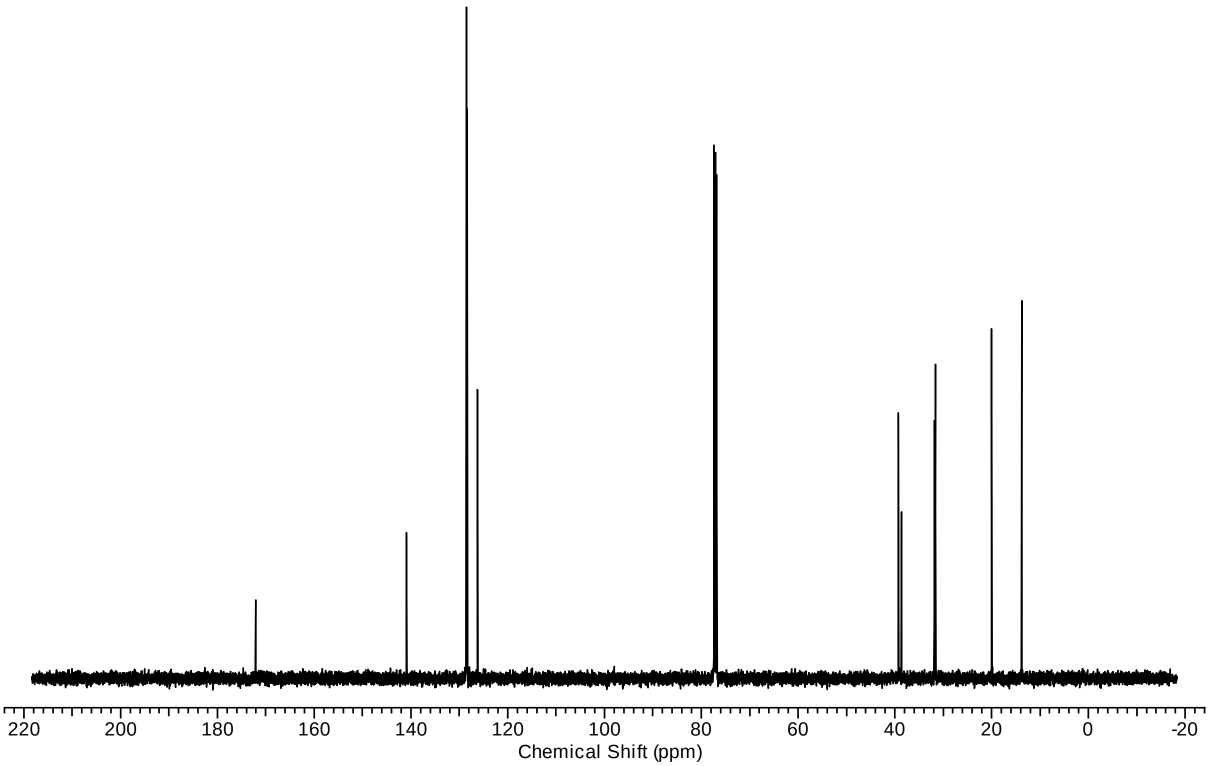
220 200 180 160 140 120 100 80 60 40 20 0 -20
Chemical Shift (ppm)

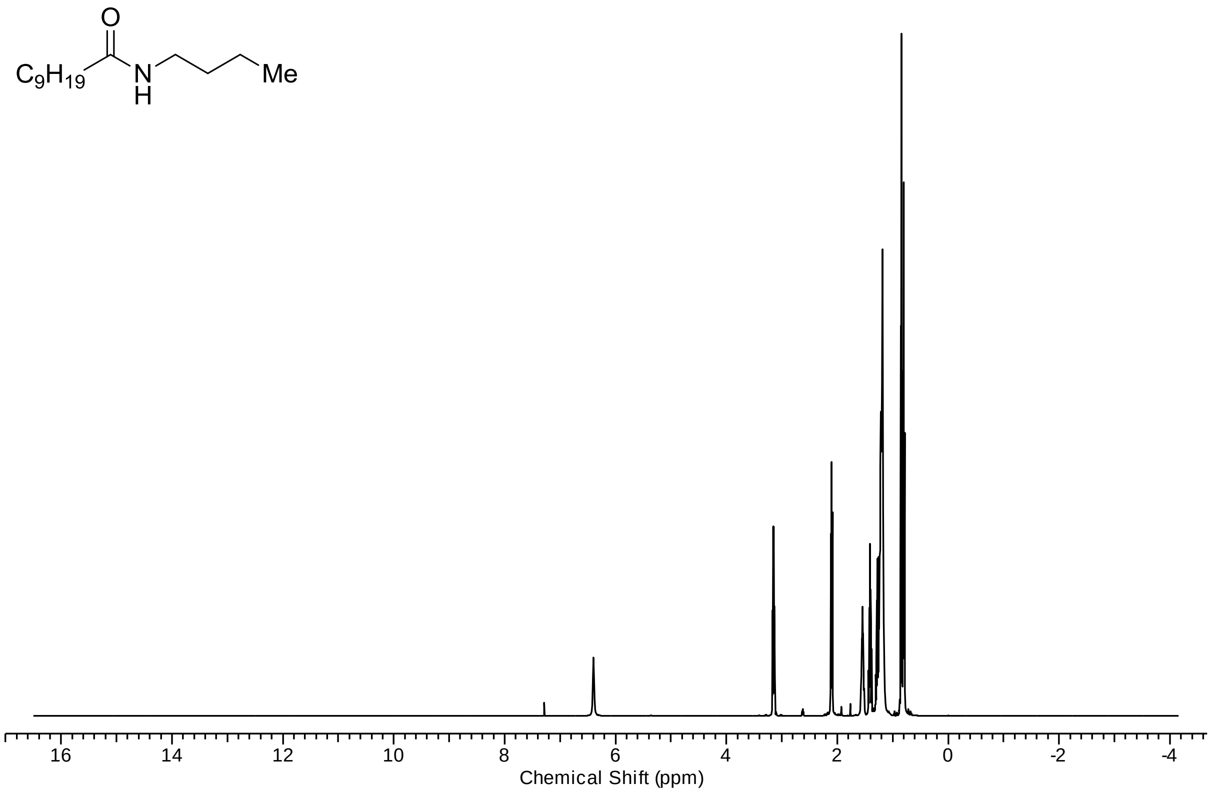
O
C9H19
N
H
Me
16 14 12 10 8 6 4 2 0 -2 -4
Chemical Shift (ppm)

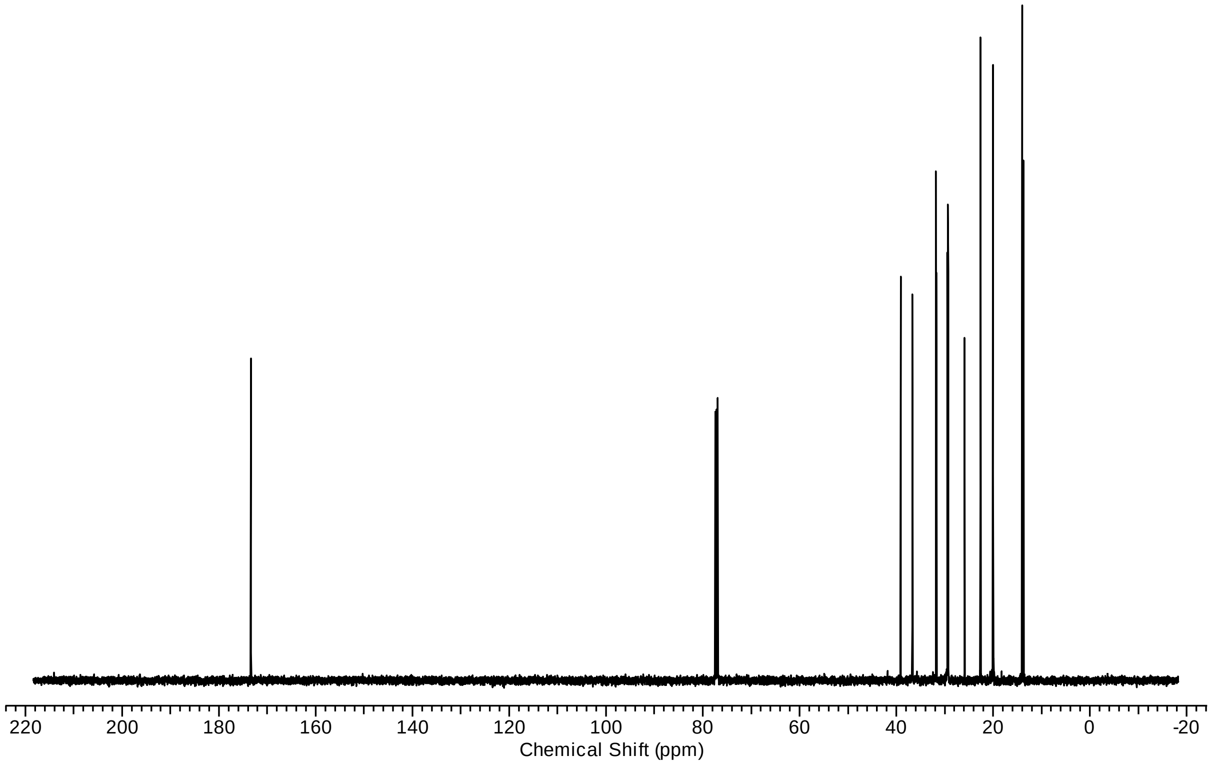
220 200 180 160 140 120 100 80 60 40 20 0 -20
Chemical Shift (ppm)

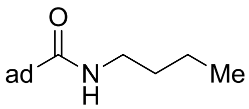
O
ad
N
H
Me

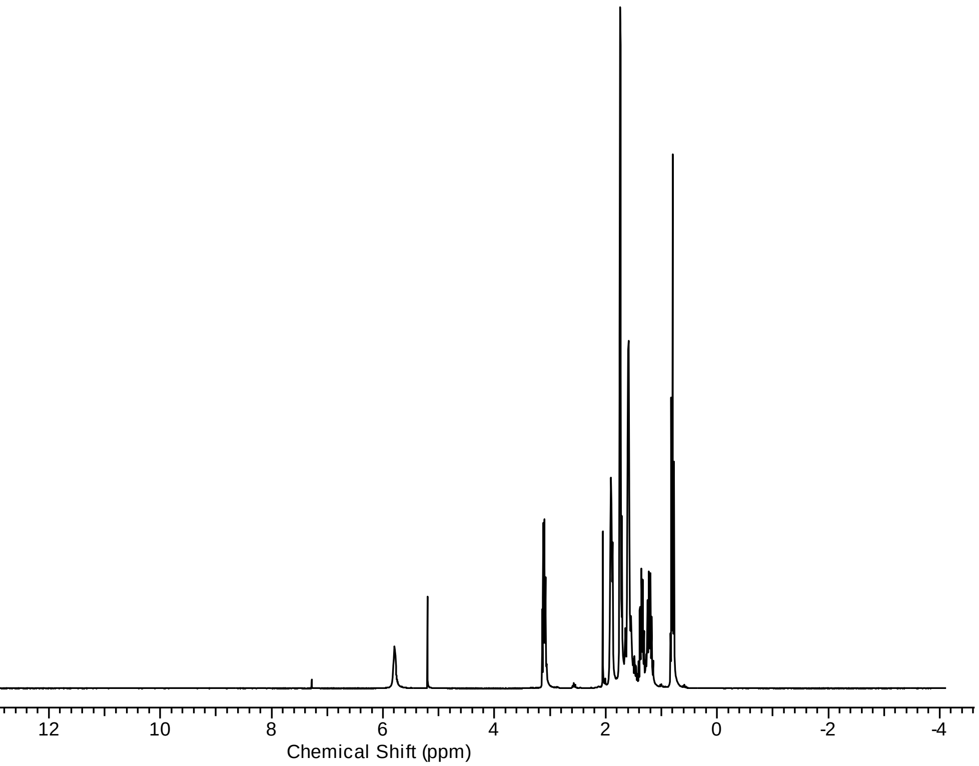
16 14 12 10 8 6 4 2 0 -2 -4
Chemical Shift (ppm)

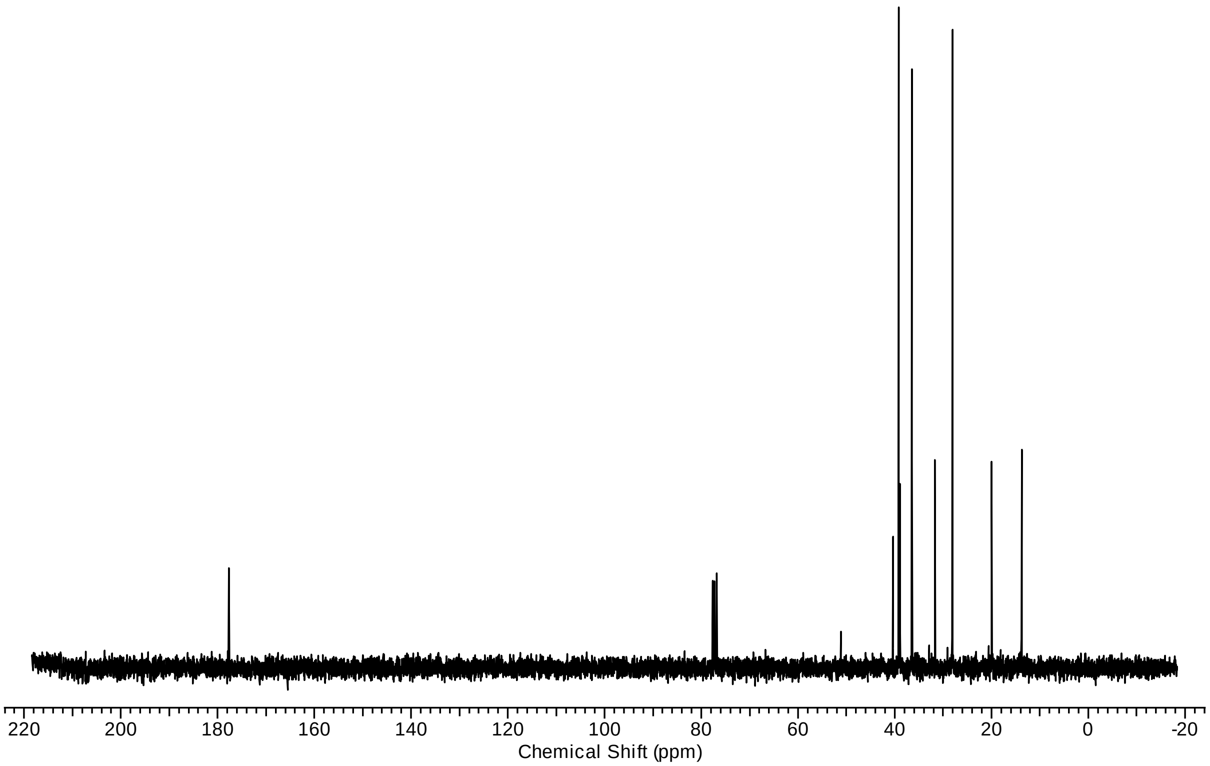
220 200 180 160 140 120 100 80 60 40 20 0 -20
Chemical Shift (ppm)

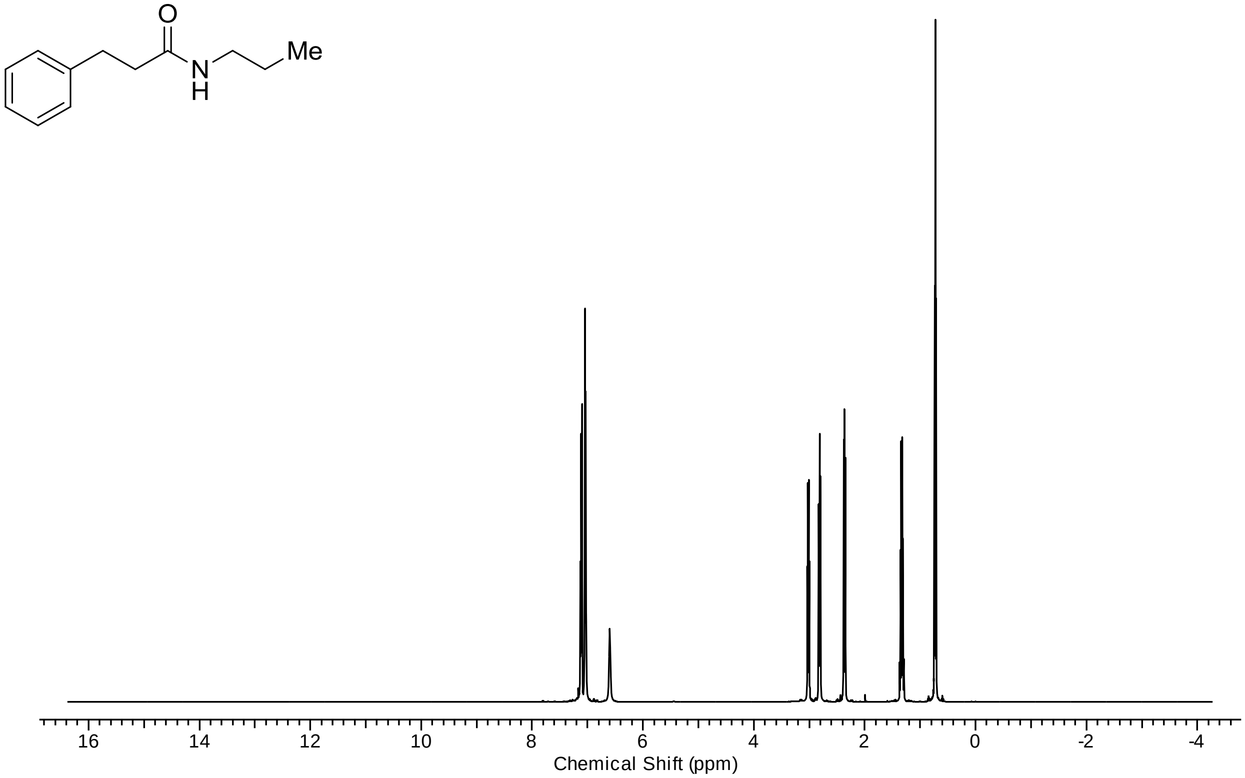
O
N
H
Me
16
14
12
10
8
6
4
2
0
-2
-4
Chemical Shift (ppm)

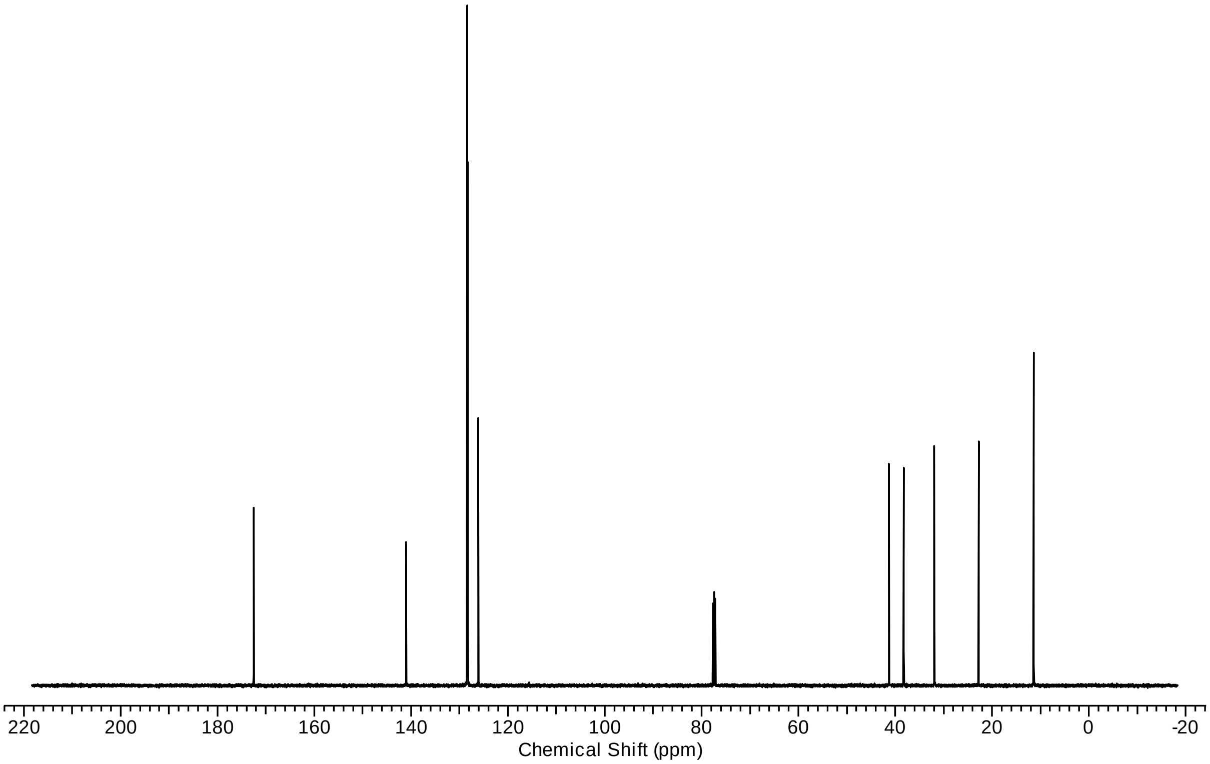
220
200
180
160
140
120
100
80
60
40
20
0
-20
Chemical Shift (ppm)

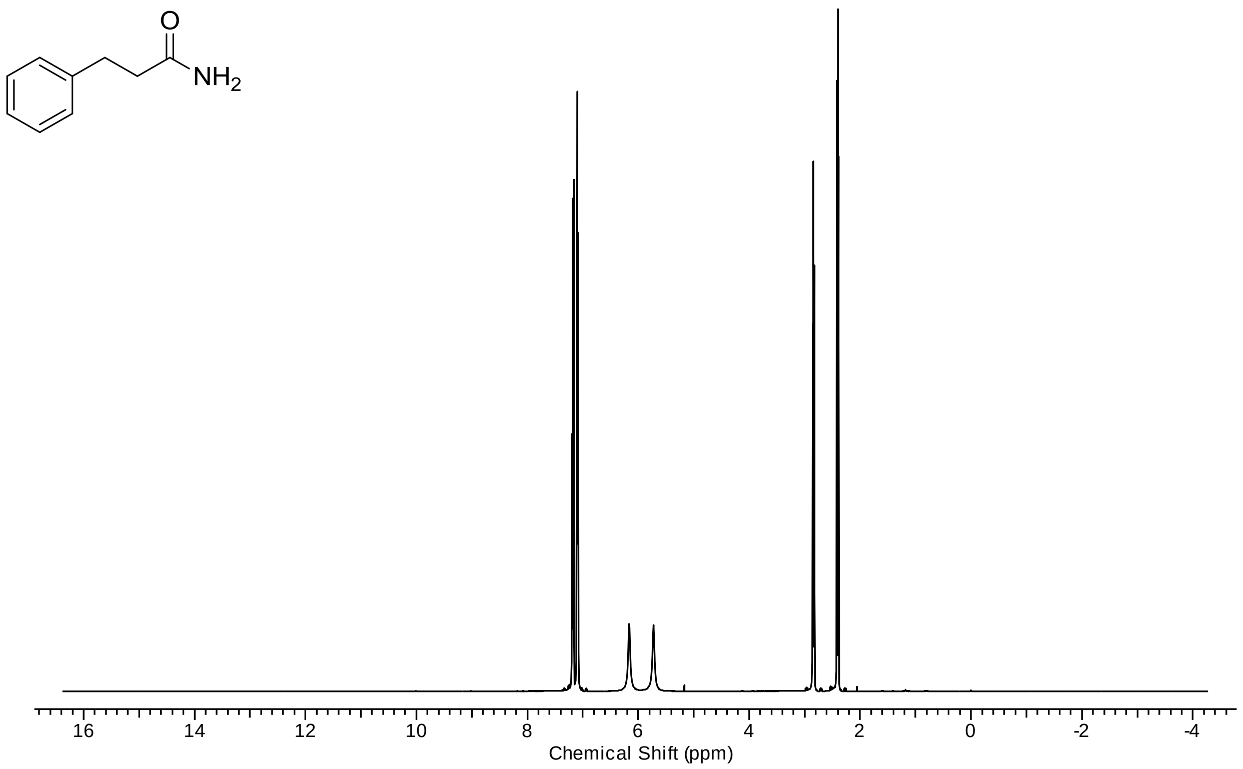
O
NH2
16 14 12 10 8 6 4 2 0 -2 -4
Chemical Shift (ppm)

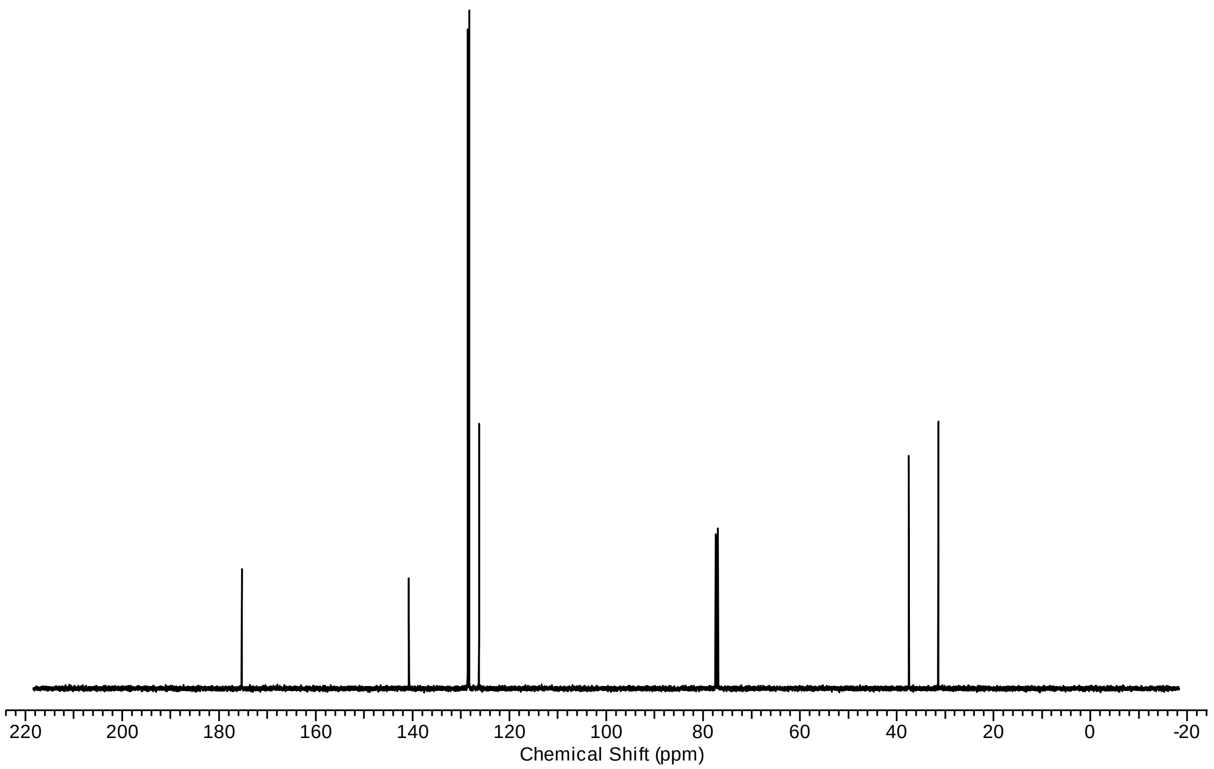
220 200 180 160 140 120 100 80 60 40 20 0 -20
Chemical Shift (ppm)

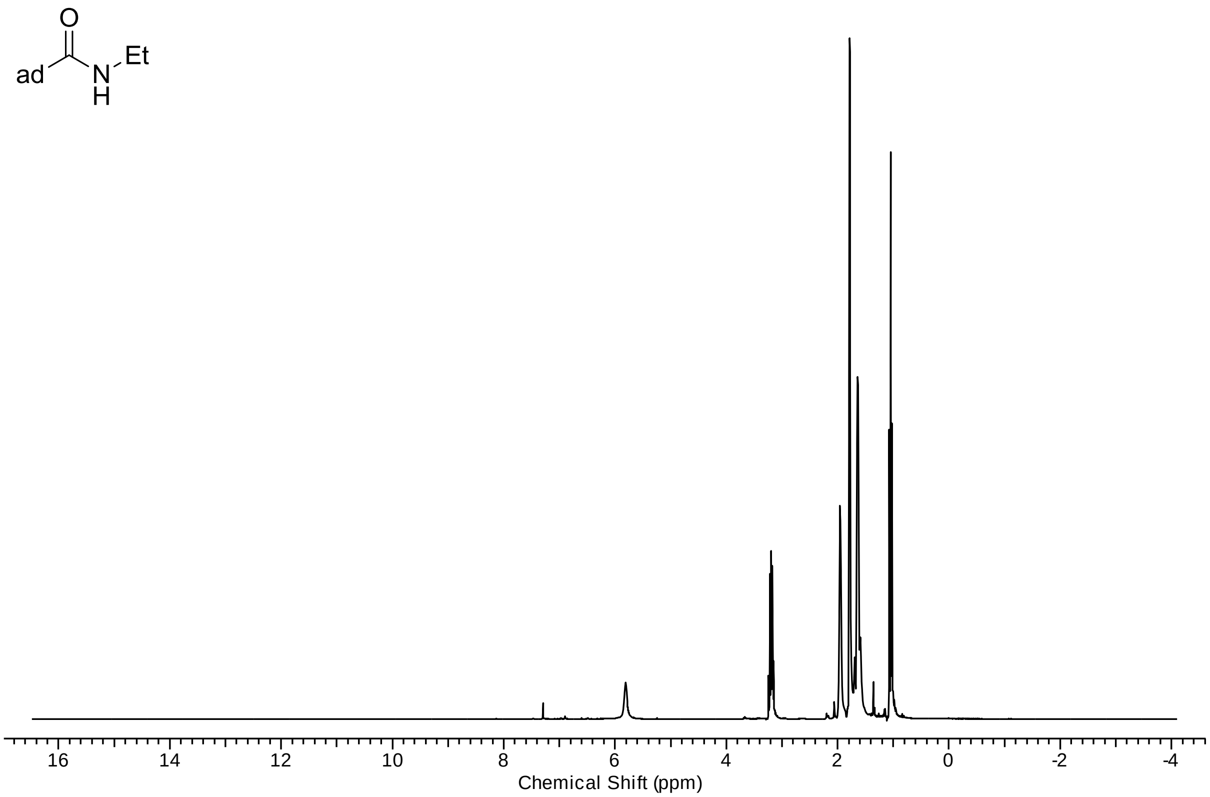
O
ad
N
Et
H
16
14
12
10
8
6
4
2
0
-2
-4
Chemical Shift (ppm)

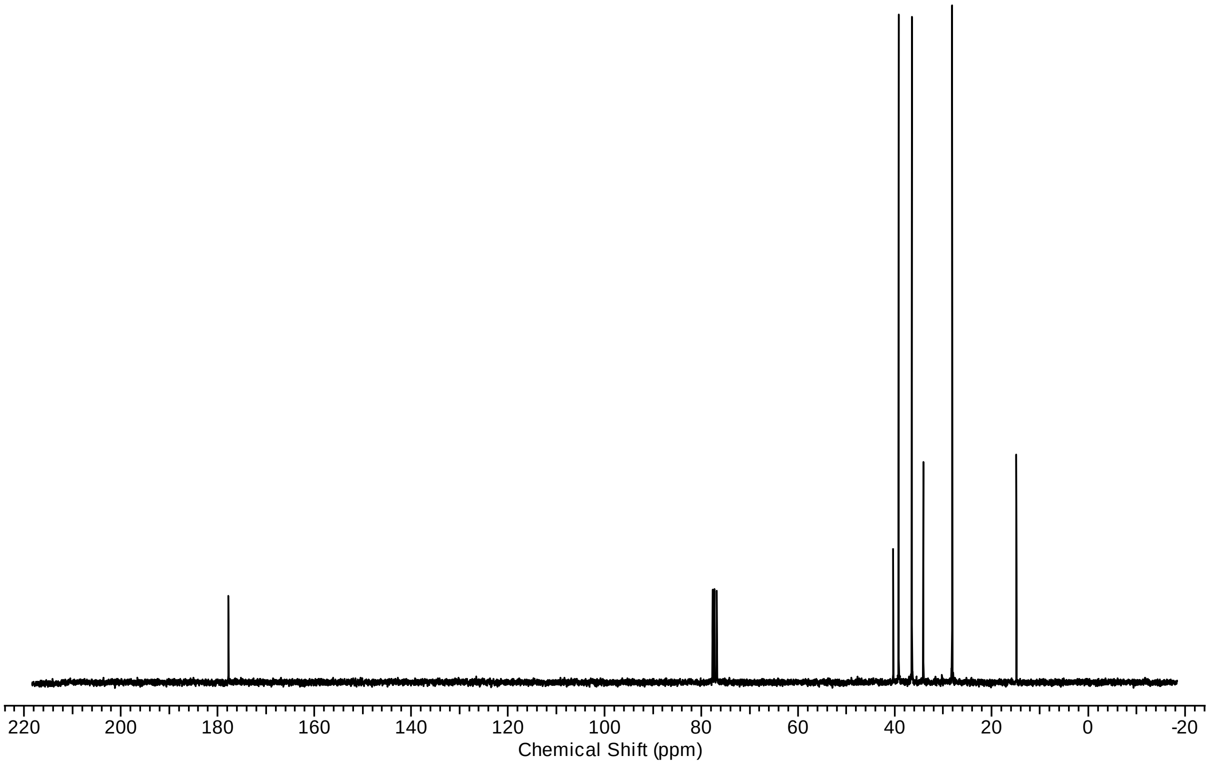
220
200
180
160
140
120
100
80
60
40
20
0
-20
Chemical Shift (ppm)

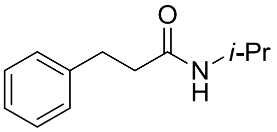
O
N
H
i-Pr

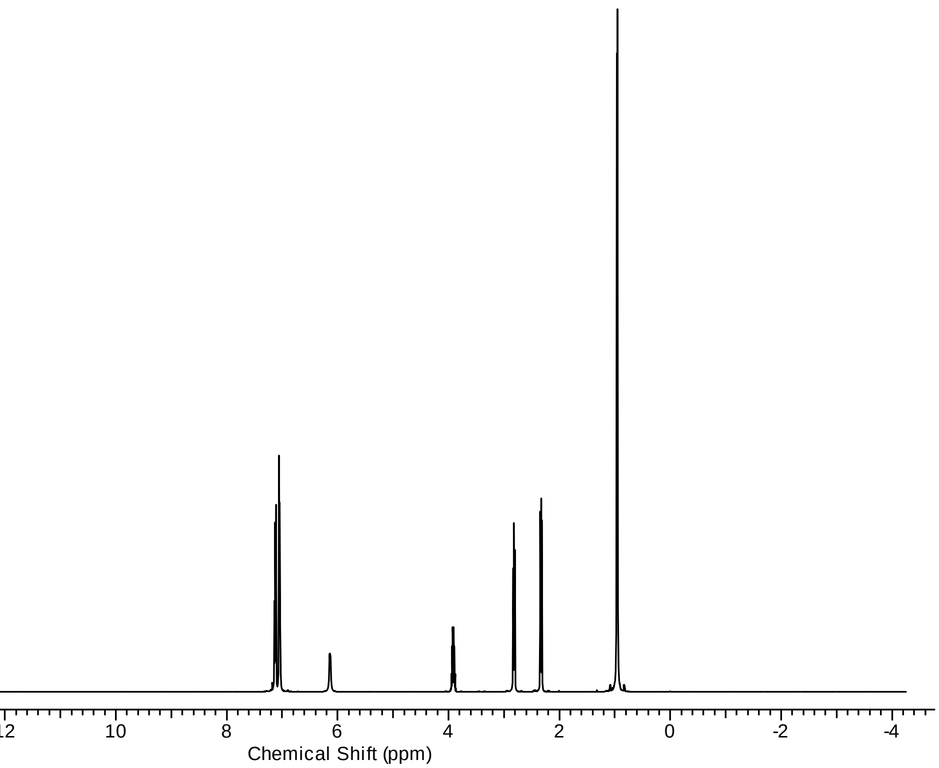
16 14 12 10 8 6 4 2 0 -2 -4
Chemical Shift (ppm)

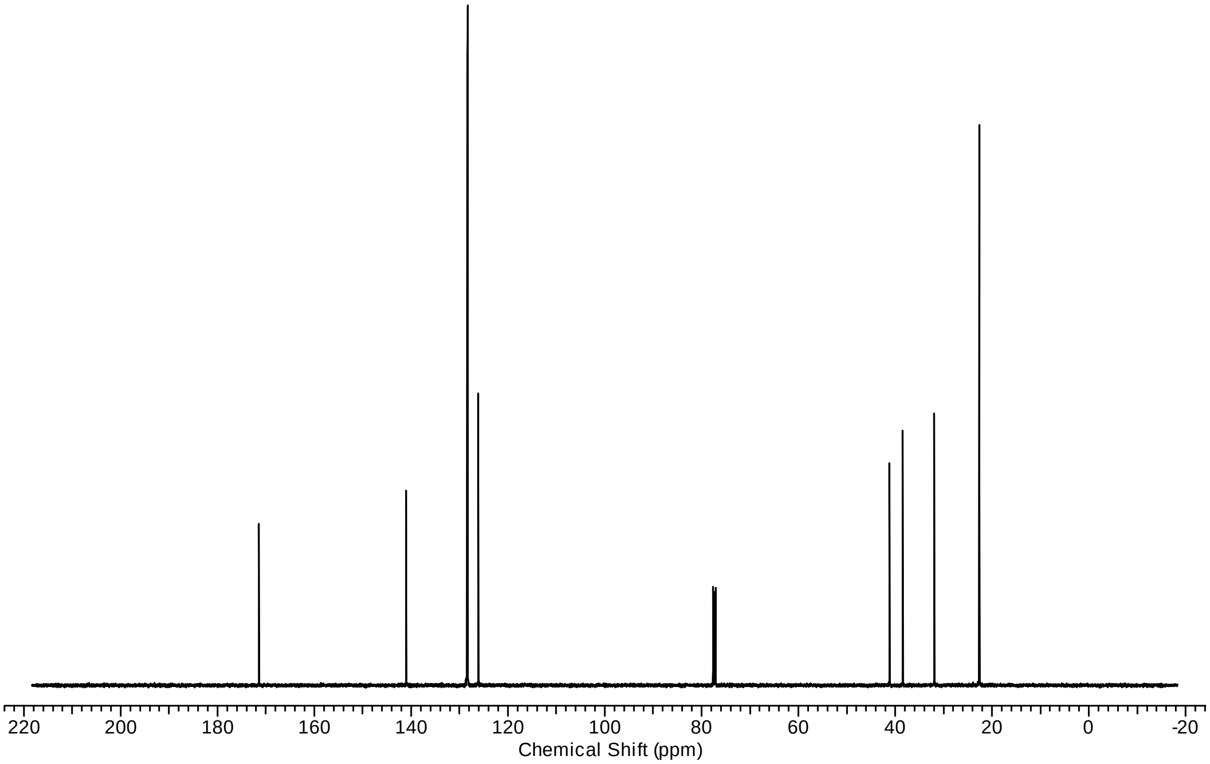
220 200 180 160 140 120 100 80 60 40 20 0 -20
Chemical Shift (ppm)

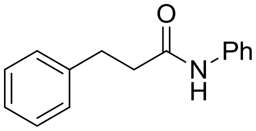
O
N
Ph
H

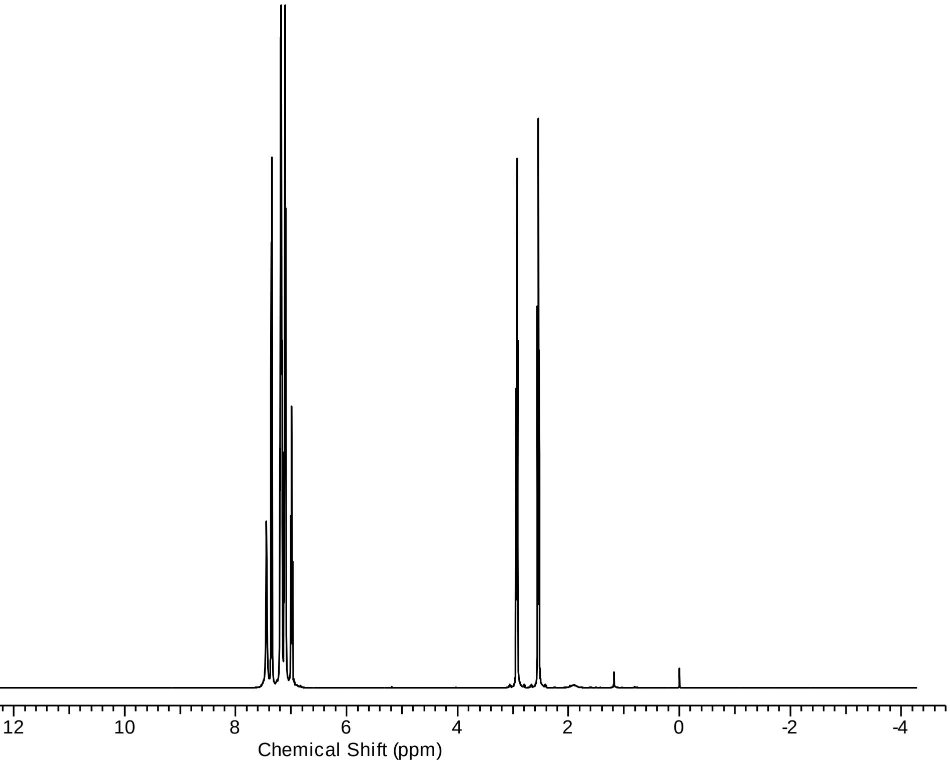
16
14
12
10
8
6
4
2
0
-2
-4
Chemical Shift (ppm)

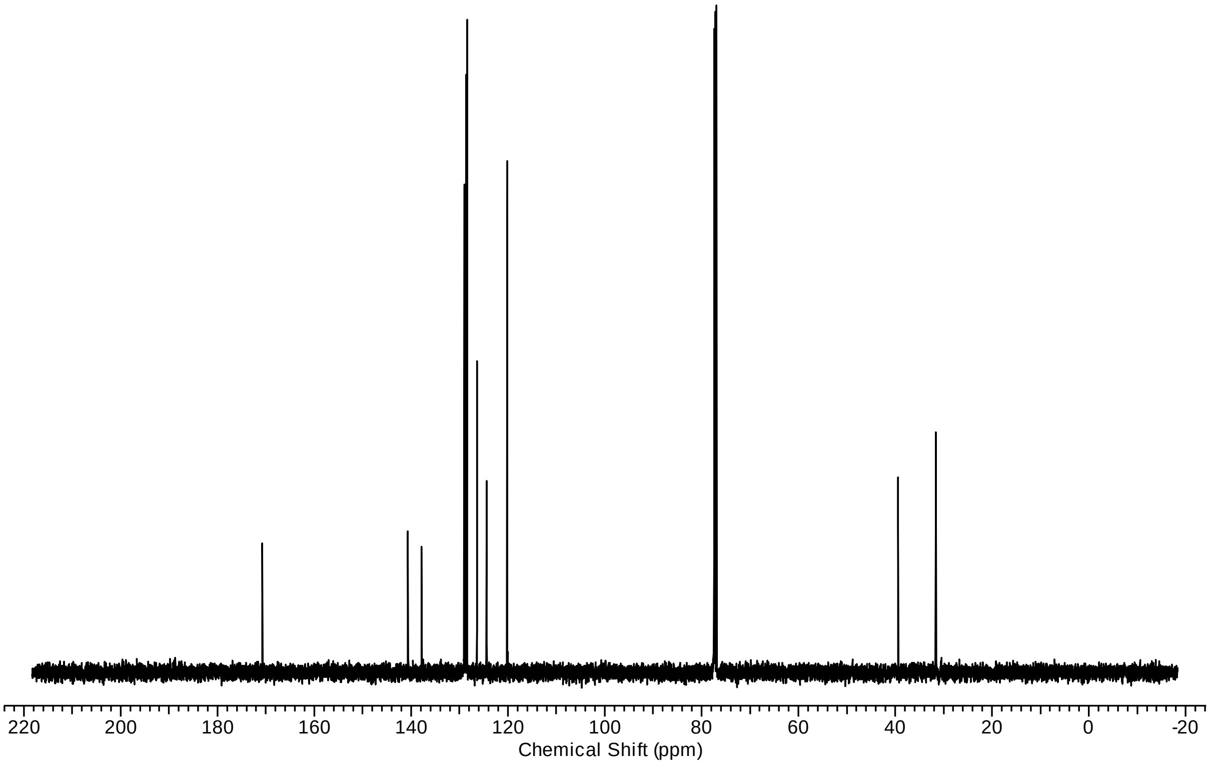
220
200
180
160
140
120
100
80
60
40
20
0
-20
Chemical Shift (ppm)

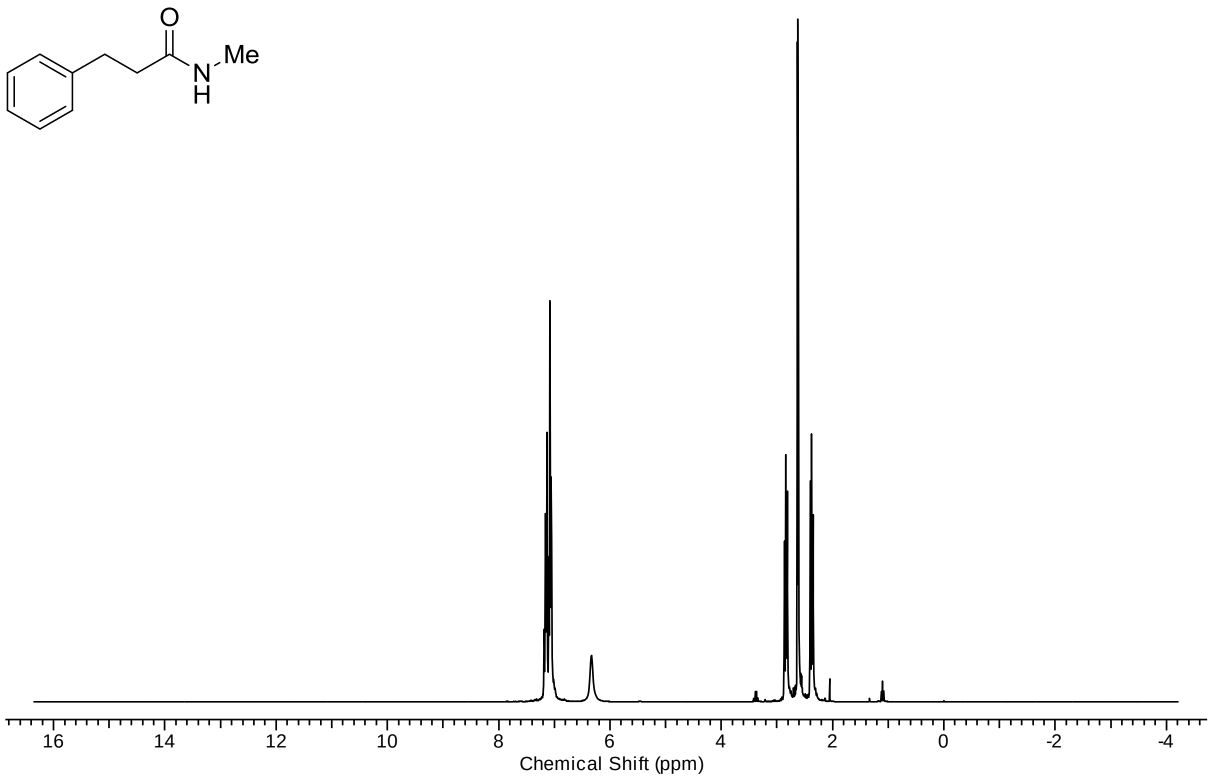
O
N
H
Me
16
14
12
10
8
6
4
2
0
-2
-4
Chemical Shift (ppm)

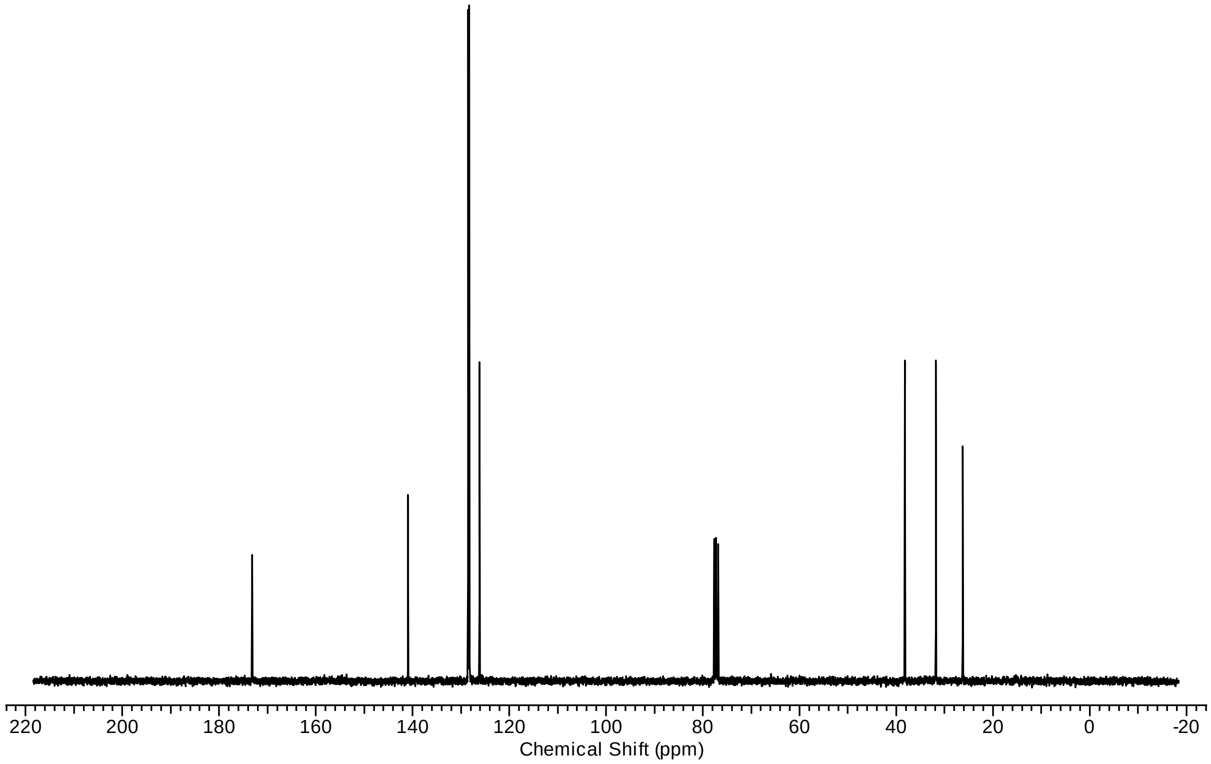
220
200
180
160
140
120
100
80
60
40
20
0
-20
Chemical Shift (ppm)

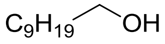
C9H19
OH

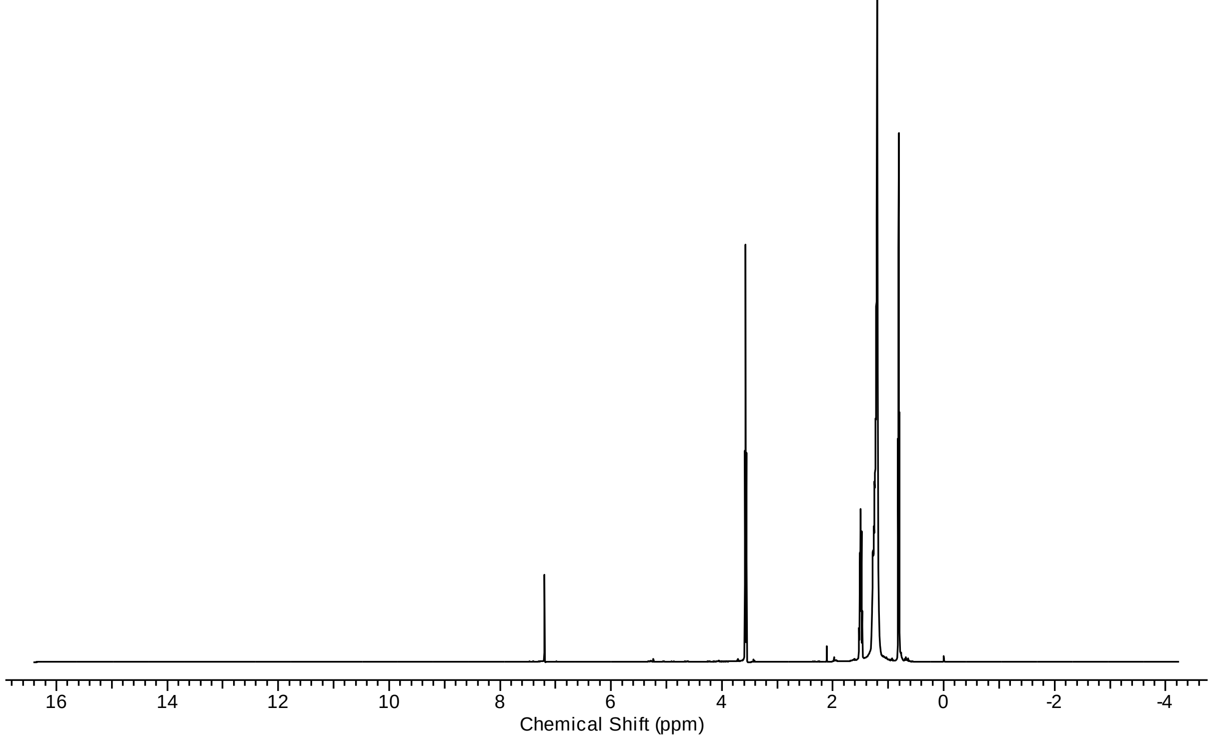
16
14
12
10
8
6
4
2
0
-2
-4
Chemical Shift (ppm)

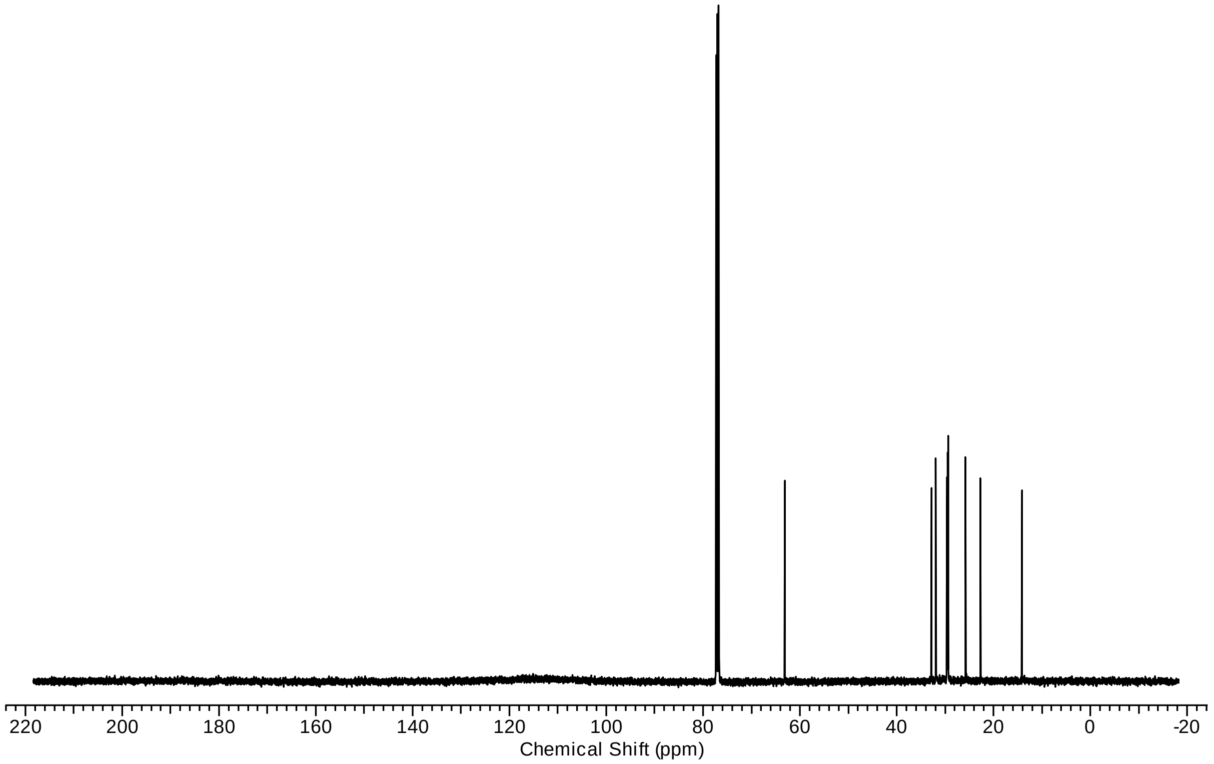
220
200
180
160
140
120
100
80
60
40
20
0
-20
Chemical Shift (ppm)

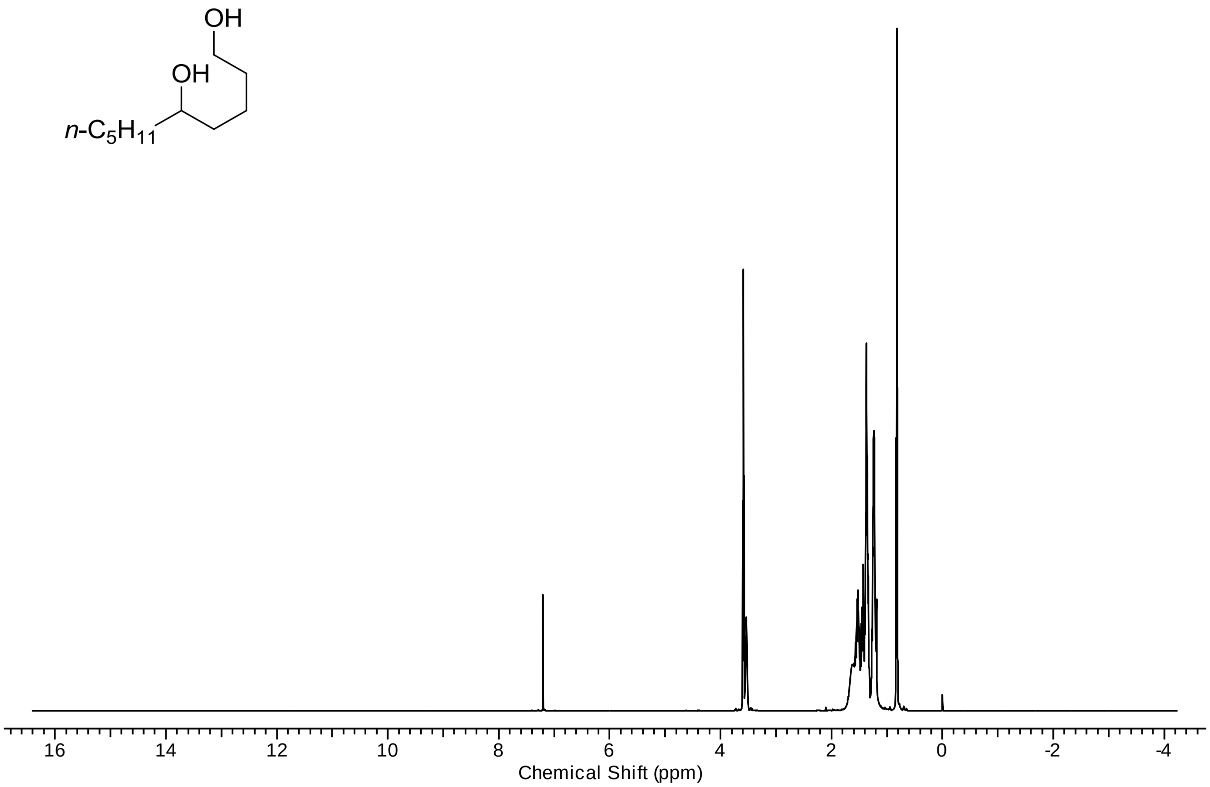
OH
OH
n-C5H11
16 14 12 10 8 6 4 2 0 -2 -4
Chemical Shift (ppm)

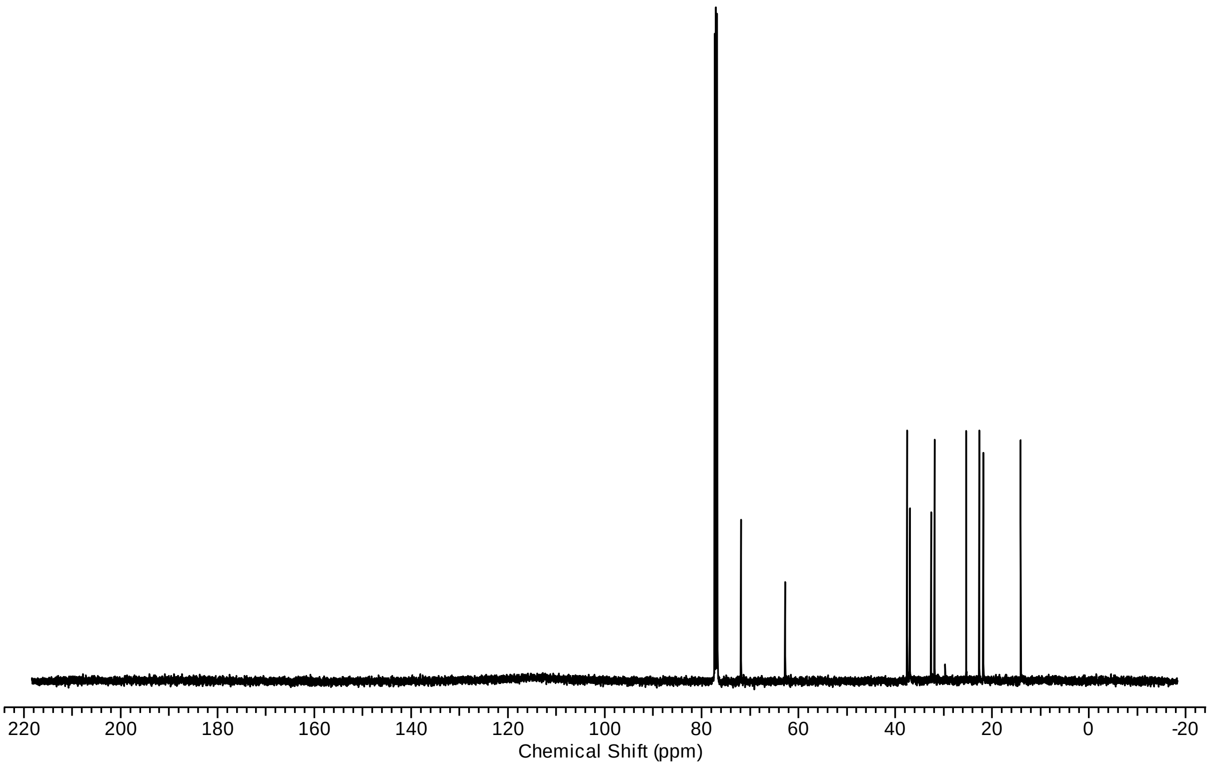
220 200 180 160 140 120 100 80 60 40 20 0 -20
Chemical Shift (ppm)

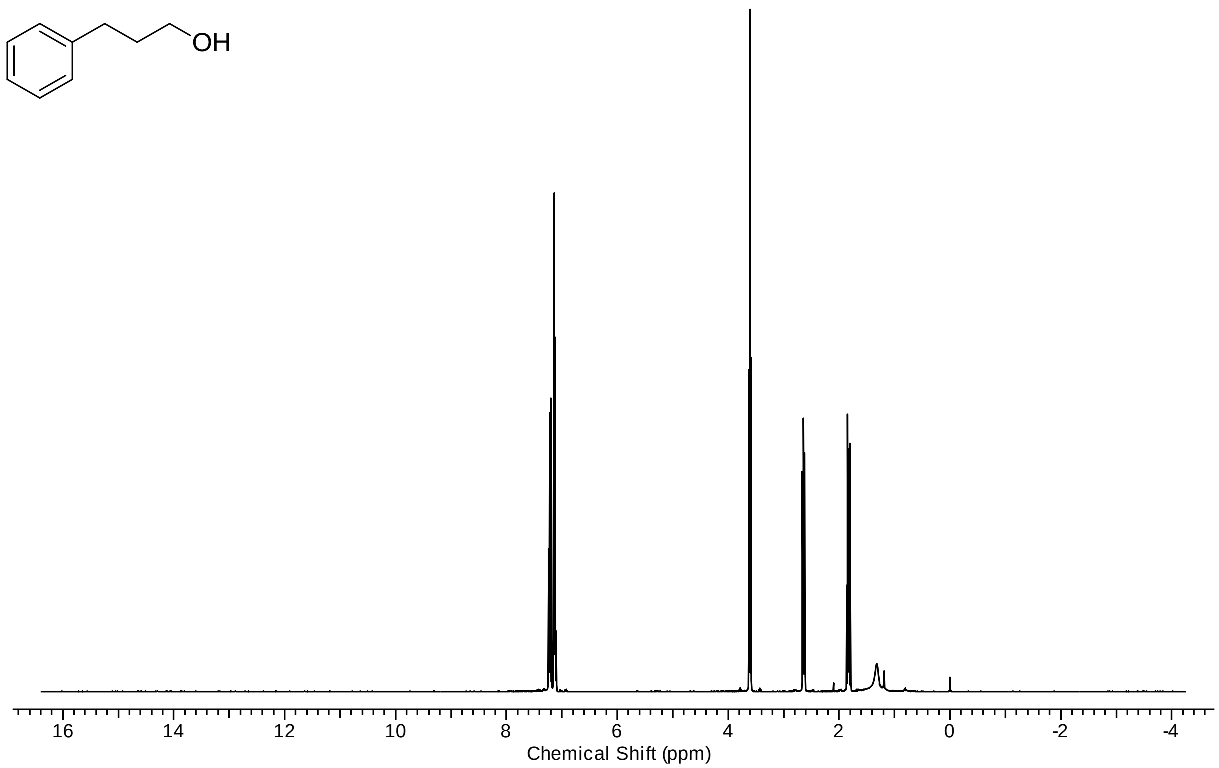
OH
16
14
12
10
8
6
4
2
0
-2
-4
Chemical Shift (ppm)

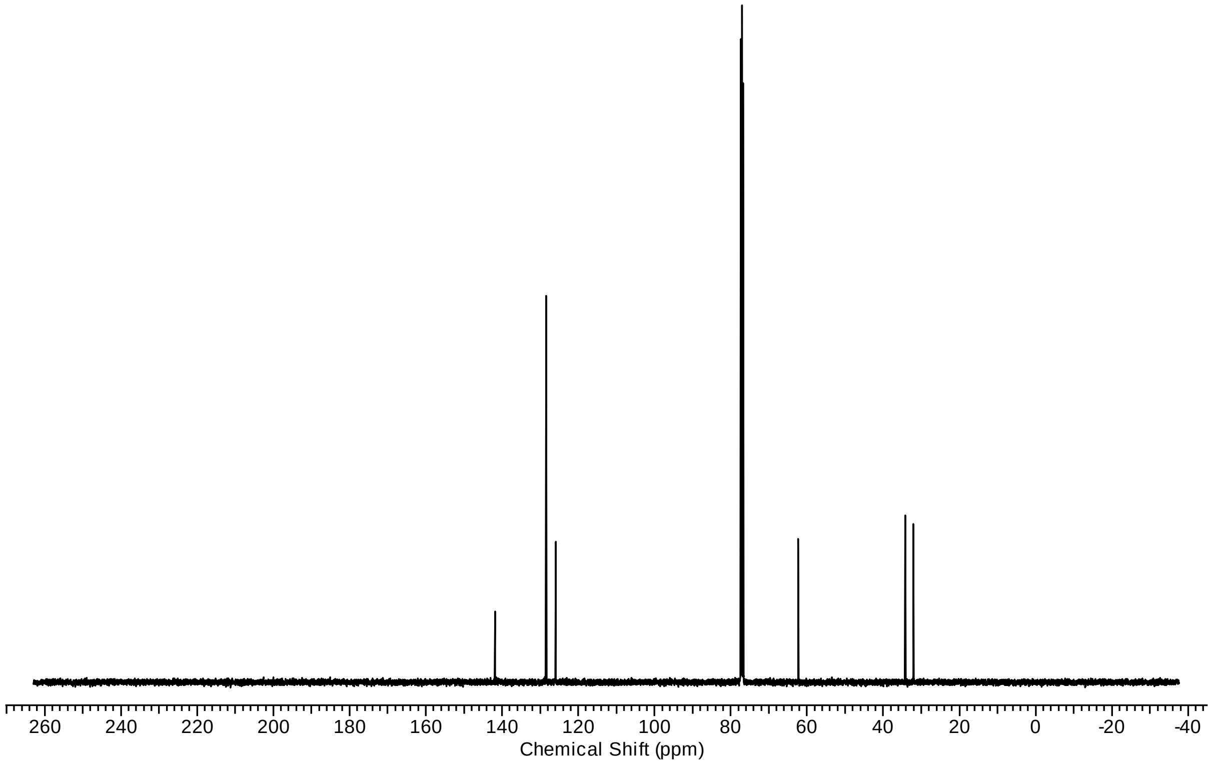
260
240
220
200
180
160
140
120
100
80
60
40
20
0
-20
-40
Chemical Shift (ppm)

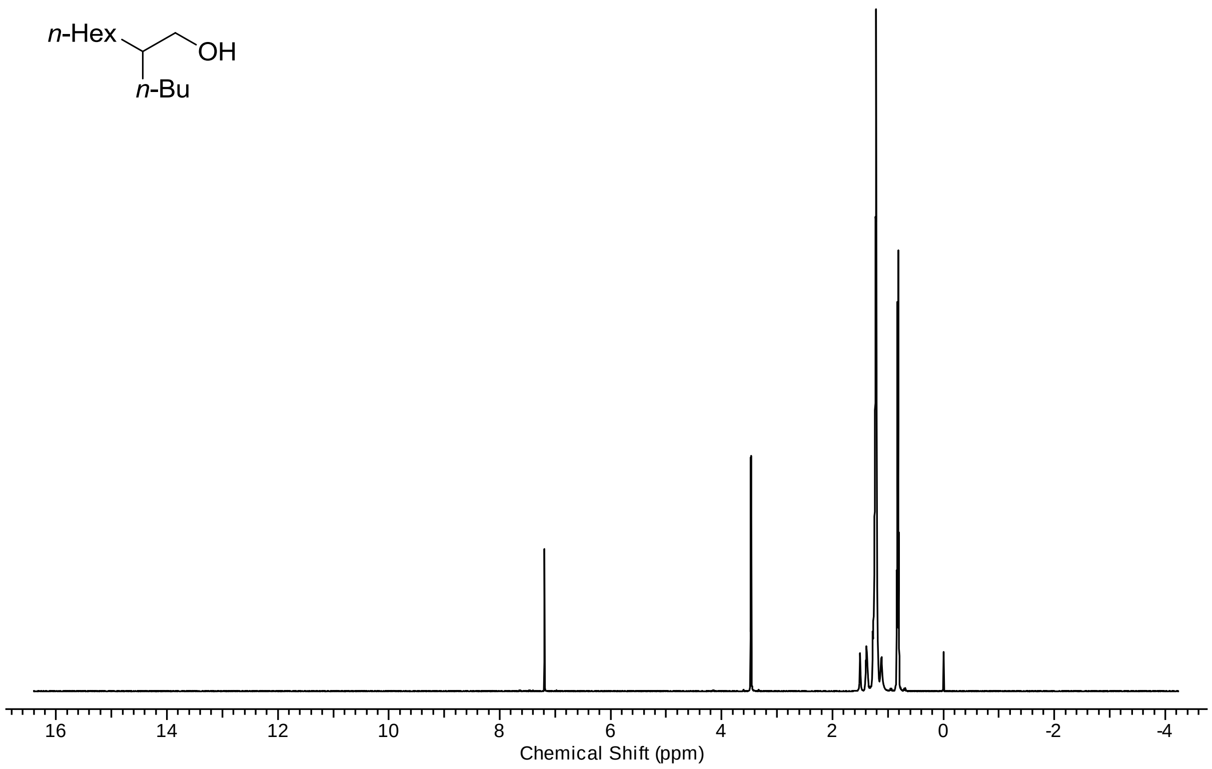
n-Hex
OH
n-Bu
16
14
12
10
8
6
4
2
0
-2
-4
Chemical Shift (ppm)

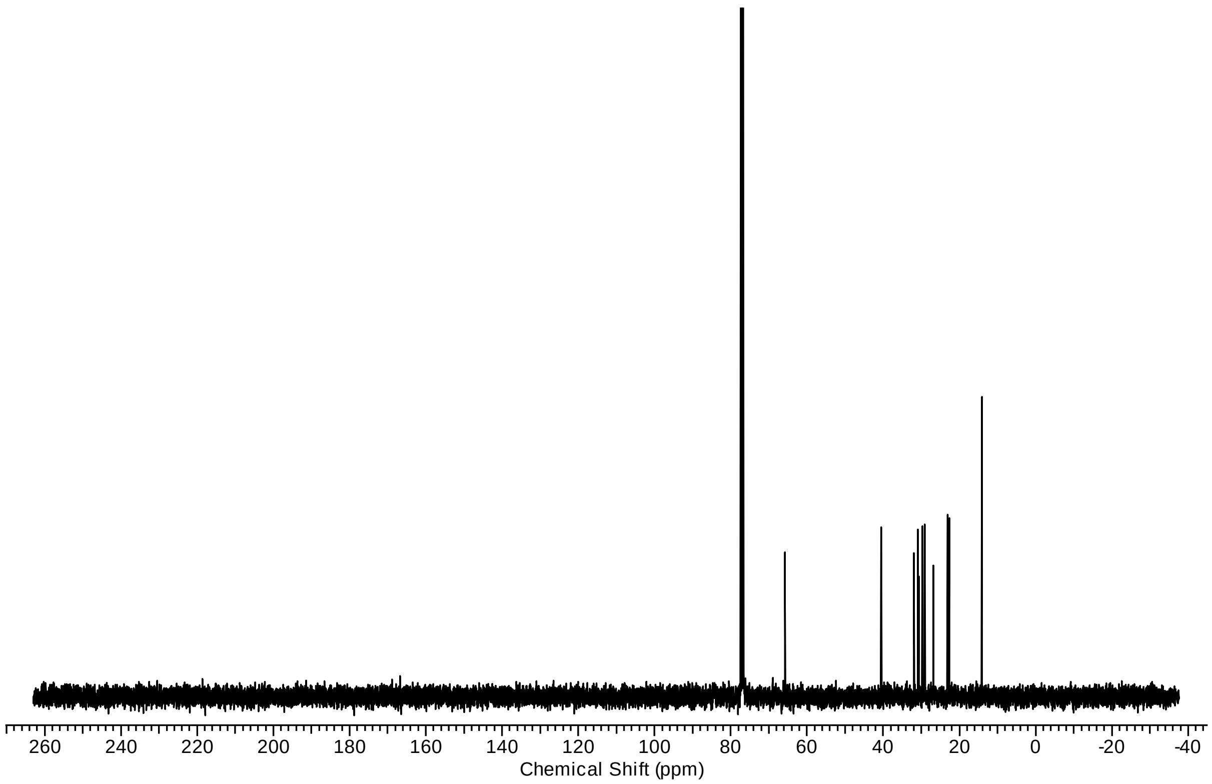
260
240
220
200
180
160
140
120
100
80
60
40
20
0
-20
-40
Chemical Shift (ppm)

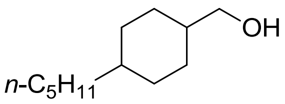
OH
n-C5H11

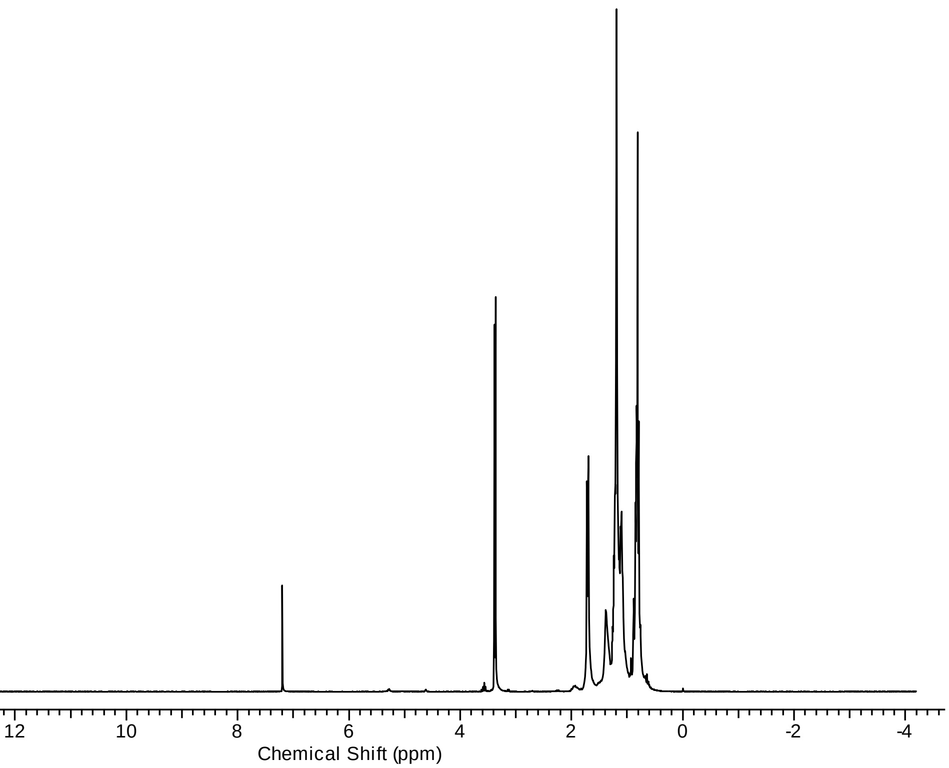
16 14 12 10 8 6 4 2 0 -2 -4
Chemical Shift (ppm)

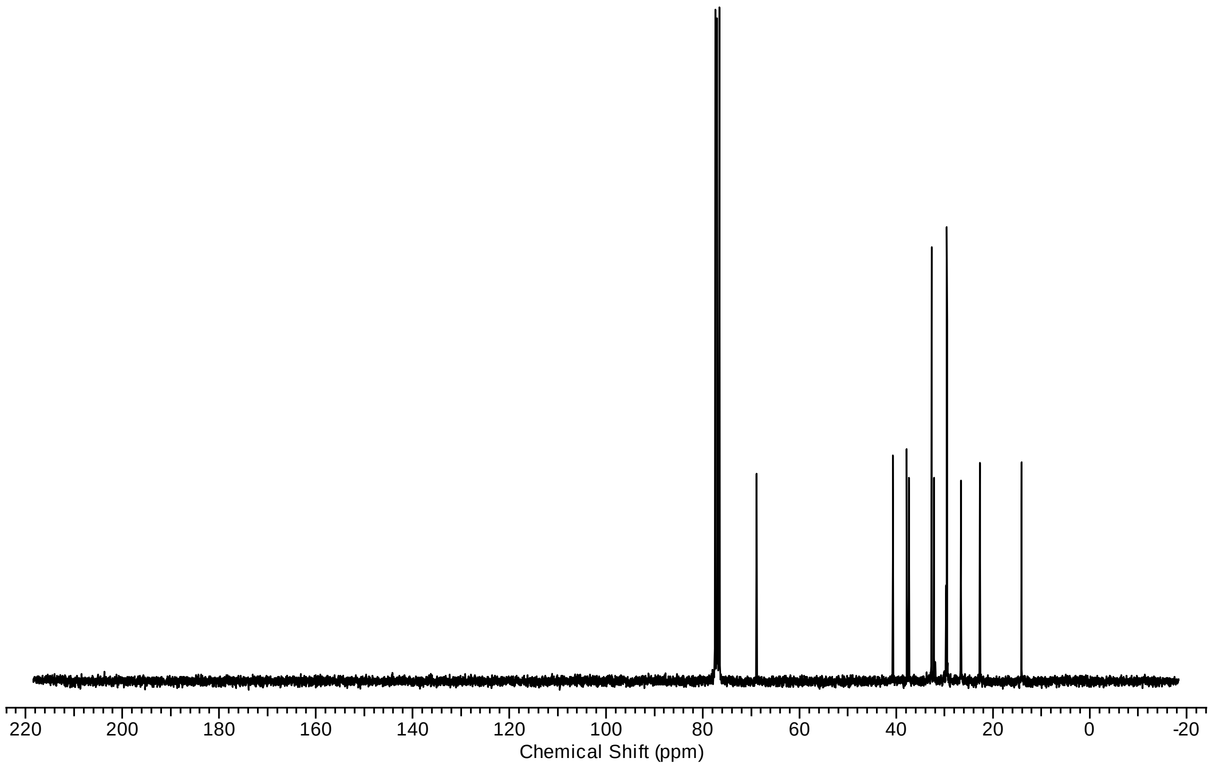
220 200 180 160 140 120 100 80 60 40 20 0 -20
Chemical Shift (ppm)

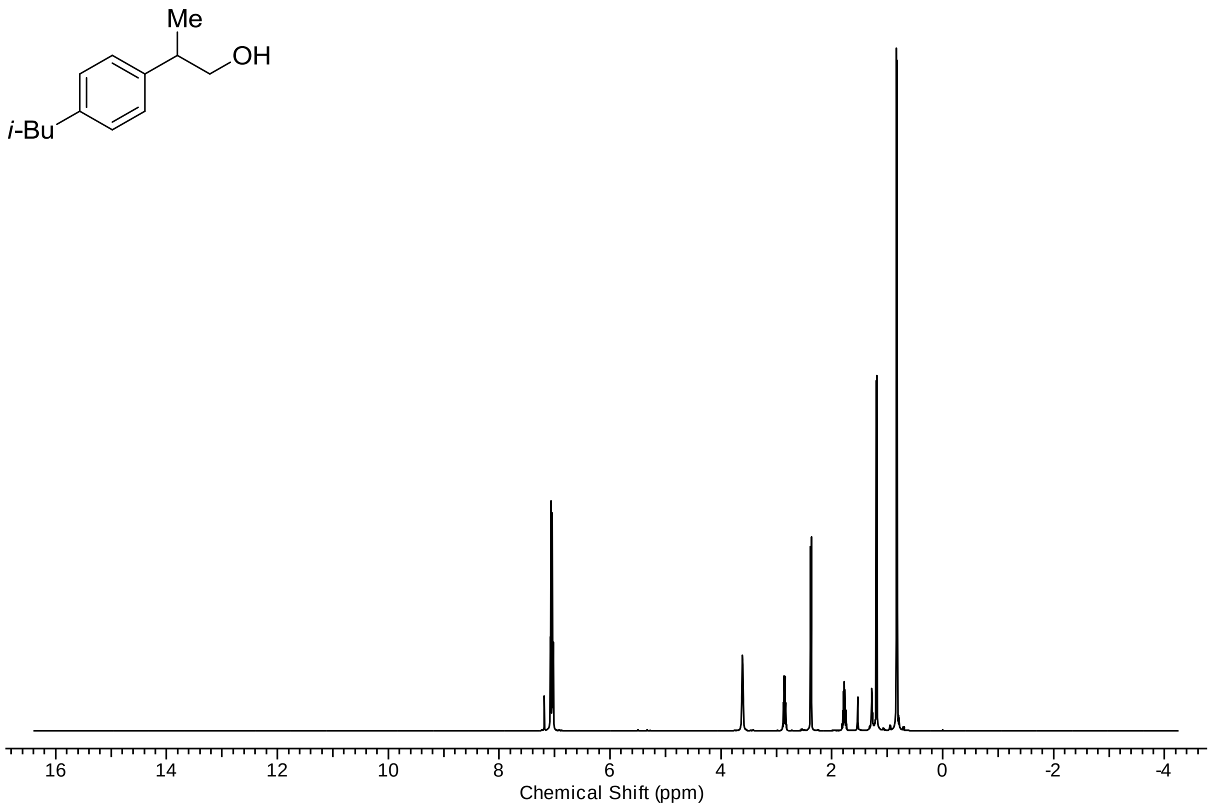
Me
OH
i-Bu
16 14 12 10 8 6 4 2 0 -2 -4
Chemical Shift (ppm)

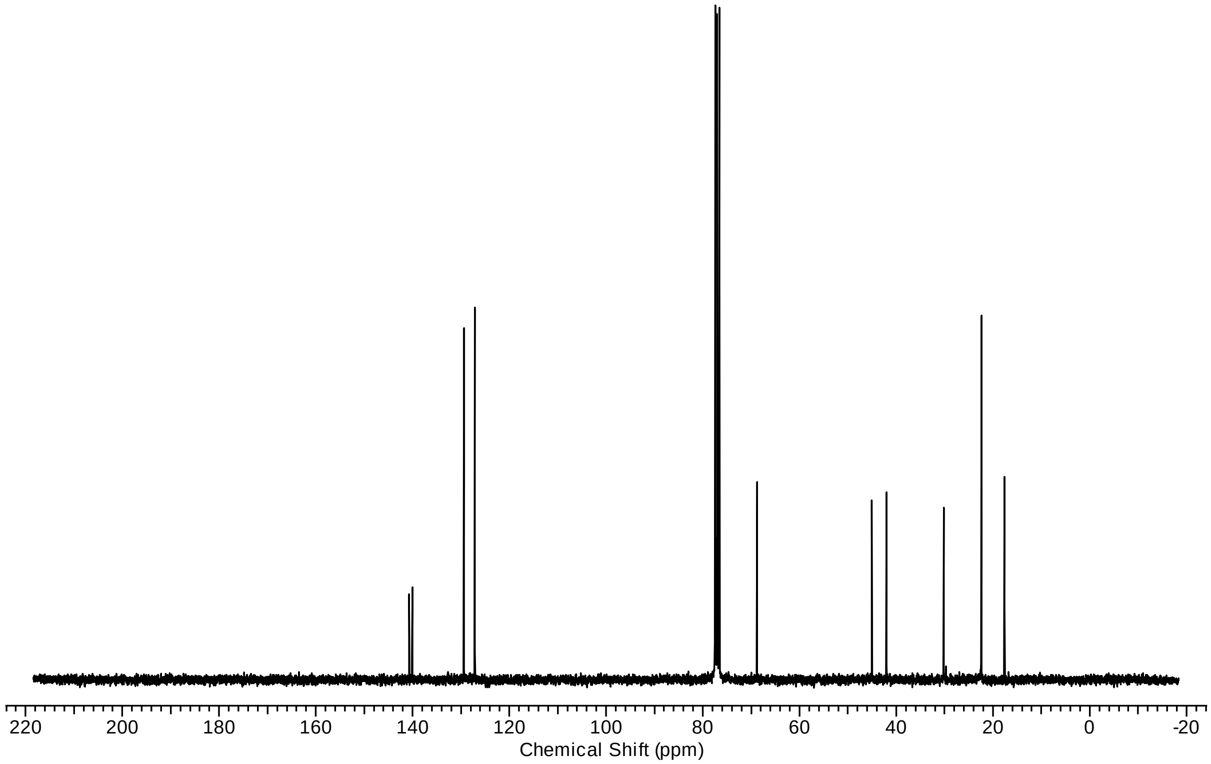
220 200 180 160 140 120 100 80 60 40 20 0 -20
Chemical Shift (ppm)

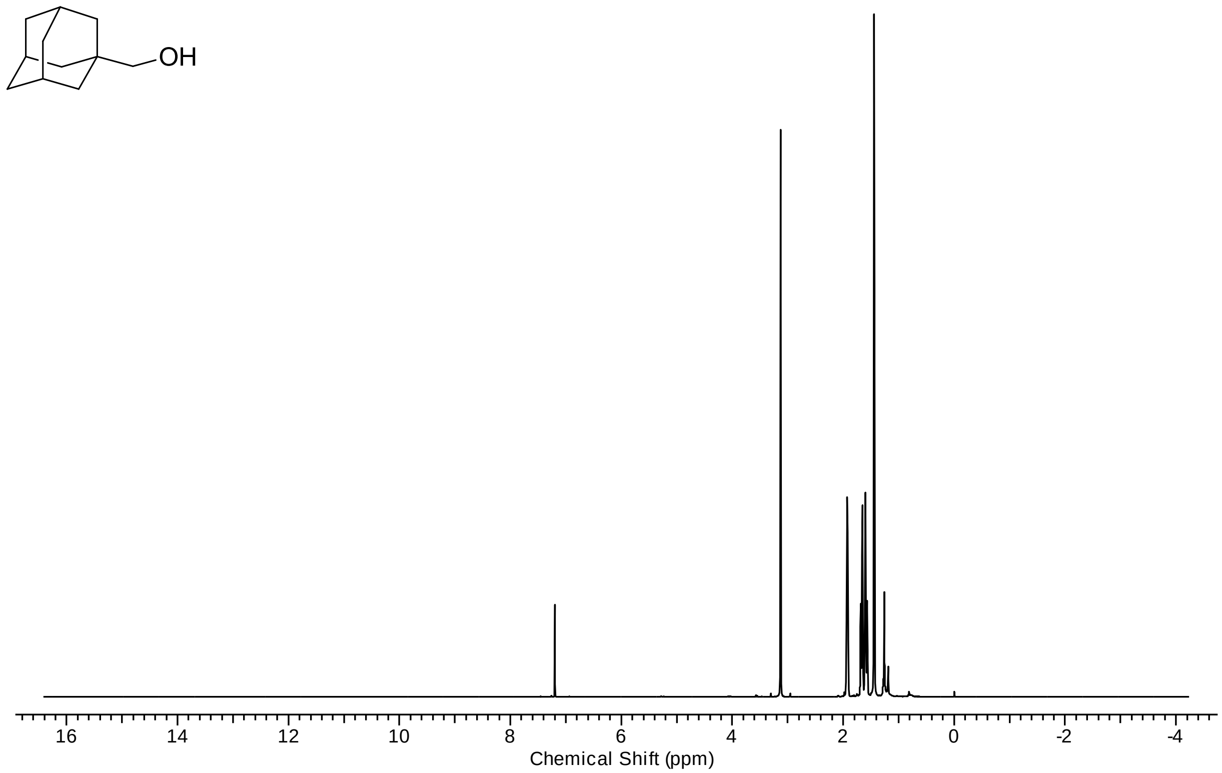
OH
16 14 12 10 8 6 4 2 0 -2 -4
Chemical Shift (ppm)

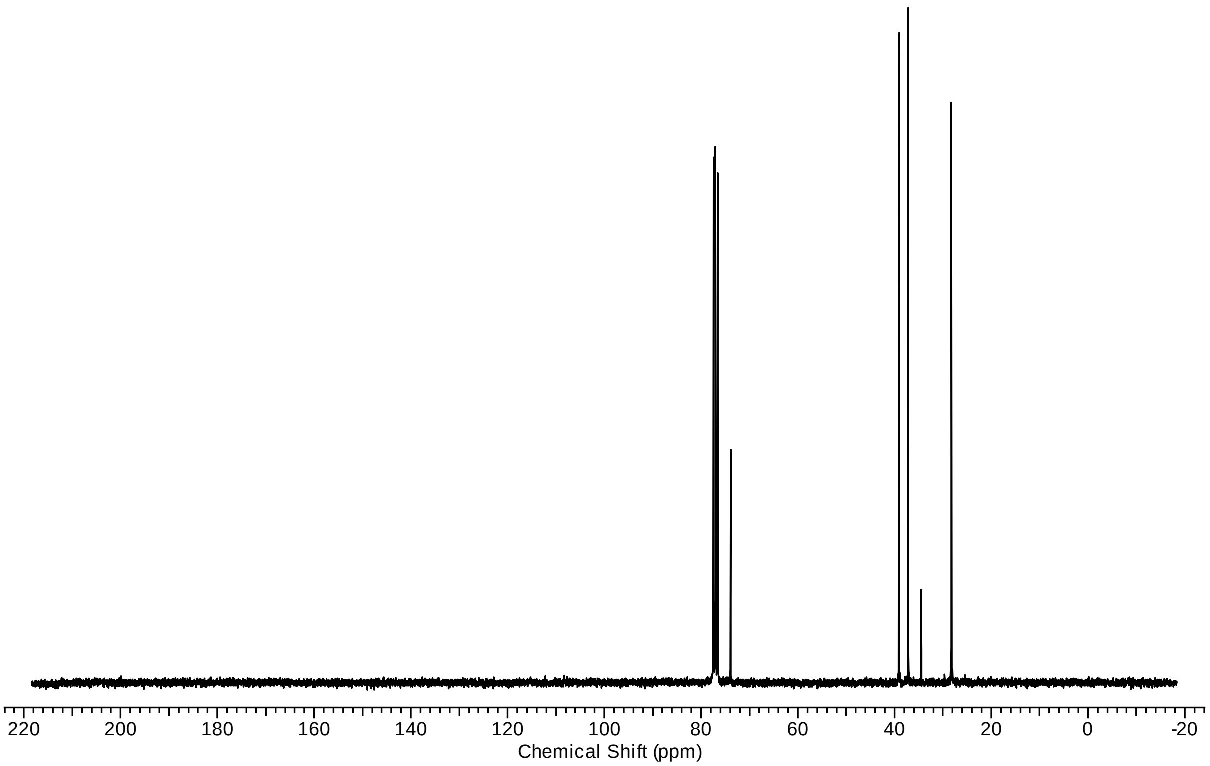
220 200 180 160 140 120 100 80 60 40 20 0 -20
Chemical Shift (ppm)

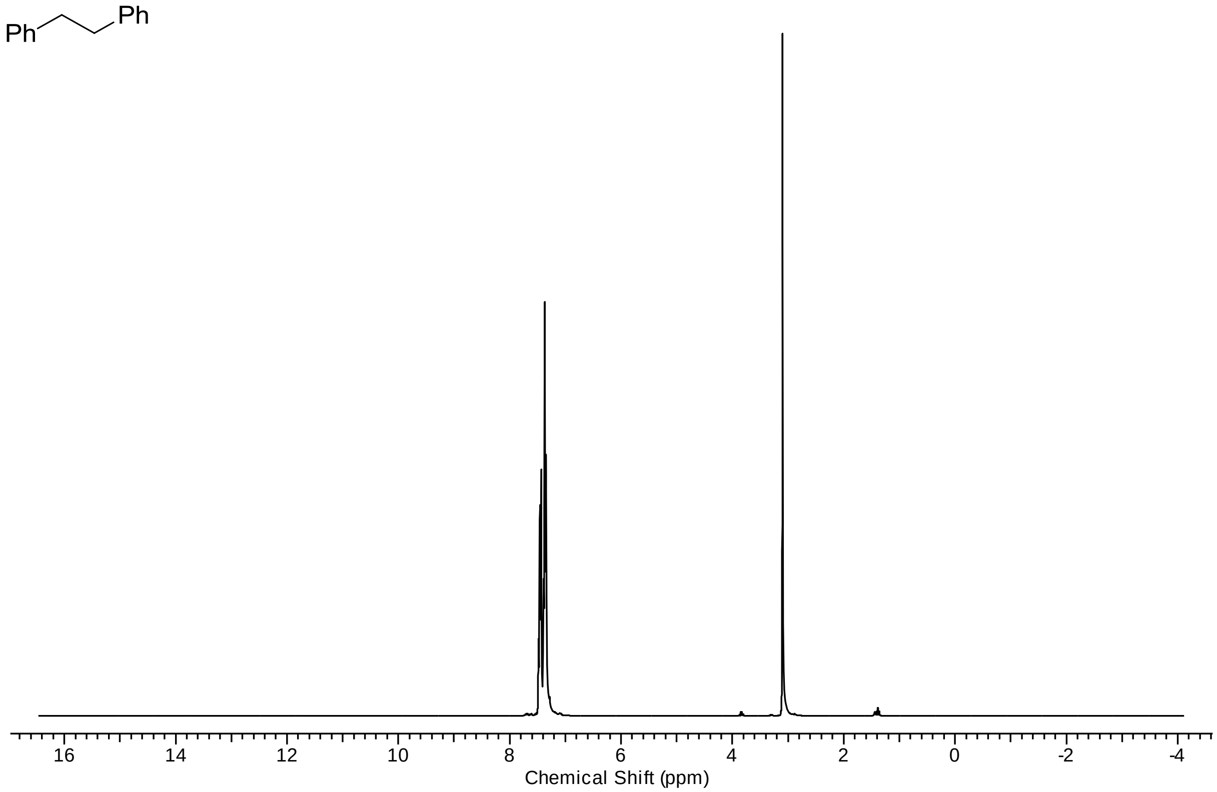
Ph
Ph
16
14
12
10
8
6
4
2
0
-2
-4
Chemical Shift (ppm)

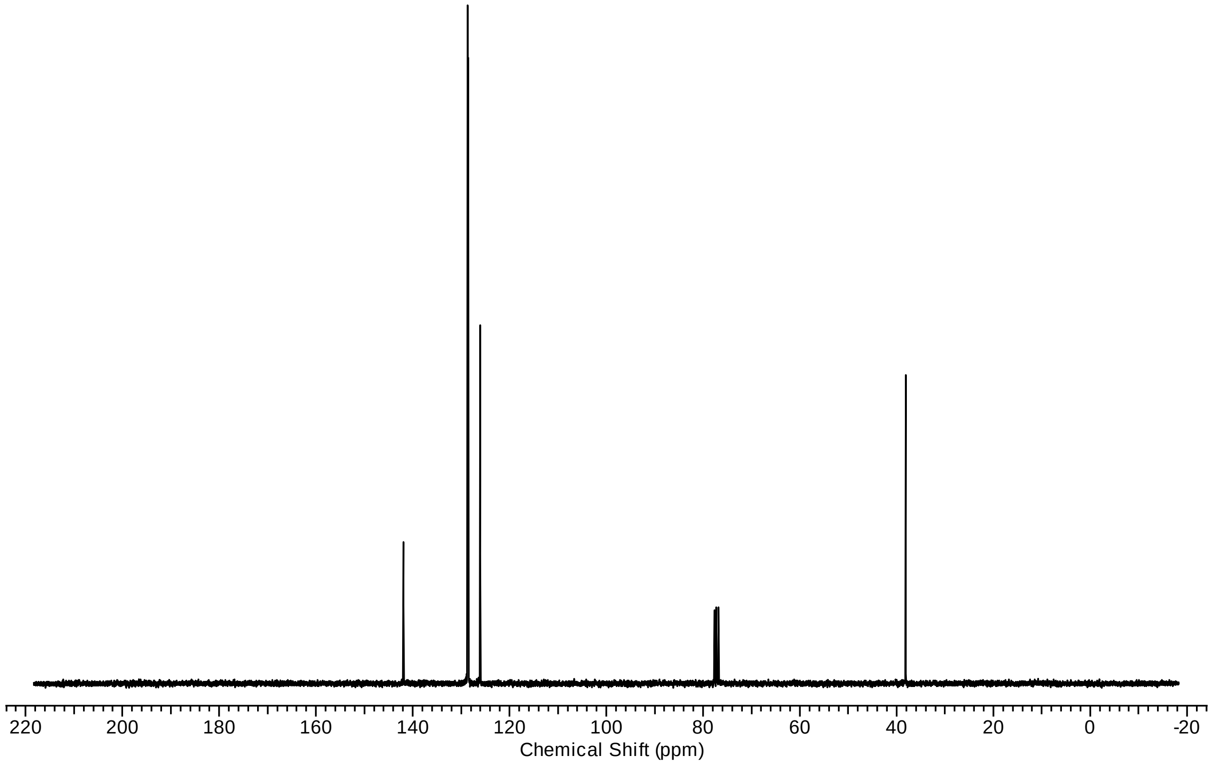
220
200
180
160
140
120
100
80
60
40
20
0
-20
Chemical Shift (ppm)

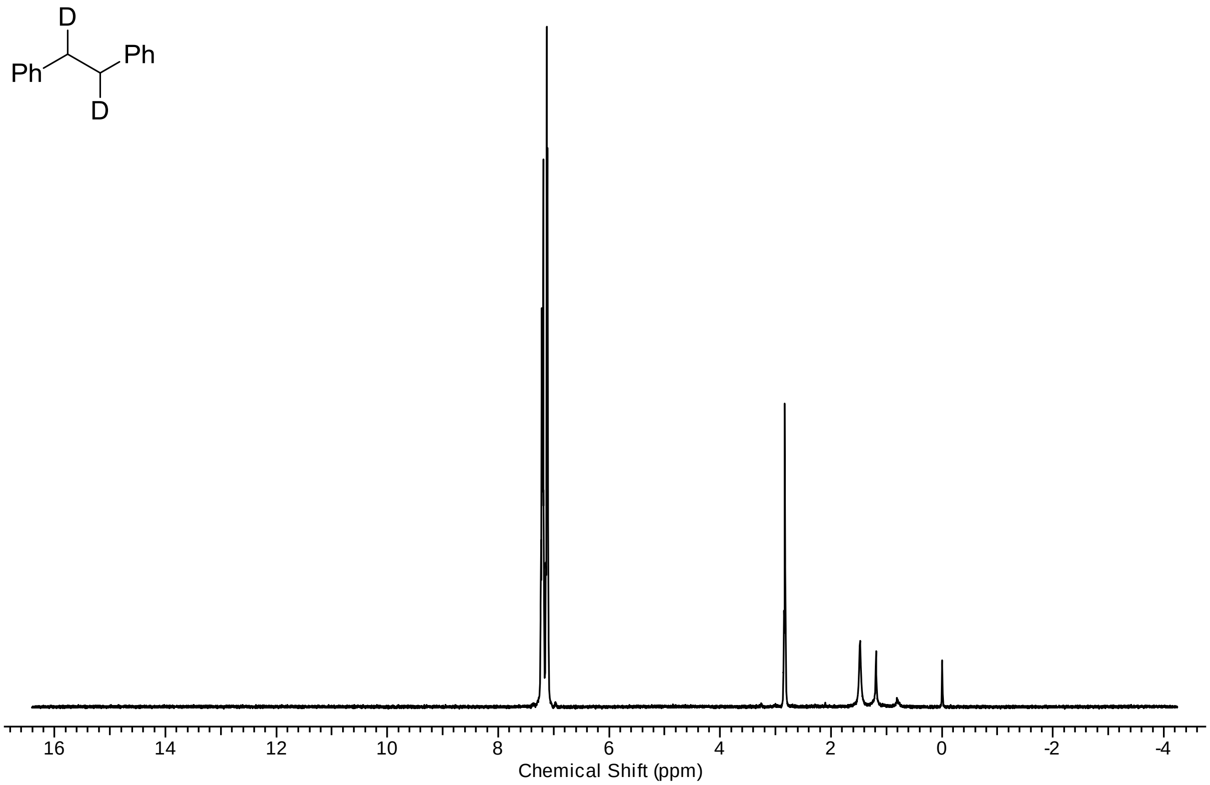
D
Ph
Ph
D
16
14
12
10
8
6
4
2
0
-2
-4
Chemical Shift (ppm)

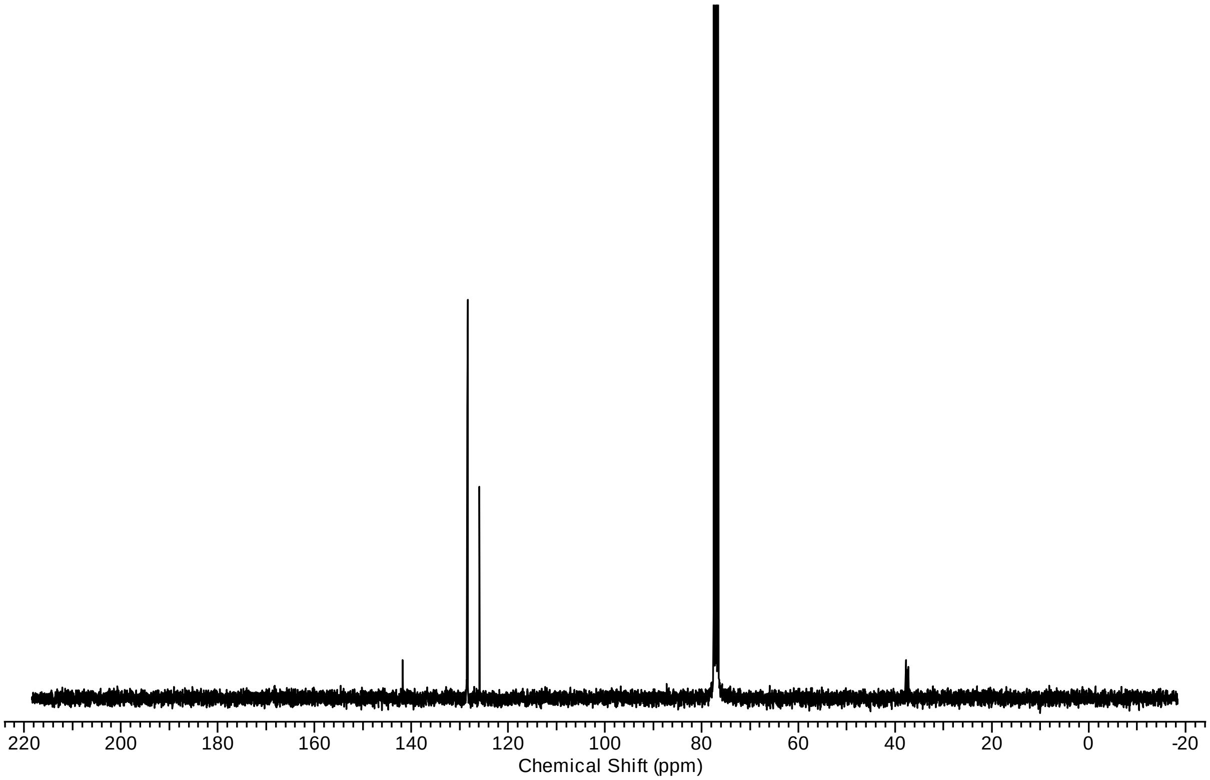
220
200
180
160
140
120
100
80
60
40
20
0
-20
Chemical Shift (ppm)
